# Huntington’s disease vasculopathy is barrier-type-selective and arteriolar-dominant with spatially-coupled astrogliosis and reversible by S1pr1 stimulation

**DOI:** 10.1038/s41467-026-77474-4

**Published:** 2026-09-12

**Authors:** Krzysztof Kucharz, Rana Soylu-Kucharz, Martine Saint-Pierre, Melanie Alpaugh, Filippa Liliendahl Qvist, Maria Björkqvist, Niels Henning Skotte, Francesca Cicchetti, Martin Johannes Lauritzen

**Affiliations:** 1 Department of Neuroscience, University of Copenhagen, Copenhagen, Denmark; 2 Department of Experimental Medical Science, Lund University, Lund, Sweden; 3 Centre de Recherche du CHU de Québec, Axe Neurosciences, Québec City, QC Canada; 4 Département de Psychiatrie & Neurosciences, Université Laval, Québec City, QC Canada; 5 Department of Molecular and Cellular Biology, University of Guelph, Guelph, ON Canada; 6 Department of Drug Design and Pharmacology, University of Copenhagen, Copenhagen, Denmark; 7 NNF Center for Protein Research, Faculty of Health Sciences, University of Copenhagen, Copenhagen, Denmark

**Keywords:** Blood-brain barrier, Huntington's disease, Neuro-vascular interactions

## Abstract

Brain vascular barriers are dysfunctional in many neurological disorders, including Huntington’s disease (HD), but which vessels are affected and how remains unclear. Using in vivo two-photon microscopy in R6/2 HD-model mice, we reveal a striking divergence in barrier dysfunction. Surface vessels of the blood-cerebrospinal fluid barrier (BCSFB) drive pathology in HD by paracellular leakage, while parenchymal vessels forming the blood-brain barrier (BBB) exhibit increased vesicular transport and adsorptive-mediated transcytosis (AMT), with arterioles representing the most critically impacted vascular segment. The pathology of both barriers was spatially-matched with astrocyte activation pattern localized to affected vessels both in R6/2 mice and in brains of HD patients. Sphingosine-1-phosphate receptor 1 (S1pr1) agonist treatment reversed BCSFB leakage and partially restored BBB function, selectively reducing AMT in arterioles, with no effect in capillaries and venules. These findings redefine HD vascular pathology, demonstrating heterogeneous, vessel-type-specific barrier failure beyond generalized “disruption,” uncovering mechanistic vulnerabilities and the therapeutic potential of S1pr1 modulation.

## Abstract (web summary)

Here the authors map Huntington’s pathology by barrier and vessel type with two-photon imaging, mass-spec signatures and mouse/human data. They reveal arteriolar susceptibility, spatially matched astrocyte activation and S1pr1 stimulation as potential therapy.

## Introduction

In the human brain, surface (pial) vessels form the blood-cerebrospinal fluid barrier (BCSFB), whereas cerebral (parenchymal) vessels, primarily capillaries, constitute the blood-brain barrier (BBB). Capillaries directly contact brain tissue^1,2^, whereas intermediate segments, penetrating arterioles and ascending venules, exhibit features of both BCSFB and BBB, at least transcriptionally^3–6^. In pathology, dysfunction of the BCSFB/BBB is often described as a global disruption of junctional complexes. This view oversimplifies the fact that distinct vessel types are both structurally heterogeneous and serve specialized transport roles^7–10^. For example, non-specific vesicular transport of proteins, adsorptive-mediated transcytosis (AMT), is heavily suppressed in capillaries but moderately active in arterioles^9,11^, while immune cell trafficking and receptor-mediated transcytosis (RMT) of macromolecules occur primarily in venules^2,8^. Thus, changes in barrier permeability may occur via different mechanisms in different vessel types, a critical but underexplored nuance, with clear implications for diagnostic accuracy and therapeutic targeting in human diseases^12–14^.

Huntington’s Disease (HD) is a fatal neurodegenerative disorder with an urgent need for disease-modifying interventions. HD manifests with a toxic accumulation of mutant huntingtin (mHtt)^15^ that triggers progressive cellular dysfunction and brain atrophy^16–19^. The mHtt is expressed throughout the microvascular network, including arterioles, capillaries, and venules, where it aggregates in brain endothelial cells (BECs), the principal component of the BCSFB/BBB, as well as in pericytes, astrocytes, and smooth muscle cells^20–24^. These abnormalities manifest early, preceding the onset of motor, cognitive, and metabolic symptoms in HD^25,26^, making them a potentially valuable target for early intervention.

Here, we address these issues by characterizing barrier- and vessel-type-specific pathologies in HD. Using in vivo two-photon microscopy (2PM) and proteomics in R6/2 transgenic HD model mice^27^ combined with mice and human post-mortem tissue analyses, we provide a detailed account of BCSFB/BBB dysfunction, offering the first insights into vessel-type-specific alterations in paracellular leakage and vesicular transport in HD. We demonstrate that barrier dysfunction follows a distinct pattern: BCSFB paracellular leakage is confined to arterioles and venules belonging to large subpial vessels, while the paracellular barrier function is preserved at the level of BBB, i.e., capillaries. In comparison, vesicular transport by AMT increases up to 8-fold in parenchymal arterioles with only a modest 2.2-fold increase in brain capillaries. Treatment with Sphingosine-1-phosphate receptor 1 (S1pr1) agonist rescued BCSFB integrity and reduced AMT in arterioles, but did not restore BBB function in capillaries and venules. Astrocyte activation matched the topology of barrier breakdown, both in mice and HD patients’ brains, clustering around barrier leakage hot-spots, with more pronounced effects in female cases. These findings suggest that brain barrier defects in HD patients are treatable, holding promise for the development of more effective therapeutics for HD.

## Results

### BCSFB drives paracellular leakage in R6/2 mice

**Fig. 1:**
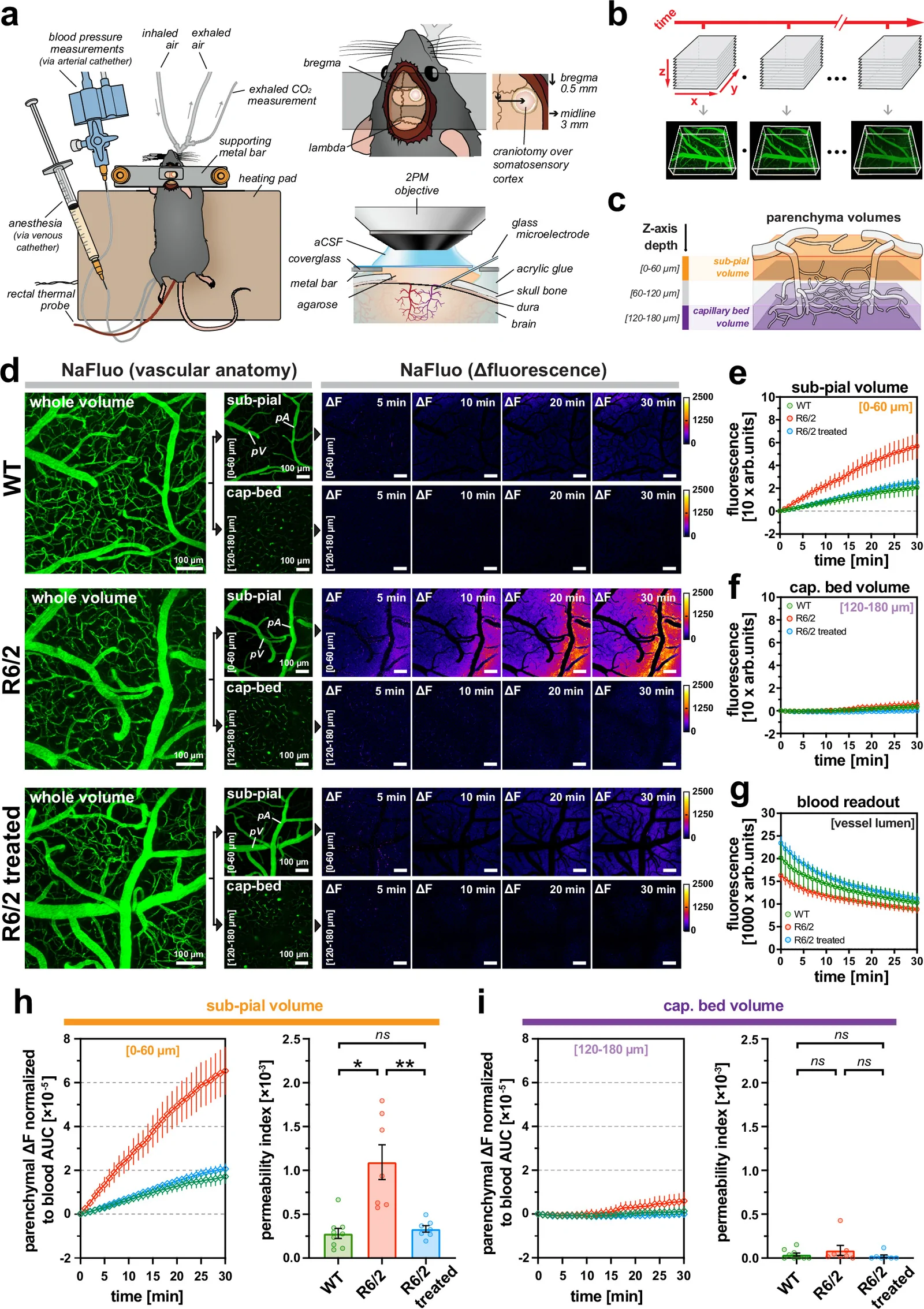
Pial vessels drive paracellular leakage pathology in R6/2 HD mice. **a** Mouse microsurgery preparation for in vivo two-photon imaging (2PM). **b** Principle of hyperstack imaging. Fluorescence was recorded as *z-*stacks over time, generating four-dimensional volumetric data (*xyzt*). The microvasculature is delineated by blood-circulating NaFluo. **c** Schematic of the analyzed cortical parenchyma. The imaged brain volume was divided into a sub-pial volume (0–60 µm below the pia), occupied predominantly by large pial vessels and sinuses forming the BCSFB, and a capillary bed volume (120–180 µm below the pia), occupied predominantly by capillaries forming the BBB. **d** Left panels: maximum-intensity projected *z-*stacks showing the anatomical features of the imaged cortical volume. Vessels are delineated by i.v.-injected NaFluo. Right panels: average-intensity projected *z-*stacks from the sub-pial and capillary-bed compartments (NaFluo in green), with corresponding time-lapse data showing gradual accumulation of NaFluo fluorescence in the brain parenchyma (ΔF arbitrary units) relative to baseline (*t* = 0 min post-injection, pseudocolor scale: Fire, ImageJ). R6/2 mice show increased paracellular leakage selectively in the sub-pial compartment. Treatment of R6/2 mice with the S1pr1 agonist SEW2871 reverses the leakage across the BCSFB. Average traces of NaFluo leakage to brain parenchyma in sub-pial (**e**) and capillary bed (**f**) volume. **g** Average traces of NaFluo decrease in the bloodstream. **h**, **i** Quantification of the NaFluo permeability index. The traces show the parenchymal NaFluo fluorescence increase from the sub-pial (**h**) and capillary-bed (**i**) volumes normalized to the AUC of the complete blood NaFluo fluorescence signal in (**g**). The bar graphs show the AUC of the normalized traces, representing the NaFluo permeability index. **All panels**: *n*_WT_ = 9; *n*_R6/2_ = 7; *n*_R6/2+_ = 7 mice; data is shown as average ± SEM; two-sided t-test; *=*p* < 0.05; **=*p* < 0.01; ns non-significant, aCSF artificial cerebrospinal fluid, NaFluo Sodium Fluorescein, LUT look-up table, arb.units. arbitrary fluorescence units (detector counts), cap-bed capillary bed volume, AUC area under curve. Exact group means, tests parameters and *p* values are provided in Supplementary Data 1, source data are provided in a Source Data file.

Vascular barrier dysfunction manifests as increased paracellular permeability between adjoining BECs, allowing blood-borne substances to invade the brain parenchyma. To spatially map this leakage in vivo, we performed 2PM imaging (Fig. 1a), using 23–24-week R6/2 mice, representing the late, severe disease stage (see model choice rationale in Supplementary Notes). We intravenously injected Sodium Fluorescein (NaFluo, 0.376 kDa, 1%, 40 µL per 20 g animal), a sensitive hydrophilic fluorophore for 2PM barrier permeability assays^11,28,29^. Immediately after, we collected hyperstack (*z-*stack over time) data from anatomically distinct cortical volumes^30^: the sub-pial volume (0–60 µm) primarily containing large BCSFB vessels, and the capillary bed volume (120–180 µm) with vessels containing the BBB (Fig. 1b, c). The data was collected simultaneously from all volumes.

Compared to wild-type (WT), R6/2 mice exhibited increased paracellular leakage at the sub-pial volume, indicated by substantial NaFluo accumulation, suggesting BCSFB dysfunction (Fig. 1d, e and Supplementary Video 1). In contrast, paracellular barrier function at the capillary bed was unchanged (Fig. 1d, f and Supplementary Video 1). To calculate permeability, we extracted NaFluo signals from blood vessels over time (Fig. 1g and Supplementary Fig. 1) and calculated the permeability index as the AUC of the parenchymal fluorescence increase normalized to the complete blood-signal AUC (Fig. 1h, i)^29^. Our results showed a ~ 3.8-fold increase in sub-pial paracellular permeability in R6/2 mice, with no significant change at the capillary BBB (Fig. 1h, i and Supplementary Data 1a, b).

Thus, the BCSFB-containing vessels in subpial regions, not BBB-containing capillaries in parenchyma, drive the pathological increase in paracellular permeability.

### Stimulating S1pr1 restores normal permeability

Sphingosine-1-phosphate (S1P) is a blood-borne signaling molecule activating S1P receptor 1 (S1pr1) at the luminal side of BECs^31^. Ongoing S1pr1 activity maintains low paracellular permeability of BECs, making S1pr1 signaling a targetable pathway in CNS, as evidenced in mice with BBB deficits^11,31,32^, and with the clinical success of the S1pr1 agonist fingolimod in treating ALS^33^. Therefore, we next tested whether S1pr1 agonism reverses BCSFB pathology in HD mice. Mice received intraperitoneal injections of SEW2871 (10 μg/g _BW_) for six days at 24-h intervals, with the final dose on the imaging day (see Methods). The treatment restored the BCSFB permeability in R6/2 to normal, low levels of WT mice (Fig. 1d–f, h and Supplementary Data 1c, d).

Thus, impairment of the S1pr1 pathway contributes to HD endothelial dysfunction in the R6/2 mice, which can be mitigated by S1pr1 agonism.

### Arterioles are most susceptible to AMT increase

**Fig. 2:**
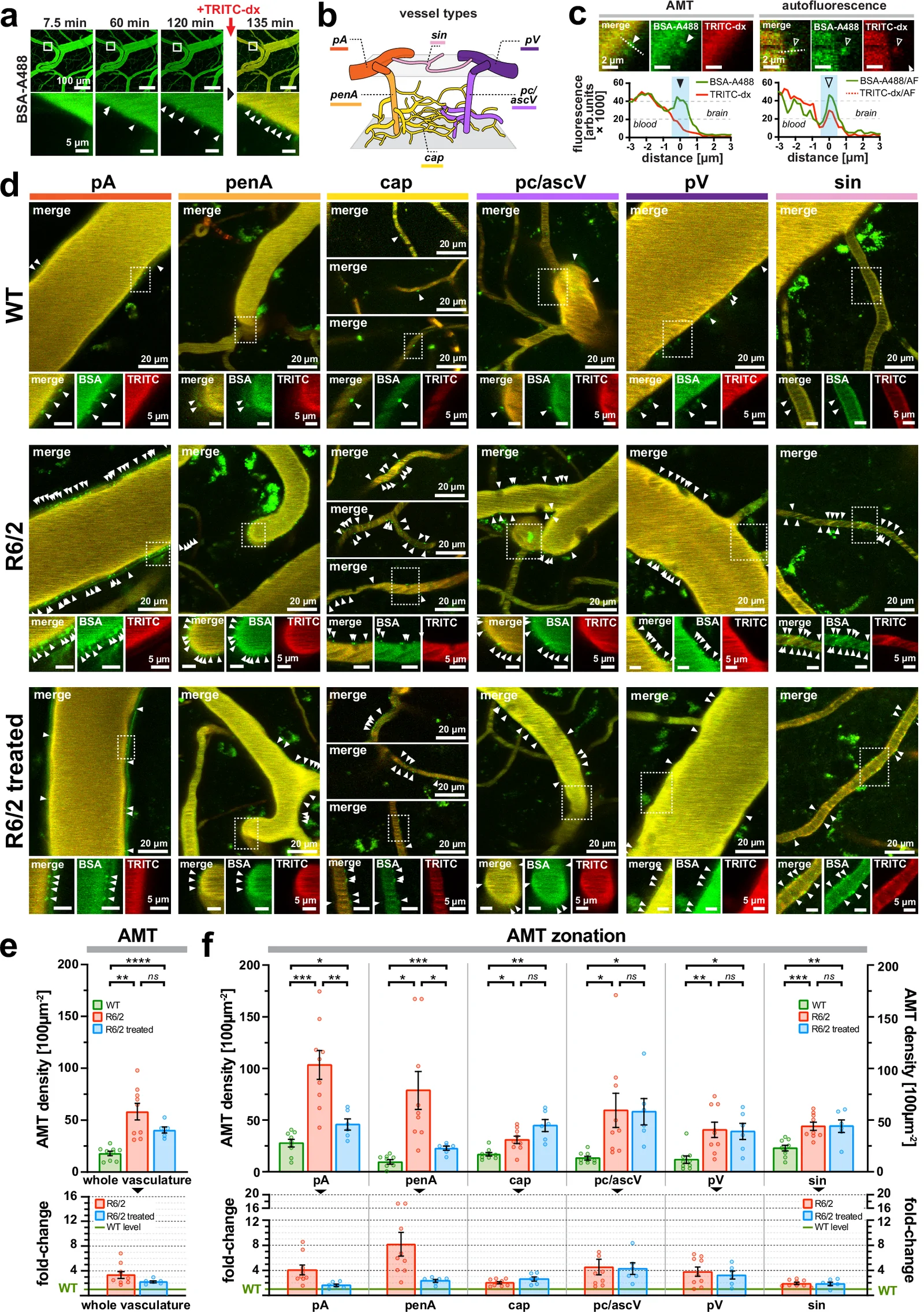
Vascular zonation of AMT pathology, and heterogeneous rescue potential of BCSFB and BBB in R6/2 HD mice. **a** Gradual uptake of blood-circulating BSA-A488 into discrete AMT puncta at the BCSFB/BBB interface; arrowheads in the insets mark individual puncta. TRITC-dx was injected after 120 min to delineate the vessel lumen. **b** Anatomical representation of the analyzed vessel types: pial arterioles (*pA*), penetrating arterioles (*penA)*, capillaries *(cap)*; post-capillary/ascending venules *(pc/ascV)*, pial venules (*pV*), and sinuses *(sin)*. **c** Distinction between AMT vesicular puncta and non-specific brain autofluorescence. AMT puncta (left) show signal only from BSA-A488 (green), whereas autofluorescent punctum also contain non-specific signal in the red emission range (red). The dashed line indicates the axis used for the fluorescence intensity profiles shown below. The filled arrowhead marks an AMT punctum, whereas the open arrowhead marks an autofluorescent punctum not classified as AMT. **d** Detailed images showing AMT puncta (arrowheads) at distinct vessel types, outlined in (**b**). The images were collected 135 min. post-BSA-A488 injection, with TRITC-dx delineating the vessel lumen. WT animals show a low degree of AMT across all vessel types, in contrast to R6/2 mice exhibiting overt pathological disinhibition of AMT, with the limited effect of the S1pr1-targeted treatment in pial and penetrating arterioles. **e**, **f** Upper panels show absolute AMT counts and lower panels show AMT levels as fold change relative to the WT baseline ( = 1). **e** Quantitative analyses of AMT puncta density (amount of puncta/vessel surface) showing the general increase in AMT in R6/2 mice and the overall effect of the S1pr1-targeted treatment in cortical vasculature. **f** Vessel-type-resolved analysis of AMT reveals the zonation vascular susceptibility to AMT increase in R6/2 mice and heterogeneous response to the treatment. The most sensitive are penetrating arterioles (*penA)*, the least sensitive are sinuses *(sin)* and capillaries *(cap)*. **All panels**: *n*_WT_ = 9; *n*_R6/2_ = 9; *n*_R6/2+_ = 6 mice; data is shown as average ± SEM; two-sided t-test with Bonferroni correction; *=*p* < 0.05; **=*p* < 0.01; ***=*p* < 0.001; ****=*p *< 0.0001; ns non-significant. Exact group means, tests parameters and *p* values are provided in Supplementary Data 1, source data are provided in a Source Data file.

AMT transports plasma proteins, such as albumin and IgGs, across the BBB^10,34^. In healthy brains, AMT is homeostatically suppressed by blood-borne S1P, and downstream, by the fatty acid transporter Mfsd2a (Major Facilitator Superfamily Domain containing 2a) in BECs^11,35,36^. AMT disinhibition is an early BBB dysfunction marker preceding ultrastructural alterations and paracellular leaks, e.g., in aging^10^ and stroke^37^. Although an increased presence of vesicular formations was detected in post-mortem HD brains^20^, its link to AMT was unclear. Using 2PM, we mapped AMT in R6/2 mice across vessel types in vivo. Mice were injected with fluorescent bovine serum albumin (BSA-A488), and its uptake in BECs was tracked over time. Within 120 min post-injection, and across all genotypes, we observed, a gradual appearance of AMT vesicles filled with BSA-A488 endocytosed from the bloodstream (Fig. 2a and Supplementary Fig. 2a–c; Supplementary Video 2) in all vessel types (Fig. 2b). Notably, we also detected a gradual uptake of transcytosed albumin in abluminal cells (Supplementary Video 3), reminiscent of the perivascular macrophages that actively sequester transcytosed albumin, as identified in our earlier work^11^. The subsequent 70 kDa TRITC-dx injection outlined the vessel lumen, allowing for clear differentiation of AMT vesicles from autofluorescent debris (Fig. 2a, c). In WT mice, AMT was uniformly low across all vessel types. In contrast, R6/2 mice showed widespread AMT disinhibition, with an overall ~3-fold increase (Fig. 2d, e and Supplementary Data 1e). Specifically, penetrating arterioles (penA) were the most affected, exhibiting an ~8-fold AMT increase, whereas pial arterioles (pA), pial venules (pV), and post-capillary/ascending venules (pc/ascV) had a moderate ~4-fold increase, and capillaries (cap) and sinuses (sin) were least affected, with a relatively modest ~2-fold increase over WT controls (Fig. 2f and Supplementary Data 1f–k). Notably, this increase was not due to, e.g., blood-flow stalling, but reflected a true rise in AMT, as stalled capillaries with no blood flow lacked AMT puncta enrichment (Supplementary Fig. 2d).

Thus, pathologic AMT disinhibition occurs across all vessel types in HD, affecting both the BCSFB and BBB. Yet, the extent of dysfunction is highly heterogeneous, with the greatest vulnerability in penetrating arterioles and the lowest in capillaries, indicating vessel-specific susceptibility to AMT increase.

### S1pr1 agonism suppresses AMT in arterioles but not in other vessel types

Beyond regulating paracellular permeability, the S1P–S1pr1 signaling axis suppresses AMT in inflammatory-like conditions^11^. Therefore, we next investigated whether S1pr1 agonist treatment alleviates increased AMT in the HD mice. We used the same mice as in the paracellular permeability assay, i.e., treated over six days with SEW2871 (10 μg/g _BW_), for the subsequent analysis of AMT. Treated R6/2 mice (R6/2+ ) showed an overall reduction in AMT (Fig. 2d, e). However, the effect was restricted to pial and penetrating arterioles (*pA, penA*), where AMT levels decreased (Fig. 2f), from ~4 to ~1.7-fold in pial arterioles and from ~8-fold to 2.4-fold in penetrating arterioles compared to WT baseline (Fig. 2f). In contrast, the treatment was ineffective for remaining vessel types, i.e., capillaries, post-capillary/ascending venules, pial venules, and sinuses (Fig. 2f and Supplementary Data 1l–s).

Thus, the heterogeneous rise in AMT across cerebral microvessels exhibits important differences in rescue potential for different vessel types.

### An inverse relation between paracellular permeability and AMT

In stroke, BBB dysfunction is biphasic, where either AMT increases or paracellular leakage predominates at different stages^37^. Whether a similar pattern exists for chronic brain disorders, including HD, is unknown. To test this, we cross-correlated paracellular leakage and AMT levels across cortical volumes within the same R6/2 mice and found an inverse correlation between paracellular leakage and AMT (Supplementary Fig. 3a). This was evident in pial arterioles within the subpial volume, where animals with high paracellular leakage exhibited only a moderate AMT increase, and vice versa. In contrast, this reciprocal relation was absent in less affected vessels, i.e., pial venules and sinuses occupying the same niche and capillaries (Supplementary Fig. 3b–d).

Thus, in the most vulnerable vessels, arterioles, either AMT or paracellular permeability predominates, suggesting a dynamic interplay between these two distinct mechanisms of BBB dysfunction.

### Mechanisms of BCSFB/BBB pathology and its therapeutic reversal

To probe mechanisms, we performed immunohistochemistry (IHC) analyses of brain sections and mass spectrometry (MS) on plasma and CSF from WT, R6/2, and SEW2871-treated R6/2 mice (10 μg/g for six days; referred to as R6/2+ ).

**Fig. 3:**
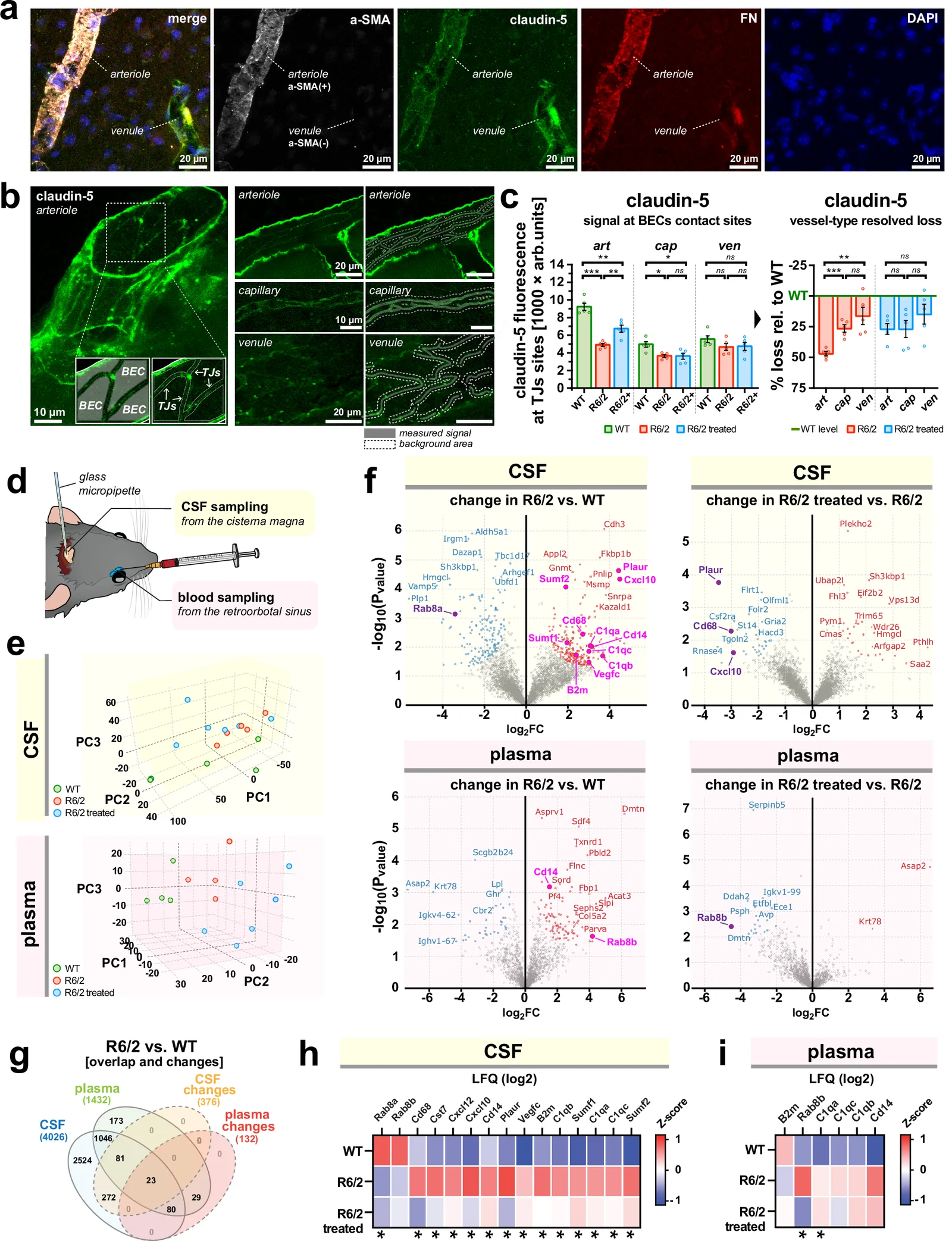
Molecular profiling of BCSFB/BBB dysfunction. **a** Cortical vessels immunostained for claudin-5, with α-SMA presence distinguishing arterioles from venules and fibronectin (FN) outlining vessel walls. **b** Left panel: the pattern of claudin-5 signal at brain endothelial cell (BEC) contact sites, at the expected location of tight junctions (TJs). Right panel: claudin-5 signal was quantified by subtracting the background signal adjacent to the TJs (dashed area) from the signal measured at the TJs (shaded gray area). **c** Left panel: claudin-5 fluorescence signal changes in arterioles (art), capillaries (cap), and venules (ven). Right panel shows that the relative reduction in R6/2 mice is greatest in arterioles. Treatment increases claudin-5 at TJs selectively in arterioles, but not capillaries and venules (R6/2+ mice). **d** Schematic drawing illustrating cerebrospinal fluid (CSF) and blood collection sites for mass spectrometry (MS) analysis. **e** Principal component analysis (PCA) of CSF and plasma proteomes, showing separation between WT and R6/2 mice and the effect of S1pr1 agonist treatment in both biofluids. **f** The volcano plots illustrate the significant protein expression changes between WT and R6/2 for both plasma and CSF, and the induced changes by treatment in both biofluids. Red and blue indicate increased and decreased protein abundance, respectively. Highlighted labels denote proteins of direct relevance to BCSFB/BBB function. **g** Venn diagram showing the overlap of identified proteins with significant changes across the two biofluids for the R6/2 vs WT. Changes in levels of relevant proteins for the BCSFB and BBB function in **h** CSF and **i** plasma. **IHC panels (3c)**: *n*_WT_ = 5; *n*_R6/2_ = 5; *n*_R6/2+_ = 5. Data is shown as average ± SEM; two-sided t-test with Bonferroni correction. *=*p* < 0.05; **=*p* < 0.01; ***=*p* < 0.001; *ns*=non-significant. **MS panels**: *n*_WT_ = 5; *n*_R6/2_ = 5; n_R6/2+_ = 6 mice. Differential abundance was assessed separately within each biofluid using two-sided independent-samples t-tests with permutation-based FDR control (FDR < 0.05, S0  =  0.3, 250 randomizations). The *P* values are exact permutation-based FDR-adjusted *P* values. *Log2* LFQ intensities were *z-*scored and significant changes shown with an asterisk(*). Exact group means, tests parameters and *p* values are provided in Supplementary Data 2 and 3, source data are provided in a Source Data file.

The cortical vessels from coronal sections were immunostained for claudin-5, alpha smooth muscle actin (α-SMA) to distinguish arterioles from venules, and fibronectin (FN) to outline the vessels, with DAPI to localize cell nuclei (Fig. 3a). Claudin-5 fluorescence signal was measured at the BECs in arterioles, capillaries and venules at cell contact sites, i.e., the locations of tight junctions relevant for maintaining low paracellular permeability (Fig. 3b). IHC revealed that R6/2 mice exhibited a loss of claudin-5 at tight junctions, especially in arterioles, showing nearly twice the reduction compared to capillaries and venules (Fig. 3c and Supplementary Data 2a–o). The treatment effect was also heterogeneous. S1pr1 agonism increased claudin-5 at TJs selectively in arterioles, with no effect in capillaries and venules (Fig. 3c and Supplementary Data 2c, f, i).

Next, we assessed CSF and blood plasma for MS proteomic fingerprinting (Fig. 3d). Proteomic quality control metrics: protein count, intensity, variability, and dynamic range, were consistent across conditions for both biofluids (Supplementary Fig. 4a–c). We identified 1638 proteins in plasma and 4746 in CSF. Unbiased principal component analysis (PCA) showed clear separation among WT, R6/2, and treated R6/2 groups in both biofluids (Fig. 3e). Comparing R6/2 to WT, we found 133 altered proteins in plasma and 382 in CSF, highlighting genotypic differences (Supplementary Data 3). Of these, three (Dctn3, Sod2, Tuba4a) in plasma and nine (Uqcr11, Atg2b, Ndufa8, Slc1a2, Ndufb6, Ndufb10, Tgm2, Psmd4, Sdhd) in CSF mapped to the KEGG HD pathway [mmu05016]. Treatment of R6/2 mice yielded 22 and 61 differential proteins in plasma and CSF, respectively (Fig. 3f), confirming treatment effects. Unsupervised clustering further distinguished all three groups, indicating distinct proteomic signatures across barriers (Supplementary Fig. 4d, e). Of 1230 shared proteins (86% overlap), 23 showed common significant changes (Fig. 3g). The proteomic profiles indicated heterogeneous molecular programs associated with barrier dysfunction (Tables 1 and 2). In R6/2 CSF, increased Cxcl12, Cd68, and C1q were consistent with inflammatory and complement-associated endothelial stress compatible with paracellular injury, whereas increased Rab8b, Sumf1/2, and Vegfc pointed to enhanced vesicle-trafficking and matrix-remodeling pathways associated with AMT dysregulation (Fig. 3h, i). S1pr1 agonist partially shifted this landscape toward normalization: Cxcl10 and Cd14 were reduced, indicating attenuation of barrier-associated inflammatory-stress signaling, while Rab8b and Plaur decreased, consistent with reduced vesicle trafficking and extracellular-matrix remodeling (Fig. 3h, i).

**Table 1:** Proteome signature of R6/2 mice

Protein	Identifier	Name	Source	Log2FC	P-value adjusted	Barrier Type	(E)xpression / vascular (T)arget	Plausible mechanism	PMID reference
Cxcl12 ↑	H7BX38	C-X-C motif chemokine 12	CSF	1.989	0.030	BCSFB	(E)Arteriole, (E)Capillaries, (E)Venules	[Paracellular ↑ ] Leukocyte infiltration at venules with perivascular space	PMID: 18276777
Cst7 ↑	O89098	Cystatin-7 (Cystatin-F)	CSF	3.139	0.050	BCSFB and BBB	(E)Arterioles	[Paracellular ↑ ] Cause of vascular injury via microglial remodeling of BBB	PMID: 3517880
Vegfc ↑	P97953	Vascular endothelial growth factor C	CSF	3.003	0.036	BCSFB	(E)Arterioles	[AMT ↑ ? + Paracellular ↑ ] VEGF family facilitates junction complexes remodeling	PMID: 24551038
C1q A↑ C1q B↑ C1q C ↑	P98086 P14106 Q02105	Complement C1q subcomponentsubunit A, B, C	CSF	3.137 3006 3.071	0.01830.0223 0.018	BCSFB and BBB	(T)Arterioles, (T)Capillaries	[Paracellular ↑ ] Complement-mediated endothelial disruption, deposition promotes leak	PMID: 30007547
Plaur ↑	A0A0U1RNN0	Plasminogen activator, urokinase receptor	CSF	4.432	0.001	BCSFB	(E)Arterioles, (E)Capillaries, (E)Venules	[Paracellular ↑ ] Involved in the degradation of the ECM, BBB permeability increase under pathological conditions.	PMID: 12427882
Rab8b ↑	P61028	Ras-related protein Rab-8B	Plasma	4.184	0.027	BCSFB and BBB	(E)Arterioles, (E)Capillaries, (E)Venules	[AMT ↑ ] Enhanced vesicular uptake	PMID: 34141705
Cd68 ↑	A0A0R4J1C8	CD68 antigen	CSF	2.713	0.014	BCSFB and BBB	(T)Arterioles, (T)Capillaries, (T)Venules	[Paracellular ↑ ] Phagocytic microglia-induced loss of BBB integrity	PMID: 31862977
B2m ↑	P01887	Beta-2-microglobulin	CSF	2.350	0.031	BCSFB and BBB	(E)Arterioles, (E)Capillaries, (E)Venules	[Paracellular ↑ ] Marker: B2M invades parenchyma when BBB is compromised	PMID:19008236
Sumf1 ↑	Q8R0F3	Sulfatase-modifying factor 1	CSF	2.082	0.024	BCSFB and BBB	(E)Arterioles, (E)Capillaries, (E)Venules	[AMT ↑ ? + Paracellular ↑ ] Regulates ECM. Altered sulfation of heparan sulfate may affect glycocalyx	PMID: 22298771
Sumf2 ↑	Q8BPG6	Sulfatase-modifying factor 2	CSF	1.904	0.007	BCSFB and BBB	(E)Arterioles, (E)Capillaries, (E)Venules	[AMT ↑ + Paracellular ↑ ] Lysosomal sulfatase, vesicle recycling involvement	PMID: 22298771
Rab8a ↓	P55258	Ras-related protein Rab-8A	CSF	-3.404	0.006	BCSFB and BBB	(E)Arterioles, (E)Capillaries, (E)Venules	[AMT ↓ ?] Impaired vesicle recycling	PMID: 24213529
Cxcl10 ↑	D3YW23	C-X-C motif chemokine 10	CSF	4.489	0.002	BCSFB	(T)Venules	[Paracellular ↑ ] Chemokine-mediated tight junction disruption, facilitates diapedesis at venules	PMID: 25339777
Cd14 ↑	P10810	Monocyte differentiation antigen CD14	CSF	3.675	0.024	BCSFB	(T)Venules	[Paracellular ↑ ] Monocyte infiltration, cytokine-induced permeability	PMID: 28732059
Cd14 ↑	P10810	Monocyte differentiation antigen Cd14	Plasma	1.512	0.008	BCSFB	(T)Venules	[Paracellular ↑ ] Monocyte activation, cytokine-induced permeability	PMID: 28732059

MS fingerprint of selected blood/CSF proteins significantly elevated or decreased in HD (R6/2 vs. WT) relevant for BCSFB/BBB function. The suggested affected vessel types are based on the preferential location of the protein transcriptome expression (marked E) in particular vessel types (PMID: 29443965), and/or mechanisms of action targeting particular vessel types (marked T, with PMID references). Differential abundance was assessed separately within each biofluid using two-sided independent-samples t-tests with permutation-based FDR control (FDR  <  0.05, S0  =  0.3, 250 randomizations). The *P* values are permutation-based FDR-adjusted *P* values. Source data are provided in Supplementary Data 3 and Source Data files.

*ECM* extracellular matrix, *TJ* tight junctions, *AMT* adsorptive-mediated transcytosis

**Table 2:** Proteome signature of S1pr1-agonist treated HD mice with implications for barrier restoration or further modulation

Protein	Identifier	Name	Source	Log2FC	P-value adjusted	Barrier Type	(E)xpression / vascular (T)arget	Plausible mechanism	PMID reference
Cd68 ↓	A0A0R4J1C8	Cd68 antigen	CSF	−3.016	0.012	BCSFB and BBB	(T)Arterioles, (T)Capillaries, (T)Venules	[Paracellular ↓ ] Inhibition of phagocytic microglia-induced loss of BBB integrity	PMID: 31862977
Plaur ↓	A0A0U1RNN0	Plasminogen activator, urokinase receptor	CSF	−3.478	0.002	BCSFB	(E)Arterioles, (E)Capillaries, (E)Venules	[Paracellular ↓ ] Facilitating ECM formation	PMID: 12427882
Rab8b ↓	P61028	Ras-related protein Rab-8B	Plasma	−4.511	0.025	BCSFB and BBB	(E)Arterioles, (E)Capillaries, (E)Venules	[AMT ↓ ] Decreased vesicular uptake	PMID: 34141705
Cxcl10 ↓	D3YW23	C-X-C motif chemokine 10	CSF	−2.927	0.029	BCSFB	(T)Venules	[Paracellular ↓ ] TJ stabilization	PMID: 25339777

MS fingerprint of selected blood/CSF proteins significantly elevated or decreased in S1PR1-agonist treated HD mice (R6/2+ vs. R6/2) relevant for BCSFB/BBB function. The suggested affected vessel types are based on the preferential location of the protein transcriptome expression (marked E) in particular vessel types (PMID: 29443965), and/or mechanisms of action targeting particular vessel types (marked T, with PMID references). Differential abundance was assessed separately within each biofluid using two-sided independent-samples t-tests with permutation-based FDR control (FDR  <  0.05, S0  =  0.3, 250 randomizations). The *P* values are permutation-based FDR-adjusted *P* values. Source data are provided in Supplementary Data 3 and Source Data files.

*ECM* extracellular matrix, *TJ* tight junctions, *AMT* adsorptive-mediated transcytosis.

Thus, although biofluid proteomics cannot assign these changes to specific vessel segments, it constrained plausible mechanisms of barrier dysfunction and identified molecular programs linked to the observed phenotypes: an inflammatory/paracellular-stress signature consistent with paracellular permeability changes, and a treatment-sensitive vesicle-trafficking signature that aligned with the arterioles pathology and selective rescue observed with 2PM in vivo and supported by claudin-5 IHC.

### Astrocyte activation mirrors patterns of BBB dysfunction

**Fig. 4:**
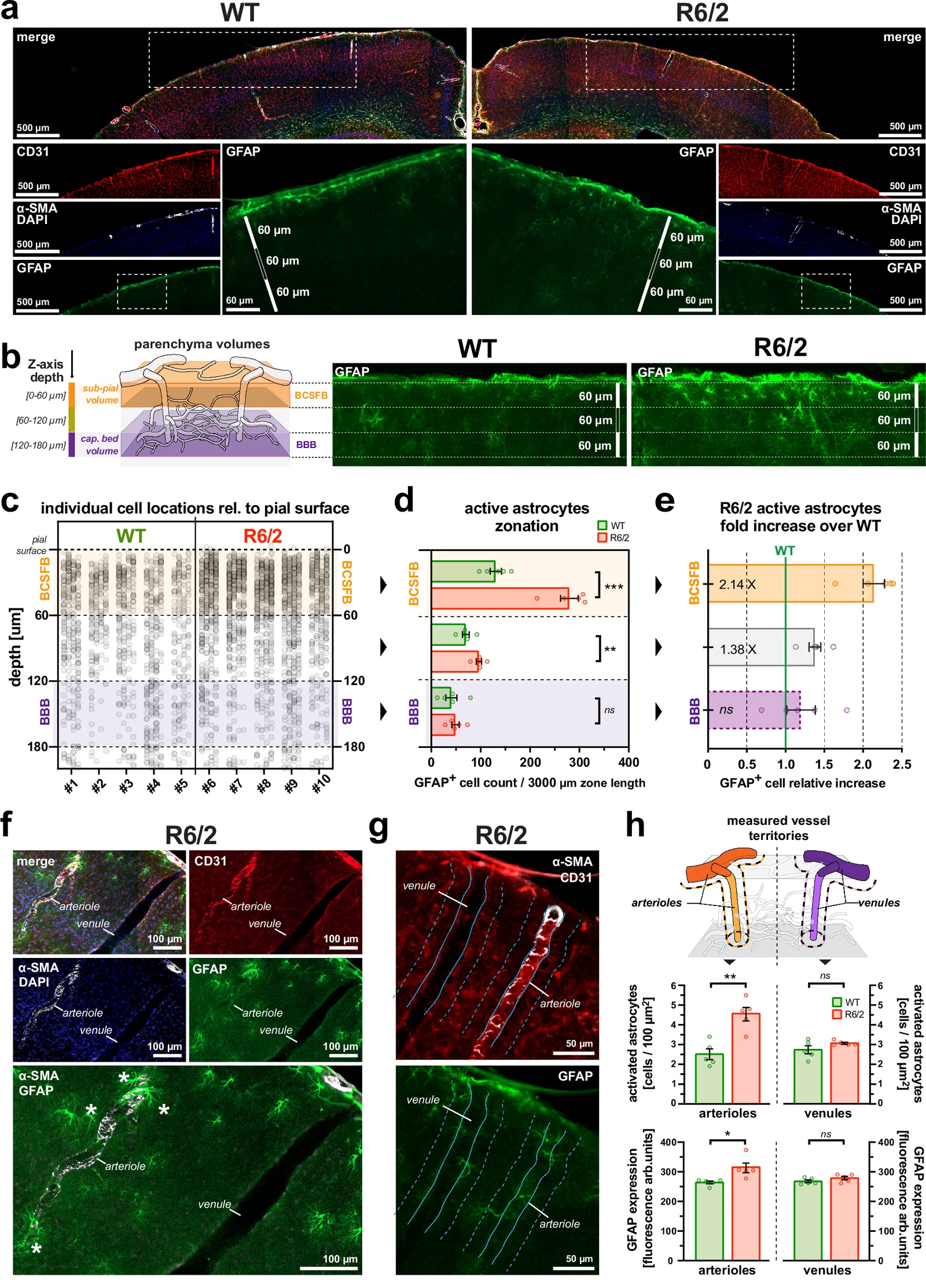
Astrocyte activation follows the topology of vascular barrier dysfunction in R6/2 HD mice. **a** Examples of immunostained sections showing cortical areas in WT and R6/2 mice. CD31 staining delineates vasculature (red), DAPI labels nuclei (blue), α-SMA labels arterioles (white), and GFAP stains activated astrocytes (green). **b** Left panel: Schematic of the analyzed cortical volumes: the sub-pial compartment (0–60 µm below the pia), enriched in pial vessels forming the BCSFB, and the capillary-bed compartment (120–180 µm), enriched in BBB capillaries. Right panel: corresponding staining images. **c** Distribution of GFAP+ astrocytes along a 3000 µm cortical segment. Each point represents an individual cell at its respective depth. Data from each mouse (marked with #) included four hemisphere profiles obtained from two coronal sections per mouse, arranged in columns. **d**, **e** GFAP+ astrocytes show a preferential increase in R6/2 mice in the superficial cortical area (0–60 µm, BCSFB influence), with no increase in numbers at the deeper layer (120–180 µm, capillary bed, BBB influence). **f** Example immunostaining images showing identification of arterioles by the presence of α-SMA signal, and accompanying GFAP+ astrocytes in proximity to vessels (asterisks). **g** Cut-off point distance (20 µm) of measured vessel territories for arterioles and venules. **h** Quantification of GFAP+ astrocyte density and their signal intensity in vessel territories, showing preferential enrichment in number and signal around arterioles, but not venules, in R6/2 mice. **All panels:** *n*_WT_ = 5; *n*_R6/2_ = 5 mice. Data is shown as average ± SEM; two-sided t-test; *=*p* < 0.05; **=*p* < 0.01; ***=*p* < 0.001; ns non-significant. Exact group means, tests parameters and *p* values are provided in Supplementary Data 2, source data are provided in a Source Data file.

We next asked whether this spatially and mechanistically heterogeneous barrier pathology is accompanied by equally localized responses in the adjacent parenchyma. Astrocytes react to BBB disruption by increasing glial fibrillary acidic protein (GFAP) expression, making them a reliable marker of barrier-related vascular abnormalities^38^. Using IHC, we visualized cerebral blood vessels (CD31), differentiated arterioles from venules (α-SMA presence), and assessed GFAP+ astrocyte activation in WT and R6/2 brain sections (Fig. 4a). GFAP+ astrocytes were increased in R6/2 mice, aligning with 2PM-detected paracellular leakage regions (Fig. 1). To quantify this response, we stratified cortical regions into 60 µm increments based on exposure to leakage from the BCSFB and BBB in 2PM experiments, enabling a direct comparison of astrocyte activation across these zones (Figs. 1, 4b and Supplementary Fig. 5).

Astrocyte activation followed a zonation, with GFAP+ astrocytes primarily enriched in cortical areas influenced by BCSFB leakage (0–60 µm)(Supplementary Data 2p–r). R6/2 mice exhibited a ~ 2.14-fold increase in the number of GFAP+ astrocytes compared to WT in these superficial cortical areas (Fig. 4c–e). In contrast, deeper regions associated with intact BBB function (120–180 µm, capillary bed) showed no significant increase in activated astrocyte numbers, suggesting that astrocyte responses are congruent with the affected barrier compartment (Fig. 4c–e). To further explore the spatial relationship between astrocyte activation and vascular dysfunction, we analyzed whether activated astrocytes were preferentially enriched near arterioles, which were most susceptible to AMT disinhibition. A standardized cut-off distance of 20 µm from the vessel wall was applied (Fig. 4f, g), approximately twice the average estimated distance between astrocyte and its nearest vessel in the brain^30^. We observed a twofold increase in astrocyte density around arterioles in R6/2 mice, which contrasted with no significant changes around venules (Fig. 4h and Supplementary Data 2s, t), reinforcing the link between astrocyte activation and most compromised vessel types, i.e., arterioles. Notably, we also detected elevated GFAP expression intensity, further confirming increased activation at sites of BBB dysfunction (Fig. 4h and Supplementary Data 2u, v). To rule out a generalized increase in glial cell density as an explanation, we used 2PM and intracortical SR101 injections to measure astrocyte density in vivo. While R6/2 mice showed a modest ~25% increase in total astrocyte density (Supplementary Fig. 6), this was insufficient to account for the twofold increase in GFAP+ astrocytes detected ex vivo, indicating that the observed enrichment of activated astrocytes is primarily a localized response to barrier dysfunction, rather than a generalized glial expansion throughout the cortex.

Thus, astrocyte activation is closely linked to the topography of barrier disruption, reinforcing the spatial coupling between the vessel-type-dependent segments of BCSFB/BBB dysfunction and glial reactivity in HD.

### Functional impact of chronic BBB leakage in HD

Astrocytes actively participate in synaptic transmission, neuronal modulation, and neurovascular responses. Their activation has been linked to cortical hyperexcitability that exacerbates neurodegeneration in chronic and acute disorders, both in humans and mice^39–41^. Given that increased paracellular leakage coincided with GFAP+ astrocyte activation in superficial cortical layers (~60 µm from the pia), we hypothesized that R6/2 mice may exhibit increased excitatory signaling and impaired inhibition, leading to cortical hyperexcitability, and if present, whether the paradigm of six-day treatment with SEW2871 (10 μg/g _BW_) that normalized sub-pial paracellular leakage and reduced AMT selectively in arterioles affects this phenotype.

**Fig. 5:**
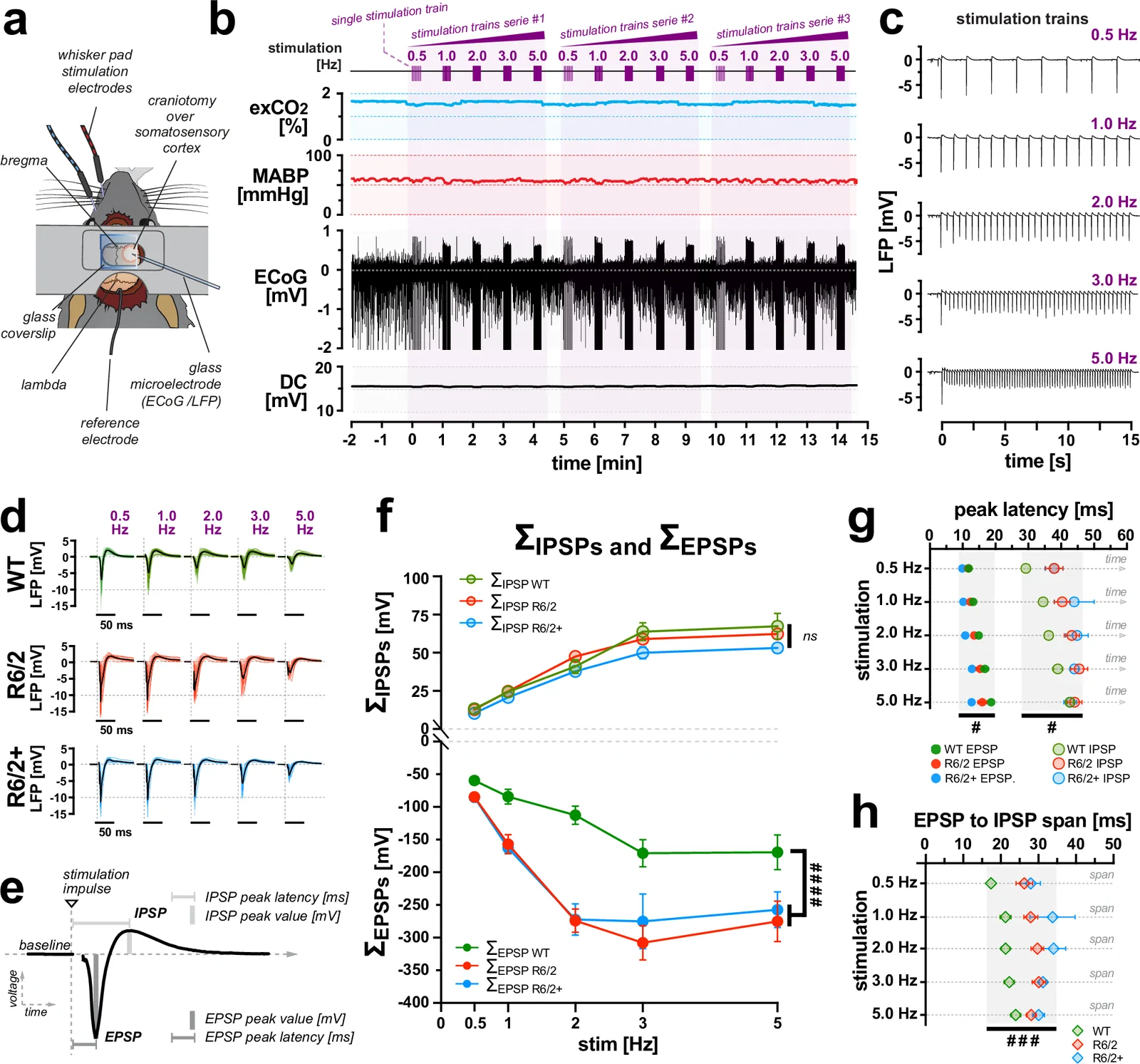
Apical barrel cortex hyperexcitability is an entrenched late-stage phenotype resisting barrier restoration. **a** The placement of electrodes for somatosensory stimulation of whisker barrel cortex. **b** Examples of concurrent recording of brain electrophysiological activity (electrocorticogram, *ECoG*), direct current *(DC)*, and accompanying systemic parameters, i.e., exhaled CO_2_ *(exCO*_*2*_*)* and mean arterial blood pressure *(MABP)*. The timeline shows the events of three series of 15 s stimulation trains ranging from 0.5 to 5.0 Hz. **c** Changes in local field potential *(LFP)* during stimulation trains. **d** The mean traces of LFP responses (black) for WT, R6/2, and R6/2 treated (R6/2+ ) mice, with tracers representing individual mice (colored). **e** The principal components of LFP response to somatosensory stimulation. Stimulation onset (*impulse)* triggers excitatory post-synaptic potential (*EPSP*), followed by compensating inhibitory post-synaptic potential (*IPSP)*. Both EPSP and IPSP were measured for peak (degree of potential change) and peak latency (peak time from stimulation onset). **f** Quantitative analyses of the total sum of EPSPs and IPSPs for each stimulation frequency in WT and R6/2 mice. Compared to WT, the R6/2 mice showed a significant increase in neuronal excitability (EPSPs) across stimulation frequencies. Treatment with SEW2871 did not normalize EPSPs and IPSPs in R6/2 mice (R6/2+ ). **g**, **h** Temporal profiles of synaptic activation showing that both R6/2 and R6/2+ mice have a quicker initiation of excitatory postsynaptic potentials (EPSPs) and a pronounced lag in inhibitory postsynaptic potentials (IPSPs) relative to WT, leading to an extended activation period (EPSP-to-IPSP span). **All panels:** *n*_WT_ = 11, *n*_R6/2_ = 10, *n*_R6/2+_ = 7 mice. Effects were evaluated with a two-way mixed-effects model (REML). The model tested group, frequency, and group × frequency effects. Šídák-adjusted post hoc comparisons were performed for detailed analysis. Hash signs denote the relevant omnibus effect from the REML ^#^=*p* < 0.05; ^# # #^ = *p* < 0.001; ^# # # #^ = *p* < 0.0001. Data is shown as average ± SEM. Exact group means, tests parameters and *p* values are provided in Supplementary Data 4, source data are provided in a Source Data file.

To test this, we conducted electrical stimulation of the whisker pad, activating the somatosensory (barrel) cortex, where thalamic inputs project to superficial cortical layers and the site of astrocyte activation^42^. Each mouse underwent three series of somatosensory stimulation across multiple frequencies while recording electrocorticogram (ECoG), local field potentials (LFPs), and direct current (DC) potentials from electrodes placed 50–70 µm into the barrel cortex, corresponding to the superficial cortical layer. We additionally monitored mean arterial blood pressure (MABP) and exhaled CO₂ (exCO₂) as systemic controls (Fig. 5a–c). For each stimulation train, we extracted excitatory post-synaptic potential (EPSP) peak values and latencies and inhibitory post-synaptic potential (IPSP) peak values and latencies at frequencies 0.5–5 Hz (Fig. 5d, e). To assess cumulative excitatory and inhibitory activity, we summed the EPSPs and IPSPs (ΣEPSPs, ΣIPSPs) over the full 15-s stimulation duration (Fig. 5f). We analyzed the data using two-way mixed-effects model (REML) with animal groups as the between-subject factor and stimulation frequency as the within-subject repeated factor, with repeated measurements matched by mouse (Supplementary Data 4). The results revealed that HD pathology leads to increased neuronal excitability, with a ~ 2-fold Σ_EPSPs_ increase across stimulation frequencies (Fig. 5f and Supplementary Data 4a, b). Moreover, R6/2 mice displayed altered activation and inhibition latencies - EPSPs occurred earlier, while IPSPs were delayed (Fig. 5g). This faster excitatory activation and delayed inhibition resulted in an extended activation period, with a longer EPSP-to-IPSP span in R6/2 than WT mice (Fig. 5h and Supplementary Data 4c–e). Of note, we observed instances of abnormal, ongoing spontaneous constrictions of pial arterioles in R6/2 mice, even without induced stimulation (Supplementary Video 4).

Next, we asked whether rapid restoration of vascular barrier function modifies the hyperexcitable state. Despite this robust vascular rescue, treated R6/2 mice remained electrophysiologically abnormal, together with earlier EPSP onset and delayed IPSP onset relative to WT (Fig. 5f–h).

Thus, short-term S1pr1 agonism dissociated two pathological levels: a rapidly reversible barrier phenotype and a late-stage cortical circuit phenotype that remained established at the time of analysis. This indicates that S1pr1 engagement retains clear therapeutic value in HD by restoring barrier integrity even at advanced disease stage, suggesting hyperexcitability as a downstream phenotype less amenable to acute correction.

### Human HD pathology patterns align with results in mice

**Fig. 6:**
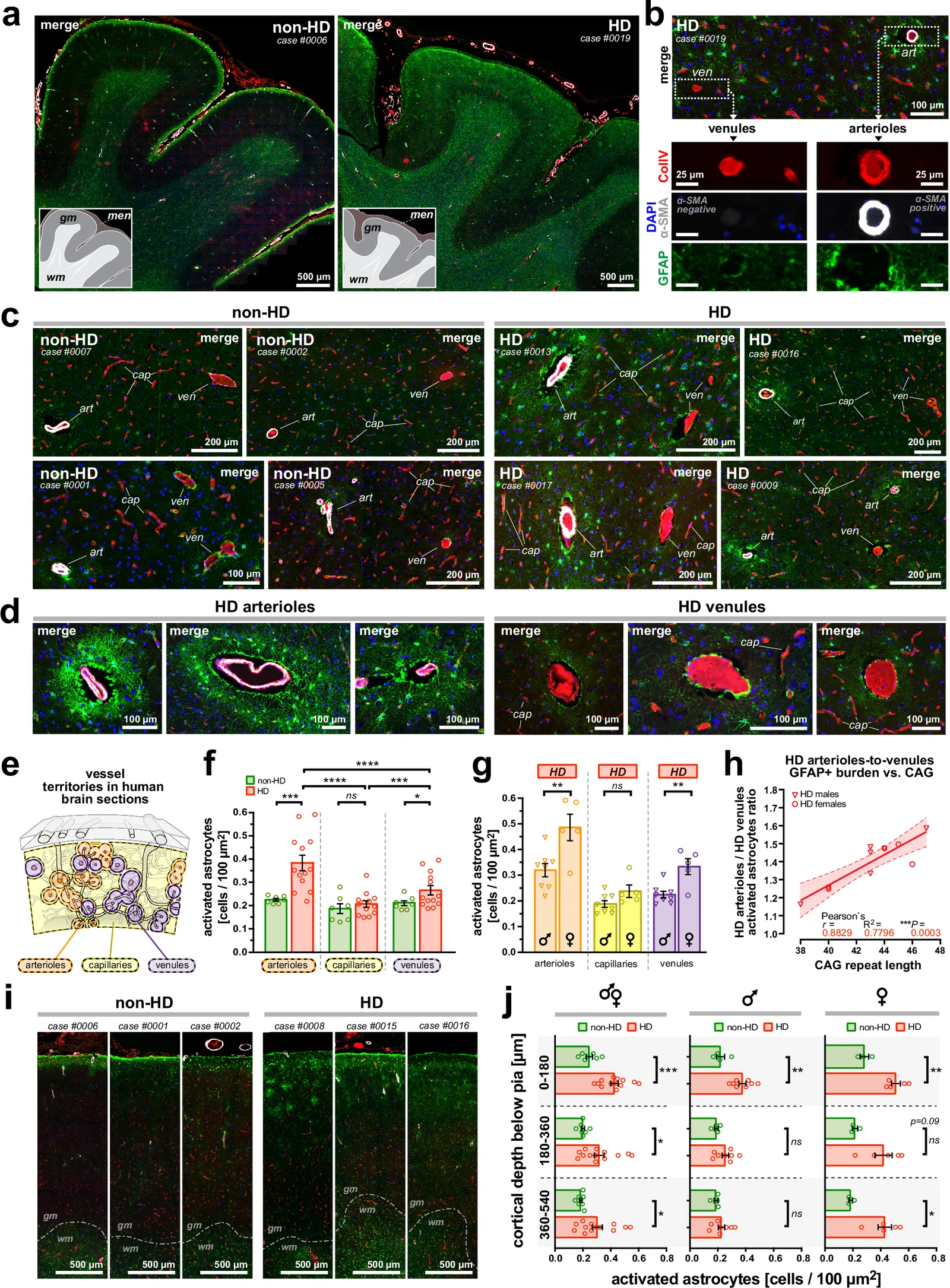
HD patients exhibit arteriolar susceptibility, mirroring the vascular pattern of astrocyte and barrier pathology observed in mice. **a**, **b** Immunostained cortical sections from non-HD and HD patients. Collagen IV marks vasculature, DAPI nuclei, α-SMA selectively identifies arterioles, and GFAP activated astrocytes (GFAP+ ). Insets highlight anatomical features. **c** Images from separate HD cases showing pronounced astrocyte activation near arterioles, with less affected venules, and the activation largely absent in non-HD brains. **d** The extent of astrocytic activation at arterioles in HD brains. **e** Schematic showing the location and orientation of analyzed vessel territories. **f** Mean densities of activated astrocytes in distinct vessel territories. HD is associated with marked periarteriolar enrichment, a moderate perivenular increase and no significant increase at capillaries. **g** Mean densities of activated astrocytes in male and female HD cases, with females being significantly more affected than males at both arterioles and venules with a trend towards increased activation in capillaries. **h** Pearson’s correlation analysis showing that asymmetry in astroglia activation between arterioles and venules, i.e., progressively greater periarteriolar activation relative to activation at venules, increases with CAG repeat length. *r* =Pearson’s correlation coefficient; *p*=two-sided probability under the null hypothesis of no linear correlation. The line and shading show the linear fit and 95% confidence interval. Each point represents a single HD case. **i** Images from individual HD cases showing enrichment of activated astrocytes at the superficial cortical layers. The dashed line marks the gray-white matter boundary. **j** Quantification of activated astrocytes across cortical depth. Astrocyte activation is most pronounced in the superficial cortical layers and decreases with depth. This effect is sex-dependent, with females showing increased activation across all analyzed depths and a more widespread and severe pathology. **All panels**: men meninges, gm gray matter, wm white matter, art arterioles, ven venules, cap capillaries. *n*_non-HD_ = 7 (4 males, 3 females); *n*_HD_ = 13 cases (8 males, 5 females). Data shown as average±SEM; *=*p* < 0.05; **=*p* < 0.01; ***=*p* < 0.001; ns non-significant. Exact patient data, group means, two-sided test types (Student’s t-test vs. Mann–Whitney) and parameters with *p* values are provided in Supplementary Data 5–7, source data are provided in a Source Data file.

Our findings in R6/2 mice revealed a striking vessel-dependent pattern of astrocyte activation, but a key question remained: does human HD pathology exhibit the same vessel- and region-specific heterogeneity? We analyzed post-mortem brain tissue from HD (*n* = 13) and non-HD patients (*n* = 7)(Supplementary Data 5). To avoid confounds such as meningeal damage or subarachnoid hemorrhage, we used cortical sections with well-preserved vessels and meninges. This was also to ensure comparable tissue preservation and to minimize sectioning artifacts at the cortical surface, which could compromise the results. This inclusion criterion was applied identically to HD and control cohorts. Given the relative paucity of prominent white matter in murine brains compared to humans, our human post-mortem analysis focused on gray matter for optimal R6/2 data comparability. Similarly to R6/2 mice, human HD brains exhibited vessel-specific astrocyte activation. Using Collagen IV to mark blood vessels, α-SMA for arterioles, and GFAP for astrocytes, we found that astrocyte activation was strongest around arterioles, forming dense halos of hypertrophic astrocytes (Fig. 6a, b). In contrast, venules and capillaries were associated with far less astrocyte activation (Fig. 6c, d and Supplementary Fig. 7a, b).

Although penetrating and ascending vessels appeared less frequent in humans than in mice-likely due to anatomical differences-the overall activation pattern resembled that seen in R6/2 mice (Supplementary Fig. 7c). To quantify this, we measured astrocyte density within a standardized 20μm radius from arterioles and venules forming the respective vessel territories, and considering the remaining territory as the territory of capillaries (Fig. 6e and Supplementary Fig. 8a, b)^30^. Our data shows that the most pronounced astrocyte activation occurred around arterioles (Fig. 6f and Supplementary Data 6a–f). In contrast, capillaries and venules lacked the prominent perivascular reactive astrogliosis defined by intense GFAP+ signal (Fig. 6f), consistent with prior reports of largely preserved cortical BBB-associated features despite vascular remodeling in HD^23,43^, and aligning closely with our 2PM AMT data in mice.

Interestingly, female HD patients exhibited ~40% greater astrocyte activation than males, for both arterioles and venules (Fig. 6g). This sex-driven effect was absent in non-HD brains, where astrocyte densities were comparable between males and females, regardless of vessel type (Supplementary Fig. 8c; Supplementary Data 6 g-l).

Notably, while CAG repeat length did not correlate with gross GFAP+ astrocyte density (Supplementary Fig. 8d), using correlation analysis, our data shows that the CAG length in patients may strongly predict arteriolar polarization in pathology, i.e., how much arterioles are affected compared to venules within each individual. Specifically, the periarteriolar-to-perivenular GFAP+ ratio (arteriolar GFAP+ density/venular GFAP+ density) increased with CAG repeat length (Fig. 6h and Supplementary Data 7). This may link the primary genetic determinant of HD to preferential arteriolar susceptibility rather than to a uniform increase in astrogliosis. By contrast, GFAP+ activation was unrelated to Vonsattel grade changes (Supplementary Fig. 8d), suggesting that this vessel-selective pattern may not simply be a consequence of neuropathological stage. Internal controls confirmed that age at death and post-mortem interval were not confounding factors in the observed GFAP+ changes (Supplementary Fig. 8e, f).

Next, we examined whether human astrocyte activation follows a gradient, as observed in BCSFB/BBB vulnerability in R6/2 mice, where superficial cortical layers exhibit greater pathology than deeper layers. Given that human cortical gray matter is ~3× thicker than in mice (2.5–3.0 mm vs. 1 mm)^44,45^, we adjusted our stratification from 60 μm increments in mice to three proportional layers in humans (0–180 μm, 180–360 μm, 360–540 μm)(Supplementary Fig. 8g). Our results revealed that, as observed in R6/2 mice, astrocyte activation in human HD brains was strongest in superficial cortical layers of gray matter and diminished with depth (Fig. 6i, j and Supplementary Data 6m–o). Notably, this effect was also sex-dependent. In males, significant astrocyte activation was confined to the superficial 0–180 μm layer, whereas in females, activation extended across all analyzed depths, indicating more severe pathology (Fig. 6j and Supplementary Data 6p–u).

Together, this cross-species concordance supports our conclusion that HD exhibits a vessel-type-specific hierarchy of vascular vulnerability where arterioles drive astrocyte activation while venules remain relatively spared, with strong sex-specific vulnerabilities.

## Discussion

**Fig. 7:**
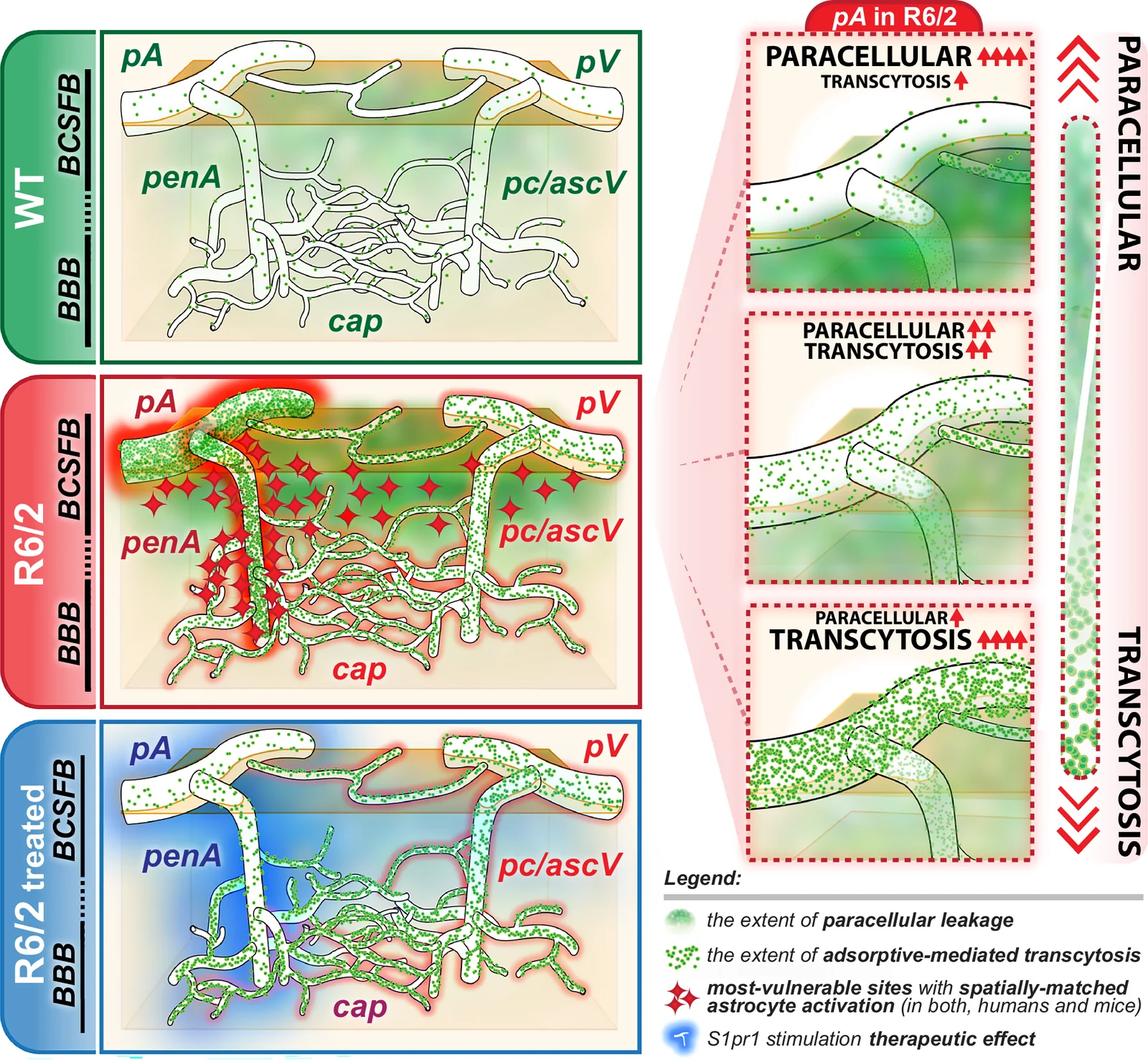
Working model of brain vascular dysfunction in HD. HD disrupts brain barriers in a vessel- and barrier-specific manner. Pial vessels drive paracellular leakage, while arterioles are the most susceptible vessels to pathological disinhibition in adsorptive-mediated transcytosis (AMT). This BCSFB and arteriolar susceptibility are spatially matched with astrocyte activation in humans and mice. S1pr1 agonism reverses paracellular leakage at pial vessels. Simultaneously, it reduces AMT, but only in arterioles, with only limited therapeutic effect on other vessel types. Right panels illustrate that either paracellular leakage or an AMT disinhibition is the dominant feature of vascular barrier dysfunction in pial arterioles in HD. BCSFB blood-CSF barrier, BBB blood-brain barrier, pA pial arterioles, penA penetrating arterioles; cap capillaries, pc/ascV post-capillary/ascending venules, pV pial venules, S1pr1 sphingosine-1-phosphate receptor 1, AMT adsorptive-mediated transcytosis.

Disruption of blood-brain barriers accompanies stroke^37^, epilepsy^46^, sepsis^47^, and chronic disorders such as Alzheimer’s and Parkinson’s disease (AD, PD)^13,14,48–51^. Recent work also implicates age-related microvascular alterations as a contributing factor to BBB dysfunction, reinforcing its role as both a target and a mediator of CNS pathology^10^. However, unlike other proteinopathies, BCSFB/BBB dysfunction in HD has received little attention. Seminal work shows BBB impairment in HD patients and R6/2 mice, implicating both paracellular defects and AMT alterations^20,43,52^. Yet the field remained split on how the barrier fails, paracellular versus transcytotic, or both, and whether dysfunction is diffuse or vessel-segment specific, a confusion amplified by bulk tracers that lack spatial resolution and which can generate false positives^53^. Here, we provide a high-resolution, pathway-specific, vessel-, and barrier-type (BCSFB/BBB) -resolved map that disentangles paracellular leakage from AMT disinhibition, establishing a compartmentalized, mechanism-specific topology of vascular dysfunction, integrating and explaining prior, seemingly divergent, observations and reveal a previously unknown regional variability across the cerebrovascular tree in HD (summarized in Fig. 7).

Paracellular permeability increase is the most recognized mechanism of barrier dysfunction. Despite widespread mHTT expression across all vessel segments and barrier types^20^, our functional 2PM assay revealed that increased paracellular leakage occurred selectively at the subpial BCSFB of R6/2 mice, whereas the capillary BBB function was preserved. At the structural level R6/2 mice showed reduced levels of claudin-5 at tight junctions, with the greatest loss in arterioles, relatively twofold more than in capillaries and venules. Although on structural level all vascular segments were affected, the central question is not merely whether TJ proteins are present, but whether the barrier is functionally competent. Indeed, the relationship between TJ protein levels and functional permeability is not always linear. A vessel can exhibit normal levels for claudin-5 ex vivo yet be functionally compromised in vivo due to e.g., mislocalization from the junctional strand (rapid claudin-5 disassembly/redistribution at endothelial junctions after permeability-inducing stimuli)^54^, or dynamic regulation (stimulus-dependent internalization and recycling of claudin-5 with concurrent barrier opening/closure)^55^, that may not be captured by ex vivo IHC assays. Conversely, a modest reduction in protein expression does not necessarily translate into a functionally significant leak, e.g., size-selective BBB loosening can occur without large-molecule leakage in claudin-5 deficiency^56^; and tissue/bed-dependentbasal permeability can be preserved despite substantial claudin-5 loss^57^. Indeed, increased permeability without the presence of ultrastructural TJ alterations were evidenced in iPSC models of juvenile HD^58^, and other CNS pathologies^59^.

In contrast to paracellular leak, AMT alterations were only recently recognized as indicative of barrier pathology that can facilitate brain dysfunction^10,34,37,59,60^, and the implications of vessel type-specific variations in AMT levels are still poorly understood^9–11^. It is plausible that mHtt has a direct effect on AMT because huntingtin is an essential integrator of intracellular vesicular trafficking, and mHtt can disrupt Rab-dependent pathways^61–63^. Here, our data suggest the S1PR1 and MFSD2A signaling axis as another central candidate. MFSD2A (Major Facilitator Superfamily Domain containing 2a) is a membrane transporter uniquely expressed by BECs, serving as a key regulator of BCSFB/BBB permeability by suppressing AMT^35^. Mfsd2a KO mice develop a leaky barrier from embryo through adult due to a dramatic increase in AMT despite intact TJs, supporting a vesicle-dominant leak phenotype rather than TJs collapse^35,36^. Recent mechanistic findings directly link Mfsd2a to S1P signaling at the BBB. S1P in the perivascular space binds endothelial S1PR1 receptors to strengthen barrier function, with Mfsd2a being essential for exporting S1P from brain endothelial cells, acting in complex with the S1P transporter^64^. This maintains an S1P rich microenvironment around the vasculature, necessary for formation and maintenance of the BBB^64^, whereas in Mfsd2a deficient mice, S1P levels in the endothelial basement membrane and extracellular matrix are greatly reduced, leading to barrier breakdown^64^. Consistently, deficits in S1P delivery mimic effects of Mfsd2a loss: ApoM, the plasma carrier of S1P, is required to present S1P to BBB endothelium and ApoM-/- mice exhibit increased permeability due to elevated AMT with no change in TJs^11^, very much like Mfsd2a-deficient mice. Notably, similar to R6/2 mice, the barrier deficits in ApoM-/- mice can also be reversed by administering an S1P1 agonist, confirming that the phenotype results from S1P signaling deficiency^11^. In addition, similarly to R6/2, ApoM-/- mice arterioles exhibited the highest degree of AMT disinhibition^11^. This arteriolar-specific susceptibility in both Apom-/- and HD mice is further supported by vascular zonation along cerebral vessels, i.e., S1PR1 expression is highest in arterioles along microvascular axis^3^, and arteriolar endothelium is specialized for caveolae-dependent processes, amplifying the consequences of AMT disinhibition and providing a basis for preferential arteriolar vulnerability^9,11^.

Notably, there is also emerging disease relevance in clinical HD. Single-cell dissection of human brain vasculature identified that immune activation co-occurs with concomitant downregulation of MFSD2A alongside other proteins critical for maintenance of BCSFB/BBB integrity in brains of HD patients^6^.

Here, we used a “slowed-down” R6/2 phenotype that provides a stable and sufficiently wide experimental window (23–24 weeks) to perform complex, low-throughput 2PM (see Supplementary Notes). In contrast, alternative models either lack substantial BBB disruption (YAC128, BACHD, KI) or require very long durations and additional insult to manifest any BBB phenotype (see Supplementary Notes). Intriguingly, in our model, the most affected arterioles tended to engage one route of barrier failure at a time, i.e., vessels with high paracellular leakage showed only modest vesicular uptake, and vice versa. This mutual exclusivity may reflect underlying vessel-specific regulation and possibly sequential dynamics. Acute stroke studies have shown that BBB disruption unfolds in phases–an initial surge in transcytosis followed by later tight-junction opening, rather than both mechanisms acting concurrently^37^. By analogy, in chronic HD, arterioles may be at different stages or under distinct stimuli, some manifesting early AMT-driven dysfunction while others progress to junctional breakdown, yielding the observed inverse correlation. Our focus on AMT and paracellular permeability and not receptor-mediated transcytosis (RMT) was a deliberate and pragmatic choice. Unlike AMT, the RMT lacks a broadly applicable in vivo tracer, as it relies on multiple receptor specific ligand systems with distinct kinetics and biodistribution^8,65–67^. Studying RMT comprehensively would provide a more holistic view on BCSFB/BBB dysfunction in pathology, and important future direction for HD, and other brain disorders.

Earlier studies reported the protective effects of S1pr1 agonists during inflammatory conditions^11,68^, including HD^69^, with fingolimod being a clinically approved S1pr1 modulator to treat ALS^33^. We observed that selective S1pr1 agonism significantly reduced barriers dysfunction, albeit the degree of rescue effect depended on the vessel type. The treatment was most effective in the most affected vessel types, both for paracellular leakage (pial vessels), and for disinhibition of AMT (arterioles). This response may be linked to the preferential expression of S1pr1 in arterioles^3^, and much stronger reliance on S1pr1 signaling for maintenance of the BBB in arterioles than in capillaries^11^. In turn, the lack of rescue at capillaries may be explained by a higher dependence on the Mfsd2a signaling pathway to regulate the AMT^35^. Nonetheless, these effects were achieved despite initiating treatment at a relatively advanced disease stage (approximately 23 weeks), with only a short 5-day intervention prior to imaging. In our study, we used SEW2871 as a short-term pharmacological probe rather than a candidate for chronic treatment. Our experiments involved continuous physiological monitoring during anesthesia and imaging, enabling detection of major systemic instability, and we observed no overt procedural exclusions attributable to the drug under our conditions. However, overt side effects were observed by others in other species, i.e., in rats^70^, suggesting that translation would require careful cardiovascular and immunological safety profiling, and prioritization of clinically optimized S1P pathway modulators. Still, reversing key barrier pathology aspects at this late-stage underscores S1pr1 agonists’ therapeutic potential for HD vascular dysfunction, warranting further attention as a promising intervention avenue even after disease progression.

Mass spectrometry cannot directly localize changes to vessel types but, instead, can provide an unbiased systems-level readout, constraining the plausible mechanisms underlying the three main observed phenotypes. Although not a causal proof, it revealed three molecular phenotypes. The first was complement/innate immune axis. CSF from R6/2 mice showed increased complement C1q subunits and microglial/macrophage markers (e.g., Cd68), alongside chemokines such as Cxcl12. Complement C1q is known to promote endothelial activation, vascular remodeling, and can aggravate brain injury and BBB dysfunction in vivo^71^. gC1qR signaling in brain microvascular endothelium also directly modulates permeability, as recently evidenced, e.g., in inflammatory responses post- stroke^72^. Together with elevated chemokines, these changes point to a causal contribution of venular immune cell recruitment and complement-mediated endothelial injury to the paracellular leakage we functionally localize to pial vessels. Second, vesicle trafficking and AMT machinery. We observed increased Rab8b and related vesicle-trafficking proteins and growth factors such as Vegfc, whereas Rab8a was decreased in R6/2 mice. Rab GTPases, including Rab8 family members, are core regulators of polarized membrane trafficking and recycling endosome function, which are central to transcytotic routes in endothelial cells^73,74^. Increased Rab8b is therefore mechanistically coherent with the pronounced AMT disinhibition we observed in penetrating arterioles, which were transcriptionally enriched for transport and vesicle-trafficking genes in independent datasets. Third, S1P/S1pr1-linked signatures. Our functional data show that S1pr1 agonism normalized BCSFB leakage and selectively suppressed AMT in arterioles. Proteomically, SEW2871 treatment partially reversed Rab8b and Plaur upregulation and reduced inflammatory markers such as Cxcl10 and Cd14, consistent with dampened vesicle trafficking and improved junctional integrity at the endothelium. This converges with prior work demonstrating that ApoM-bound S1P and pharmacological S1pr1 stimulation (including SEW2871) maintain low paracellular permeability across the BBB and suppress AMT in arterioles^11^ and, more generally, that S1pr1 signaling enhances endothelial barrier function^75^. These inferences were supported by the expression patterns of proteins such as Cxcl12, Cd68, C1q, Rab8b, and Vegfc, or their vascular targets^3,6^, suggesting a vessel-dependent susceptibility in the disease.

The observation that SEW2871 shifts protein modules that are functionally linked to S1P/S1pr1-dependent control of AMT and paracellular permeability strengthens the argument that these molecular changes are not generic “late-stage noise” but are mechanistically relevant to the barrier defects. Notably, CSF proteome in late-stage HD had at least two components. One comprised proteins that arise directly from BCSFB/BBB vascular deficits and are sensitive to S1PR1 agonism (e.g., proteins leaked from blood or released by acutely activated cells). The other likely reflected downstream pathologies, such as advanced neurodegeneration or chronic, self-perpetuating neuroinflammation, that are not reversible by short-term vascular repair. This supports our hypothesis of a therapeutic window, and although vascular integrity can be restored, reversing the full spectrum of pathological changes may require earlier or more prolonged intervention. However, the definitive causality ultimately requires targeted manipulations of specific candidates, e.g., endothelial-restricted modulation of Rab8b or pharmacological/complement-blocking interventions guided by our proteome signature and cell-type-resolved proteomics or transcriptomics^73,74^. In this scenario, our findings provide the field with a barrier-relevant proteomic landscape and a mechanistic framework with a prioritized list of molecular pathways for future causal interrogation, and may inform the development of targeted therapeutic strategies in HD, specifically those aimed at barriers and vessels.

Although S1PR1 is not endothelial-specific, the rescue pattern observed here is most consistent with a predominantly endothelial action of SEW2871. S1pr1 is abundant in endothelial cells, especially enriched in arterioles, with markedly lower expression in neurons and astrocytes, and minimal expression in microglia^6,76–79^. This distribution supports the point that a systemically accessible agonist would be expected to exert prominent effects at the endothelial barrier, particularly in arterioles. Astrocytes do express S1PR1, and astrocytic signaling can shape BBB function in disease contexts^78^. Neuronal S1PR1 can also modulate intrinsic properties and excitability in a circuit and cell type-dependent manner^80^. Systemically, S1pr1 agonism can also reduce inflammation by promoting anti-inflammatory macrophage phenotypes and restraining lymphopoiesis^81–83^, benefiting barriers strength. Here, the SEW2871 effect on claudin-5 expression and AMT was largely confined to arterioles with no detectable rescue in capillaries or venules. If SEW2871 was acting primarily via general immunosuppression, one might expect a more uniform and widespread rescue across all vessel types. In addition, our observation is supported by previous findings in ApoM KO mice, where endothelial S1PR1 signaling deficits produce a barrier phenotype dominated by increased AMT in arterioles^11^, closely mirroring the HD phenotype in our study. While we cannot exclude a secondary, beneficial effect on S1PR1 stimulation of other cell types, in the context of barriers rescue, the most plausible model appears to be direct action on compromised endothelium. Noteworthy, while reduced S1P levels has been observed in animal models, including R6/2 mice^84,85^, the transcript levels of S1PR1 appear to be unchanged in HD patients^6^, suggesting that rather than S1PR1 downregulation, reduced blood-borne S1P levels may play a role in impaired S1PR1 signaling.

The invasion of blood-derived factors into the brain microenvironment triggers astroglial activation, with albumin activating astrocytes via TGF-β signaling, triggering a senescent, pro-inflammatory phenotype^86^. Vascular and glial dysfunctions emerge as early contributors to HD pathogenesis, detected even in presymptomatic HD carriers (Grade 0 HD), where striatal astrocytes exhibit increased GFAP expression, indicative of early activation^87^. Here, astrocyte reactivity was increased locally at regions exhibiting BCSFB leakage. Although activated astrocytes may locally release neurotrophic factors and antioxidants, they can also acquire neurotoxic properties, secreting reactive oxygen species and excitotoxic metabolites such as quinolinic acid, driving cell damage^88^. This chronic astrogliosis may further compromise essential astrocytic functions involved in the regulation of neurotransmission^88,89^. The extravasated serum albumin binds to TGFβ receptors on astrocytes, triggering a signaling cascade (SMAD2 phosphorylation) that results in the downregulation of the potassium channel Kir4.1 and the glutamate transporter EAAT2^41^, resulting in the loss of buffering capacity and a persistent state of neuronal hyperexcitability. Consistent with this framework, R6/2 mice exhibited increased neuronal excitability, with earlier EPSP onset and delayed IPSP onset. Although these data do not establish direct causality between barrier dysfunction, astrocyte activation, and altered neurotransmission, there is strong mechanistic precedent that barrier failure can promote hyperexcitability through albumin-driven astrocytic TGFβ signaling, impaired potassium and glutamate homeostasis, and enhanced excitatory synaptogenesis^90–93^.

In patients, cortical hyperexcitability is a well-established feature of HD, detectable even at early disease stages^94^, and sensitivity to glutamatergic excitotoxicity increases with disease progression^95^, consistent with a multifactorial origin^96–98^. Our results show that while the six-day SEW2871 treatment robustly reversed the barrier pathology, it did not normalize cortical hyperexcitability at late disease stage. Because BCSFB/BBB abnormalities emerge early in both R6/2 mice and patients, these findings suggest a plausible early therapeutic window for vascular intervention^20^. Consistent with this, sustained, early modulation of the S1P axis in HD models may improve motor performance, extend survival, and preserve synaptic function^52,69,99^. In addition, barrier leakage can drive durable circuit remodeling by albumin-driven excitatory synaptogenesis. Blocking these downstream pathways may prevent later hyperexcitability, reinforcing the prevention versus reversal treatment strategies^93,100,101^.

While paracellular permeability and AMT are intrinsic to endothelial cells, BECs can be influenced, besides astrocytes, by other cells of NVU. For instance, dysfunctional pericytes could fail to provide the stabilizing signals to endothelial cells, contributing to the disinhibition of AMT^102,103^. Hypothetically, pathology in VSMCs could also lead to chronic alterations in blood flow and shear stress, which could secondarily compromise endothelial function^104^, e.g., via shedding of glycocalyx^105,106^. Consistent with this, we also observed spontaneous arteriolar constrictions in vivo. Because such events can occur on a seconds timescale, whereas our volumetric imaging was acquired every 7.5 min to prioritize high-resolution mapping across large cortical volumes, the present dataset supports qualitative identification of these dynamics but not robust kinetic analysis, highlighting rapid arteriolar constriction as an important target for future high-speed in vivo imaging.

Notably, the segments most enriched in VSMCs (arterioles) and pericytes (capillaries) are also those in which we observed the most distinct differences in susceptibility to pathology. This pattern is consistent with segment-specific mural cell contributions to BCSFB and BBB maintenance. Our multimodal dataset also provides several convergent lines supporting the involvement of recruited immune cells and microglia, which can drive the local BEC dysfunction and increased astrogliosis. First, our blood and CSF mass spectrometry signature in R6/2 mice is enriched for factors consistent with myeloid engagement and complement activity in CNS-facing compartments, including elevated Cxcl12, Cd68, and C1q in CSF, together with treatment-sensitive immune access markers such as Cd14 and Cxcl10. In the context of barrier compromise, CXCL12 is a key endothelial chemokine that regulates leukocyte positioning and entry at the blood brain barrier, and perturbations of the CXCL12 axis permit parenchymal leukocyte invasion in neuroinflammatory settings, providing a mechanistic bridge from barrier dysfunction to recruited immune cells^107–109^. Second, we observed that transcytosed albumin is taken up by perivascular macrophages (cell identity verified in our previous work^11^), demonstrating direct delivery of blood-borne cargo to immune-competent cells that can amplify local cytokine and complement cascades. Third, the partial normalization of these CSF inflammatory and vesicular markers with S1pr1 agonism is consistent with reduced immune access when barrier function improves, supporting directionality from barrier failure to immune activation. Lastly, mechanistically, activated microglia can induce reactive astrocyte programs via IL1α, TNF, and C1q, and albumin can further potentiate astrocyte reactivity through TGFβ receptor signaling, aligning well with the proposed sequence^91,92,110^. However, dedicated studies are still needed to determine how the spatial organization and heterogeneity of mural and immune cell populations, collectively or individually, regulate barrier in HD.

Our focus on the cortex was not only driven by practical considerations of 2PM. First, cortical atrophy and cortical thinning occur early and often in the preclinical stage of HD alongside or even beyond striatal changes, and their progression has been linked with the extent of the impairment of cognitive, memory and executive function^111^. This indicates that cortical pathology is not a late by-product of striatal loss, but at the core of HD neurodegeneration^112^. Moreover, cortical vascular dysfunction may also directly impact cortico-striatal circuits. For example, recent human data show that vascular remodeling in the HD cortex correlates with the extent of striatal degeneration and earlier symptom onset^43^, suggesting that cortical vascular deficits exacerbate network impairment in HD. While our late-stage experiments do not resolve temporal causality, i.e., whether vascular changes initiate pathology, several observations argue against barrier breakdown as purely end-stage, nonspecific consequence of neurodegeneration. Cerebrovascular and blood-brain barrier abnormalities can occur early in HD, including functional and ultrastructural barrier alterations in R6/2 mice and human tissue that precede neuronal loss^20^. Physiological imaging in individuals with HD also reports impaired cerebrovascular reactivity, consistent with an early neurovascular component^113^. More broadly, HD pathology evolves for years before diagnosable onset, leaving ample window for progressive neurovascular contributions^17^, with MRI assessment of perivascular spaces in premanifest HD suggesting impairment of vascular clearance pathways, even when global effects are subtle^114^. We propose that the relationship between vascular abnormalities and neurodegeneration is likely a vicious cycle. Early paracellular leakage at the BCSFB and increased AMT at the BBB allow chronic exposure of the brain parenchyma to neurotoxic and pro-inflammatory blood-derived components (e.g., albumin, complement factors, cytokines). This may create a toxic microenvironment that accelerates neuronal stress, synaptic dysfunction, and ultimately, neurodegeneration. In turn, degenerating neurons and glia can release factors that further compromise vascular integrity, perpetuating the cycle.

In summary, this study identified a zonation of vascular dysfunction in HD, with a promising therapeutic avenue. We propose moving beyond the binary concept of an intact versus “leaky” BBB in HD and instead present a model of compartmentalized, regional variability across the cerebrovascular tree. Our molecular, structural, and functional data, supported by proteomic fingerprinting, provide a proactive path forward for targeted interventions and personalized diagnostics. Further studies are warranted to expand on these findings and translate them into human therapeutics, both in HD and, by extension, other neurodegenerative disorders.

## Methods

### Ethical considerations

Formalin-fixed paraffin-embedded 5 µm-thick cortex sections from Huntington’s disease (HD) patients and age- and sex-matched controls were acquired through the University of Washington Biorepository and Integrated Neuropathology Lab (PI: C. Dirk Keene, MD, PhD) under the REC of the CHU de Québec ethical approval A13-02-1138. Informed consent for research brain donation was obtained from the legal next of kin according to protocols approved by the University of Washington Institutional Review Board that conform to the provisions of the Declaration of Helsinki and preserve patient anonymity. All animal surgical and imaging protocols were approved by the Danish Animal Experiments Inspectorate. The experiments followed ARRIVE guidelines and conformed to the standards established by the European Council Convention for the Protection of Vertebrate Animals Used for Experimental and Other Scientific Purposes (permit #2019-15-0201-01655).

### Animal model

We used 23-to 24-week-old heterozygous R6/2 mice, corresponding to late-stage disease, with their age-matched wild-type (WT) littermates as controls. The R6/2 colony exhibited onset of the disease at ~16 weeks of age (266 to 393 CAG repeats), resulting in slower disease progression compared to the original R6/2 line with shorter expansion, i.e., ~150 CAG repeats^115,116^. This enabled practical, day-to-day two-photon microscopy sessions with a maximum throughput of one imaged animal per day and an age difference of no more than two weeks between R6/2 groups and age-matched healthy littermates (B6CBA mice, referred to as WT)(see also Supplementary Notes on model choice broad rationale). The mice were genotyped for the human mutant huntingtin gene (*mHTT*), as previously described^27^. The animals were housed in climate-controlled ventilated cages (at 50 ± 10% relative humidity, at room temperature), with a 12 h light/ 12 h dark cycle, with a standard (constant, low) noise level. All mice had *ad libitum* access to a standardized natural-ingredient diet (SAFE D30) supplied in ~10 mm pellets and water. R6/2 mice were monitored throughout disease progression and were euthanized by 6 months of age to prevent severe suffering. Humane endpoints included >20% body-weight loss, inability to eat, drink or right themselves, severe neurological deficits, or marked deterioration in general condition. Animals were euthanized by pentobarbital overdose followed by cervical dislocation. The animal housing facility has been accredited by the Association for Assessment and Accreditation of Laboratory Animal Care (AAALAC), and the Federation of Laboratory Animal Science Associations (FELASA).

### Surgical preparations for two-photon imaging in vivo

Animals underwent microsurgical preparation, as described before, with minor modifications^8^. Briefly, animals were initially sedated via intraperitoneal (i.p.) injections of xylazine (10 μg/g _BW_) and ketamine (60 μg/g _BW_). Subsequently, and through all steps of surgery, the anesthesia was maintained with recurrent i.p. injections of ketamine (30 μg/g _BW_) administered at 20–25 min intervals. A tracheotomy was performed to facilitate mechanical respiration with a stroke volume of 180–220 μL at the frequency 190–240 strokes /min (MiniVent Type 845 respirator, Harvard Apparatus). The inhaled air was supplemented with oxygen at the rate of 1.5–2 mL/min. Next, dual catheterization was performed. The first catheter was inserted into the left femoral artery to administer substances and monitor the mean arterial blood pressure (MABP; BP-1 Pressure Monitor, World Precision Instruments). The second catheter was inserted into the femoral vein to sustain anesthesia during imaging. Post-catheterization, all wounds were surgically closed, and the animal was turned to the prone position for a craniotomy. The scalp was removed, and the periosteum was cleared with a cotton swab soaked in FeCl_3_ solution. Next, the cranium was attached to a custom metal head plate using glue (Loctite Adhesives). A craniotomy was performed above the right somatosensory cortex, at the coordinates of 3 mm laterally and 0.5 mm posterior to the bregma, with Ø = 4 mm, using a diamond dental drill at 4500 rpm. The bone flap was carefully elevated, the dura mater excised, and a low-melting point agarose (type III-A, Sigma-Aldrich) 1% solution in artificial cerebrospinal fluid (aCSF; in mM: NaCl 120; KCl 2.8; Na_2_HPO_4_ 1; MgCl_2_ 0.876; NaHCO_3_ 22; CaCl_2_ 1.45; glucose 2.55; pH = 7.4) was applied to the cerebral surface. Subsequently, a ~ 4 mm square and 0.08 mm thickness imaging coverslip (Menzel-Gläser, 24 × 66 mm, #1.5) was placed to seal the craniotomy, leaving a margin of ~0.5 mm for the insertion of glass microelectrodes into the cortex. Next, the animal was relocated to the two-photon microscope imaging stage, and anesthesia was switched to a continuous intravenous (i.v.) infusion with α-chloralose (50 mg/kg_BW_/h)(Figs. 1a, 5a)

The animal was allowed to rest for ~25 min. before imaging to enable the washout of ketamine from the system, which, unlike α-chloralose, hinders NMDA receptor-dependent excitatory neurotransmission^117,118^. To maintain the animal’s physiological state, during all surgical and imaging procedures, the end-tidal CO_2_ (Capnograph Type 340, Harvard Apparatus) and mean MABP were monitored in real-time, and normothermia at 37 °C was ensured via a rectal thermistor-regulated heating pad. Before imaging, a 50 μL blood sample was collected via arterial catheter to assess blood gases (ABL Radiometer), and the respiration volume and rate were adjusted if necessary.

### Drug treatment

The animals were acutely treated with i.p. injections of SEW2871, the sphingosine-1-phosphate (S1P) receptor (S1pr1) agonist. We chose SEW2871 because it is a high affinity S1pr1 selective agonist developed to recapitulate S1pr1-driven biology^119^, without the broader subtype engagement of e.g., fingolimod, which after phosphorylation acts on S1pr1, S1pr3, S1pr4, and S1pr5 and functionally antagonizes S1pr1 through internalization and downregulation^120,121^. Mechanistically, this mattered for our study because across cell types and receptor subtypes; a S1PR1 selective agonist SEW2871 provides a cleaner pharmacological probe than a multi subtype modulator.

The treatment started five days prior to imaging procedures, and SEW2871 was administered to 23–24-week-old mice, in 24-h intervals at the dose of 10 μg/g body weight (BW) in PBS with 10% DMSO. The WT and control R6/2 groups received the corresponding injections with DMSO solution only. The total number of injections was six per animal, with the final injection performed on the experimental day, three hours before the imaging procedures. We used this dose and a six-day administration paradigm to achieve robust receptor engagement, consistent with prior cerebrovascular disease models supporting daily intraperitoneal SEW2871 dosing as a practical schedule for sustained, time-controlled receptor stimulation^106,122,123^. The drug was prepared in PBS containing 10% DMSO, with vehicle matched injections in control groups. We chose i.p. injection over oral gavage primarily for reasons of dosing accuracy and animal welfare, because late-stage R6/2 mice often exhibit significant motor impairments and swallowing difficulties, which can make daily oral gavage a stressful procedure and lead to unreliable drug administration. The i.p. injections ensured that each animal received the precise intended dose.

### Two-photon in vivo imaging setup and timeline

The two-photon (2PM) in vivo imaging was performed using SP5 upright laser scanning microscope (Leica Microsystems) integrated with MaiTai Ti:Sapphire tunable infrared laser (Spectra-Physics). The images were acquired using an HCX APO L 20 × 1.0 NA water immersion objective. The estimated planar optical resolution was ~0.53 μm (*xy*), and the depth resolution was ~2.5 μm (all estimates for λ_excitation_ = 870 nm). The fluorescence signal was first split by FITC/TRITC dichroic mirror and collected after 525–560 nm/560–625 nm bandpass filters by two separate NDD multi-alkali photomultipliers (Leica Microsystems). The images were recorded using LAS AF v. 2.4 software (Leica Microsystems) in 16-bit color depth and exported to ImageJ for analysis (v. 1.52a; NIH). 3D reconstructions were performed using volume rendering in Amira v. 6 (FEI Visualization Sciences Group). All transcytosis (AMT) and NaFluo leakage imaging was performed sequentially in the same animals. AMT was assessed first using BSA-Alexa488, which produces discrete, low intensity punctate signals, followed by evaluation of paracellular permeability with NaFluo, which generates a diffuse parenchymal signal several orders of magnitude brighter. Accordingly, laser power and detector sensitivity were reduced for NaFluo acquisition, effectively suppressing any residual BSA signal to background levels. NaFluo imaging was initiated 30 min. after completion of BSA imaging. This approach enabled direct comparison of AMT and paracellular permeability within the same animals. As an internal control, TRITC-dextran was intravenously injected to confirm preserved barrier integrity and exclude any potential imaging-induced damage that could confound NaFluo measurements.

### Imaging BCSFB/BBB paracellular permeability in vivo

The paracellular permeability assays were performed as described before^11,29^ with minor modifications. Briefly, each animal was bolus-injected with 1% sodium fluorescein (NaFluo; 0.376 kDa; Sigma-Aldrich) at the dosage of 40 µL/20 g_BW_ via an arterial catheter. Immediately after (<30 s), a hyperstack (*z-*stack over time; 4D imaging) was recorded at λ = 920 nm excitation for *t* = 30 min, in bi-directional mode, encompassing the cortical volumes of 775.76 µm × 775.76 µm × 190 µm (*xy* pixel resolution = 1024 × 1024, *z* depth = 38 planes, *z* step = 5 µm), with 1 min interval between consecutive *z-*stacks.

### Quantifying BCSFB/BBB paracellular permeability

To correct for the tissue micromovement during the recording, the time-lapse images were first stabilized. The hyperstack data was exported to ImageJ and underwent dimensionality reduction by maximum intensity projection along the Z (depth) axis. The resulting timelapse image was processed using the *Image Stabilizer* plugin^124^, with *Template Update Coefficient* = 0.9;* Maximum Iterations* = 20;* and Error Tolerance* 0.0000001, to obtain the *xy* transformation coefficients (vector of tissue movement).

Next, using stabilized maximum projection as an anatomical reference^99^, small circular regions of interest (ROIs) were placed uniformly across the brain parenchyma (pROIs) where the signal from vessels was absent both in *xy* and along the *z* axis (Supplementary Fig. 1a). The pROIs’ positions were saved for subsequent use. Then, 10 circular ROIs were placed at the lumen of different pial vessel segments (vROIs) to obtain the average readout of the intensity of fluorophore circulating in the bloodstream over time (Supplementary Fig. 1b).

To obtain the readout from brain parenchyma with respect to the vascular niche, the original hyperstack was divided into three separate volumes, i.e., superficial *sub-pial* volume (0–60 µm under pia) containing pial and penetrating/ascending vessels; *intermediate* volume (60–120 µm under pia) containing all types of the brain microvasculature, and *capillary bed* volume (120–180 µm under pia) containing primarily the capillaries. Each of these volumes underwent average intensity projection in Z, and for each animal, we analyzed the average projection of *sub-pial* and *capillary bed* volume over time.

We then applied previously obtained *xy* transformation coefficients to stabilize each volume equally. Subsequently, we extracted the baseline fluorescence (at *t* = 0 min) and obtained the readout of fluorescence increase over time. The data was collected from previously identified pROIs (Supplementary Fig. 1a), and averaged within each animal. To normalize for systemic tracer exposure, the NaFluo fluorescence increase measured in each parenchymal volume at every time point was divided by the area under the complete 30-min blood-fluorescence curve obtained from the vROIs (Supplementary Fig. 1b). The area under the resulting normalized parenchymal time course was then calculated as the permeability index, equivalent to the ratio of parenchymal-signal AUC to blood-signal AUC.

### Imaging adsorptive-mediated transcytosis in vivo

We assessed the degree of adsorptive-mediated transcytosis (AMT) by imaging the endothelial uptake of the bovine serum albumin (BSA) conjugated to Alexa Fluor488 fluorescent dye (BSA-Alexa488; Invitrogen; emission in green). BSA-Alexa488 was administered i.v. as a single bolus injection (1% in saline; 2 μL/g animal) via a femoral arterial catheter. Immediately after, a hyperstack (*z-*stack over time; 4D imaging) was recorded at *λ* = 920 nm excitation, in bi-directional mode and with triple frame averaging, for *t* = 120 min, encompassing the cortical volumes of 387.5 µm × 387.5 µm × 138 µm (*xy* pixel resolution = 2048 × 2048, *z* depth = 55 planes, *z* step =  2.52 µm), and with 7.5 min interval between consecutive *z-*stacks. Over time, the BSA-Alexa488 appeared at the BBB interface in the form of bright fluorescent puncta, which corresponded to its vesicular uptake (endocytosis). Following that (at *t *= 135 min), we injected 70 kDa tetramethylrhodamine isothiocyanate-dextran (1% in saline; TRITC-dx; Sigma-Aldrich; emission in red) to delineate a vessel lumen and better distinguish between BSA-Alexa488 in circulation (overlapping with TRITC-dx) and BSA-Alexa488 puncta (Fig. 2a, c). Lack of TRITC-dx extravasation to the tissue also served as an internal control for the physical integrity of the BBB following the cranial window microsurgery and imaging procedures.

### Quantifying adsorptive-mediated transcytosis

AMT imaging hyperstack data was exported to ImageJ and an anatomical map was generated, where all individual vessel segments in the field of view were indexed to one of the following vessel types: pial arterioles (*pA*); penetrating arterioles (*penA*); capillaries *(cap)* post-capillary venules and ascending venules (*pcV/ascV*), pial venules *(pV)* and interconnecting sinuses *(sin)* (Fig. 2b). This anatomical segregation was based on the vessel location, morphology, branching pattern, diameter, and direction of the blood flow, as previously described^8,11,125^.

At 135 min after BSA injection, we performed (i) vessel surface measurements and (ii) AMT puncta count to obtain (iii) AMT puncta surface density, which corresponds to the extent of AMT.

Ad (i): each vessel segment was traced in three dimensions using ImageJ *3D measure length* macro (available at http://imagej.net/macros; Jan 2024). The distances were traced manually, always choosing the center of the vessel lumen as an anchor location for the measurement points. For each vessel segment, the vessel lumen diameter was then measured at 3-to-5 locations distributed equally along the vessel length (*Line Tool*, ImageJ). The measurements were averaged to obtain a mean representative readout. Both vessel length and diameter were then used to calculate the surface of each respective vessel segment.

Ad (ii): the puncta density was manually counted in 3D for each vessel segment in the field of view (*Point* Tool, ImageJ) using the last frame of a recorded hyperstack, i.e., after injecting TRITC-dx into circulation to delineate vessel lumen (at *t *= 135 min). The selection criteria for a positive identification of the puncta was the presence of the BSA-A488 signal (emission in green) and the absence at the exact location of the signal from other sources (emitting in red), which would indicate that the observed puncta does originate from BSA-A488; but from autofluorescent debris, e.g., lipofuscin in the brain (Fig. 2c).

Ad (iii) To calculate AMT puncta density for distinct vessel types, we summed up the puncta count of each respective vessel type and divided it by a sum of their surfaces and for each mouse. This was to eliminate a bias of averaging the density values obtained, e.g., per every single vessel, where one “AMT”-dense vessel with a small surface may skew the results of the whole vessel population. The AMT puncta density was ultimately expressed as an average number of AMT puncta per μm² of the surface of a vessel wall delineated by TRITC-dx.

### Cerebrospinal fluid collection

A 1 mL syringe connected to a polyethylene–tubing–linked glass capillary (outer diameter ≤80 µm) was used for fluid aspiration. Mice were deeply anesthetized with i.p. injection of xylazine (10 μg/g _BW_) and ketamine (10 μg/g _BW_), and mounted onto a stereotaxic frame with a 30° downward head tilt to expose the posterior neck. Following a midline incision and retraction of the overlying muscle, the dura overlying the cisterna magna was gently cleared using sterile PBS and dried with a cotton swab. The capillary was inserted between surface vessels into the cisterna magna. Cerebrospinal fluid (CSF) was slowly withdrawn while minimal negative pressure was applied to minimize the risk of contamination during collection. Samples were expelled by an air buffer pre-loaded in the syringe and snap-frozen in low-binding Eppendorf tubes on liquid nitrogen and stored at −80 °C for further analyses.

### Mass spectrometry analysis

Samples were stored at −80 °C until sample preparation for MS analysis. Five µL of each sample were processed in an SDC buffer (1% w/v sodium deoxycholate, Sigma-Aldrich; 40 mM 2-chloroacetamide, Sigma-Aldrich; 10 mM Tris(2-carboxyethyl)phosphine hydrochloride, Sigma-Aldrich; 100 mM Tris, Sigma-Aldrich; MS-grade water, VWR). Proteins were denatured at 95 °C for 10 min. at 1200 rpm. Enzyme was added at 1:100 (w/w) using a master mix of Trypsin (T6567; Sigma-Aldrich) and LysC (125-02543; Fujifilm, WAKO) enzymes, and incubated for 2 h at 37 °C. The reaction was quenched by adding quenching buffer (MS-grade isopropanol, Fisher Scientific; 1% w/w trifluoroacetic acid, Sigma-Aldrich) to each sample (pH 2). The samples were transferred to Stagetips containing two layers of SDB-RPS material (Empore) for clean-up. The peptides were washed once with quenching buffer and twice in wash buffer (0.2% TFA, Sigma-Aldrich; MS grade water, VWR) and subsequently dried. The cleaned peptides were eluted using a basic elution buffer (1% ammonia, Merck; 80% MS-grade acetonitrile, Merck; MS-grade water, VWR) and vacuum-dried to complete dryness at 45 °C. Samples were resuspended in an MS buffer (0.1% TFA, Sigma-Aldrich; 5% MS-grade acetonitrile, Merck; MS-grade water, VWR). Peptide concentration was measured with a Nanodrop 2000 and adjusted before injection on the mass spectrometer. Samples were analyzed without immunoaffinity depletion or albumin subtraction. Plasma and CSF were processed by direct digestion to avoid co-depletion of albumin-associated and other non-target proteins, which may introduce quantitative bias, reduce biomarker recovery, and affect proteome depth and cross-biofluid comparability in low-input mass spectrometry workflows^126–128^.

Two hundred ng of peptides was loaded onto Evotip C18 trap columns (Evosep Biosystems) according to the manufacturer’s instructions. LC–MS/MS analysis was performed on an Orbitrap Astral mass spectrometer (Thermo Fisher Scientific) coupled to an Evosep One system (Evosep Biosystems). Peptides are eluted online from the EvoTips using a 30 SPD (samples per day) method corresponding to a 44-min gradient using a commercial 80 mm analytical performance column (Evosep Biosystems). The column temperature is maintained at 55 °C and interfaced with an EASY-Spray Source. For DIA experiments, the Orbitrap Astral mass spectrometer is operated at a full-MS resolution of 240,000 with a full scan range of 380–980 m/z using an automatic gain control (AGC) target of 500% and a maximum injection time of 3 ms. Fragment ion scans (MS/MS scans) are set to 200 windows of 3-Th with a scan range of 150–2000 m/z and a max IT of 5 ms. The isolated ions are fragmented using HCD with 25% Normalized Collision Energy.

Raw files are processed in Spectronaut version 19.5.241126.62635 (Biognosys) in directDIA mode, analyzed against a Mus musculus FASTA file (downloaded from Uniprot on 28-10-2024). Enzyme specificity is set to Trypsin/P, and peptides are filtered to between 7 and 52 amino acids in length, with a maximum of 2 missed cleavages. Carbamidomethyl on cysteine is allowed as a fixed modification, and oxidation on methionine and acetyl on the N-terminal as variable modifications. The false discovery rate (FDR) is set at 0.01 on all levels, and cross-run normalization is set to local normalization with row selection by complete identification across all runs (‘Qvalue complete’). Proteomic data processing, including normalization, filtering, statistical analysis, and visualizations were performed in Python, utilizing an in-house analysis pipeline based on the Clinical Knowledge Graph (CKG)^129^. The data was filtered for at least 70% valid values in at least one group, and missing LFQ values were imputed by using a mixed model combining probabilistic minimum imputation and k-nearest neighbor (downshift = 1.8 standard deviation and width = 0.2, knn_cutoff = 0.6, n_neighbors = 5)^129^. Protein intensities were transformed to a *log*2 scale and normalized by the median of each sample.

### Electrophysiology setup, ECoG, and systemic physiology

Brain electrophysiological activity was recorded using a heat-pulled single-barreled borosilicate glass microelectrode (tip Ø = 2–3 μm; inner Ø = 0.86 mm; outer Ø = 1.5 mm; electrode resistance 1.5–2.0 MΩ; Sutter Instrument) with an Ag/AgCl filament, filled with artificial CSF (aCSF). The electrode was inserted 50–70 μm into the cerebral cortex under a glass coverslip, while the reference electrode was placed between the neck skin and muscle (Fig. 5a). The total electrical signal (TES) was filtered with a 0–3000 Hz low-pass filter and amplified 10x (AP311 analog amplifier; Warner Instruments). The spontaneous brain activity, or alternate current-ECoG (AC-ECoG) component, was further amplified 100x and filtered with a 0.5 Hz high-pass filter (NL 106 analog amplifier and NL 125/126 analog filter, NeuroLog). Next, the analog data was digitized at 20 kHz using a Power 1401 interface (CED). Both EcoG and systemic physiology data, i.e., the raw readout from the exhaled CO_2_ (Capnograph Type 340, Harvard Apparatus) and MABP (BP-1 Pressure Monitor, World Precision Instruments), were recorded via a Power 1401 interface (CED) in Spike2 software (v. 7.02a; CED).

### Whisker somatosensory stimulation and LFP

The right mouse sensory barrel cortex was activated by thalamocortical stimulation of the contralateral ramus infraorbitalis of the trigeminal nerve, using custom-made, percutaneously inserted bipolar electrodes, as described before^130,131^, with modification. Briefly, the cathode was positioned at the left hiatus infraorbitalis (i.o.) nerve, with the anode in the masticatory muscles (Fig. 5a). Stimulation was delivered via an ISO-flex stimulator (A.M.P.I.) at an intensity of 1.5 mA for 1 ms in trains of 15 s at frequencies in Hz: 0.5; 1.0; 2.0; 3.0. and 5.0. To minimize the impact of the preceding train of stimulation, the consecutive train stimulation was spaced 1 min apart. This round of stimulation was repeated thrice for each animal, totaling 15 stimulation trains across five stimulation frequencies per animal. From each single-frequency train, we obtained the average local field potential (LFP) readout (discarding the first stimulus impulse). From each LFP readout, we extracted the baseline (50 ms preceding each stimulus artifact spike), the evoked LFP post-synaptic excitatory potential (EPSP), post-synaptic inhibitory potential (IPSP), and their respective latencies (time from the stimulation impulse to the peak of EPSP and IPSP). Next, all values (EPSP, IPSP, EPSP and IPSP latencies) were averaged across three stimulation rounds. The sum of EPSPs and IPSPs for each frequency was calculated by multiplying the average EPSP and IPSP values by the respective stimulation frequency (0.5; 1.0; 2.0; 3.0. or 5.0 Hz) and by the duration of the stimulation train (15 s). For each electrophysiological endpoint, frequency-series data were analyzed at the level of individual mice using a two-way mixed-effects model with animal group and stimulation frequency as fixed effects and mouse as the matched repeated factor.

### SR101 astrocyte density measurements in vivo

To label the astrocytes in vivo, we loaded a heat-pulled single-barreled borosilicate glass micropipette (tip Ø = ~4 μm; inner Ø = 0.86 mm; outer Ø = 1.5 mm; electrode resistance ~1.0 MΩ; Sutter Instrument) with 1 mM sulforhodamine 101 dye (SR101; Sigma-Aldrich) in aCSF^39^. The micropipette was inserted into the cortex at a depth of ~150 µm, and SR101 was extruded in a series of 4–5 intermittent pulses (2-s pulse; 10–15 psi; pneumatic picopump PV830; World Precision Instruments). After 40 min, the SR101 uptake clearly labeled astrocyte somata and processes. To count the average density of astrocytes for each animal, we performed a series of *z-*stacks, encompassing the cortical volume of 387.5 µm × 387.5 µm × 159.7–304.4 µm range (XY pixel resolution = 1024 × 1024, Z depth = 32–61 planes, Z step = 5 µm). The *z-*stacks were collected at λ_excitation_ = 880 nm in a series of three-to-four stacks with increasing laser intensity between consecutive stacks, e.g., 3%; 6%; 12.5%, then 25% of the output power 2.51 W measured at the laser. This was to obtain a high dynamic range of recorded fluorescence signal, which permitted clear identification of astrocytes in vivo, especially at the same imaging plane with distinct astrocytes exhibiting varying degrees of SR101 uptake. Next, from the total imaged volume, we excluded the volume occupied by pial vessels and the tissue beneath along the whole *z* axis (ImageJ). This was because the presence of highly absorbing pial vessels in the tissue might obstruct the detection of astrocytes located beneath vessels in deeper cortical layers (>250 µm). The number of astrocyte somata was manually counted in 3D using *z-*stacks collected with different laser power (ImageJ) and expressed as the density of astrocytes/mm^3^ volume of the brain parenchyma.

### Immunohistochemistry in mice

Mice were anesthetized using xylazine/ketamine and transcardially perfused at a rate of 10 mL/min with phosphate-buffered saline (PBS) for 1–2 min., followed by ice-cold 4% paraformaldehyde (PFA) in PBS for 4–5 min. Brains were post-fixed in 4% PFA at 4 °C for 24 h and subsequently cryoprotected in 25% sucrose with 0.1% sodium azide in PBS at 4 °C for an additional 24 h. The brains were then frozen with dry ice and sectioned at 30 μm thickness with a sliding microtome (Microm HM450, Thermo Fisher Scientific).

For immunofluorescence staining, brain sections were initially washed in PBS to remove the antifreeze solution. For Claudin 5 immunostaining, to unmask the epitopes, brain sections underwent heat-induced epitope retrieval in 0.01 M citrate buffer (pH 6.0) at 80 °C for 30 min., then rinsed twice with PBS. Next, the sections were blocked for 1 h at room temperature (RT) in PBS containing 5% normal donkey serum (NDS) and 0.3% Triton-X-100. Sections were incubated overnight at RT for GFAP and at 4 °C for Claudin 5 in PBS with 0.025% Triton-X-100 (PBST) and 3% NDS containing the following primary antibodies: anti-glial fibrillary acidic protein (GFAP; 1:500, Agilent Technologies (Dako), Z033429-2), claudin 5 (monoclonal antibody (4C3C2), 1:100, Thermo Scientific 35-2500), anti-fibronectin antibody to label the basement matrix (fibronectin, 1:200, mouse monoclonal, ab6328), anti-CD31 (1:400, BD Biosciences, BD550274) to label endothelial cells, anti-α-smooth muscle actin (anti-actin α-smooth muscle - Cy3™ mouse monoclonal antibody, 1:250, Sigma-Aldrich, C6198) for vascular smooth muscle cells. Sections were then washed (3× for 10 min in PBST) and incubated for 1 h at RT with fluorescent secondary antibodies: Alexa Fluor 488 (1:500, goat anti-rat, Abcam, ab150157 or goat anti-mouse, ThermoFisher Scientific, #A32731TR), and Alexa Fluor 647 (1:500, donkey anti-rabbit, Abcam, ab150075), all diluted in PBST with 5% NDS. After another round of washes (3× for 10 min in PBST), sections were stained with DAPI (1:1000, ThermoFisher Scientific) for 10 min. to visualize cell nuclei, followed by a final wash in PBS. The stained sections were mounted on gelatin-coated glass slides and coverslipped using an antifading mounting medium (Fluoroshield Mounting Medium with Propidium Iodide, Abcam, ab104129).

### Confocal imaging of claudin-5 in mice

Claudin-5 fluorescence in the cortical microvasculature was imaged using a DMi8 inverted confocal microscope (Leica Microsystems) equipped with an HC PL APO 40×/1.3 NA oil-immersion objective. The images were acquired using 405, 488, 552, and 638 nm laser diodes for DAPI, Alexa Fluor 488, Cy3, and Alexa Fluor 647, respectively. The fluorescence signal was collected with a hybrid detector using 440–480, 500–550, 565–585 and 655–685 nm bandpass filters. *z-*stacks were acquired in sequential mode with triple line averaging, 2048 × 2048 pixel resolution, at 2 airy units and 2 μm *z-*step intervals, corresponding to a field of view of 386 × 386 μm and an average stack depth of ~30 μm. The images were exported as multichannel 16-bit TIFF files and analyzed in ImageJ. Because of the complex geometry, particularly in large vessels, claudin-5 signal was quantified in the Alexa Fluor 488 channel from maximum-intensity projections of ~10–14 μm substacks and measured at BEC contact sites corresponding to tight junctions (TJs), rather than along the vessel wall. For each analyzed segment, a ribbon-shaped ROI was placed at the TJ site, and a parallel ROI of identical dimensions was positioned immediately adjacent to it, outside the TJ region, to estimate local background. The specific claudin-5 signal was calculated by subtracting the local background from the TJ signal. ROI width was fixed at 0.6 μm, whereas ROI length varied with the analyzed segment. Across all analyzed animals (*n* = 15; 5 mice per group; 5 *z-*stacks per animal), the cumulative length of analyzed contact sites was ~15 mm for arterioles, ~25 mm for capillaries, and ~11 mm for venules. For each mouse, the mean claudin-5 signal was calculated as a length-weighted average across analyzed segments, so that longer segments contributed proportionally more than shorter ones and the resulting value more accurately reflected the overall property of the segment population.

### Confocal imaging of GFAP+ astrocytes in mice

Widefield fluorescence images were acquired on an Axio Scan.Z1 slide scanner (Zeiss) using ZEN 3.1 in tiled mode, with a Plan-Apochromat 10×/0.45 M27 air objective and an Axiocam 506 mono camera. CD31-Alexa Fluor 647, GFAP-Alexa Fluor 488, αSMA-Cy3, and DAPI were imaged as 4-channel *z-*stacks spanning 9 optical planes at 2 μm intervals and 0.911 × 0.911 μm xy resolution. Tiles were acquired with 10% overlap, stitched in ZEN, and exported as multichannel 12-bit TIFF files for analysis in ImageJ.

### Immunohistochemistry in human brains

Sections were placed on a slide warmer at 65 °C for 20 min. to remove the paraffin, followed by defatting in Citrisolv (Fisher Scientific, 22143975) and rehydration in a descending bath of ethanol solutions. Sections were then rinsed with MilliQ water before antigen retrieval was performed with a solution of citrate buffer (sodium citrate tribasic 10 mM [MilliporeSigma, W302600], EDTA 2 mM [Fisher Scientific, BP-2482], Tween-20 0.05% [Fisher Scientific, BP-337]) pH 6.2 at 80 °C for 20 min. After a cooling period of 20 min. at RT in citrate buffer, sections were washed with potassium phosphate-buffered saline (KPBS), incubated in 3% peroxide for 30 min., and then immersed in blocking solution (1% bovine serum albumin [BSA, BioShop Canada, cat# ALB001], 0.1% Triton X-100 [MilliporeSigma, cat# T8787], 5% NDS [MilliporeSigma, D9663] in KPBS) for one hour at RT. After additional washes, sections were incubated overnight at 4 °C with a mix of primary antibodies in blocking solution; goat anti-collagen IV (1/500; MilliporeSigma, AB769), rabbit anti-GFAP (1/500; Dako, Z0334) and mouse anti-αSMA conjugated with Cy3 (1/200; MilliporeSigma, C6198). The following day, sections were washed with KPBS and incubated with secondary antibodies diluted in blocking solution; donkey anti-goat Alexa Fluor 488 (1/500; Invitrogen, A11055) and donkey anti-rabbit Alexa Fluor 647 (1/500; Invitrogen, A31573) for 2.5 h at RT. After subsequent washes in KPBS, sections were incubated with DAPI (1/5000, DAPI 5 mg/mL, Invitrogen, D3571) for 10 min., washed in KPBS and autofluorescence was quenched using a solution of Sudan Black (MilliporeSigma, 199664) 0.1% in 70% ethanol. Sections were washed in KPBS to remove the excess of Sudan Black, rinsed in MilliQ water and coverslipped with Fluoromount-G (Invitrogen, 00-4958-02). The list of human brains and patient demographics was included in Supplementary Data 5.

### Whole-slide imaging of human brain sections

Scans of the entire section stained for each case were acquired with an Axio Scan.Z1 slide scanner (Zeiss) using ZEN 3.1 in tiled mode, with a Plan-Apochromat 20×/0.8 M27 objective and a Hamamatsu Orca Flash 4.0 camera. DAPI, Alexa Fluor 488, Cy3, and Alexa Fluor 647 were imaged as 4-channel *z-*stacks spanning 3 optical planes at 2.5 μm intervals and 0.325 × 0.325 μm xy resolution. Fluorophores were excited by LED modules at 385 nm, 470 nm, white-light, and 625 nm, with channel-specific reflector and filter configurations (63 HE Red Fluorescent Protein, 38 HE Green Fluorescent Protein, 43 HE DsRed, and 50 Cy5; beamsplitters 590, 495, 570, and 660; filter ranges 559–585/600–690, 450–490/500–550, 538–562/570–640, and 625–655/665–715 nm). Images were acquired at 16-bit depth and were exported for subsequent analysis in ImageJ.

### Statistical analyses

The sample sizes, including the treatment group size, were estimated based on our previous 2PM studies of paracellular permeability, transcytosis, and electrophysiology in vivo^8,11,130^. The in vivo experiments were performed to obtain readouts of cerebral and systemic electrophysiology, AMT, paracellular permeability, and astrocyte count, in sequence from each imaged animal. All statistical analyses were made comparing independent biological replicates, i.e., individual patients and mice.

For comparisons between the groups, the data were first tested for normality using the D’Agostino-Pearson test. For 2PM and IHC datasets, normally distributed data were analyzed using two-sided Student’s *t* tests when variances were similar and two-sided Welch’s t-tests when variances differed; whereas non-normally distributed data were analyzed using two-sided Mann–Whitney tests. For testing multiple hypotheses, Bonferroni post-hoc correction was used. For electrophysiological datasets, we used a two-way mixed-effects model (REML) in Prism v.10.2 (GraphPad), with animal group (WT, R6/2, R6/2+ ) as the between-subject factor and stimulation frequency (0.5, 1, 2, 3, and 5 Hz) as the within-subject repeated factor, with repeated measurements matched by mouse. The model included the group, frequency, and group × frequency interaction terms, and Šídák-corrected multiple comparisons were used for simple effects within each stimulation frequency. The statistical analyses were performed using Prism v.10.2 (GraphPad). Data were plotted using Prism v.10 and OriginPro 2018 (OriginLab Corporation). No outlier testing and elimination were performed to avoid inflation of type I error rates. Proteomic identification of differentially expressed proteins between different groups was executed using t-tests with permutation-based threshold false-discovery rate (FDR) < 0.05 and S_0_-value = 0.3 at 250 randomizations for each comparison^129,132^. For all datasets in the study, the results were considered statistically significant for *p-*values or corrected *p* < 0.05. The data were presented as average ± standard error of the mean (SEM), unless stated otherwise.

The imaging experiments could not be performed in a blinded manner due to overt differences between WT and R6/2 phenotypes, but the imaging procedures and data acquisition parameters were the same across all tested groups. The electrophysiology, paracellular permeability, AMT, and cell-count analyses were performed in semi-randomized order (by the ascending ID number of the animal) by an investigator blinded to experimental conditions. The analyses involving immunohistochemistry were performed in a blinded manner for both mice and human brains, with imaging acquisition parameters the same across tested groups. We used a pooled group of both sexes for quantitative IHC comparisons after ad hoc analyses revealed no overt differences in staining between males and females.

### Exclusion criteria

One out of 22 animals was excluded from in vivo electrophysiological recordings due to electrode noise obscuring the ECoG and LFP readouts. All 2PM recordings exhibiting significant (>2 μm/min) brain movement in X, Y, or Z coordinates or loss of focus during timelapse imaging were excluded from analyses (two out of 25 for paracellular permeability; one out of 25 for AMT). For mass spectrometry analyses, two mice from the plasma group were excluded due to low protein yield and protein identifications.

### Model considerations

We chose the R6/2 transgenic mouse model of HD, which carries the human mutant huntingtin gene^16,17,115^. This model is characterized by early onset and rapid disease progression^16,17^ and replicates key features of HD, including the distribution of mHTT aggregates across neurovascular unit (NVU) cell types^20^ and transcriptional changes similar to those observed in patients^133^. The transgene is expressed under the control of the human huntingtin promoter, achieving ~75% of endogenous huntingtin levels^27,134^. While the R6/2 model does not capture all aspects of HD, its robust phenotype and reproducibility make it a widely accepted platform for studying disease mechanisms and therapeutic interventions. Importantly, mHTT is detected in all major NVU components^19,20^, mirroring its widespread vascular deposition in human HD pathology. See also Supplementary Notes detailing model choice rationale.

### Methodological considerations

The 2PM allowed us to assess changes in microvascular function in the living, intact brain with superior spatial and temporal resolution over whole-brain imaging techniques such as magnetic resonance imaging or positron emission tomography. However, our method also has limitations. First, the diffusion rate of NaFluo in the brain was too fast to identify whether arterioles or venules contributed most to paracellular leakage in the sub-pial region. Still, it is unlikely that NaFluo leaked at capillaries and diffused along larger surface vessels via perivascular spaces to sub-pial volume. If this was the case, we would observe a concentration gradient with high NaFluo presence at capillaries and low levels in sub-pial volumes, which was not present in the analyzed images. Notably, earlier studies using Evans Blue did not account for its non-selective nature. An increase in Evans Blue accumulation within the brain parenchyma may not indicate increased paracellular permeability but could result from increased AMT, as Evans Blue also binds to albumin in the bloodstream^135^. Here, we selectively focused on albumin to get insight into AMT. Although albumin is the main protein undergoing AMT, other proteins, such as IgGs, can also follow this route^10^. Despite that, compared to transmission electron microscopy, which is typically performed on capillaries due to preservation challenges with larger vessels, our method allowed for the first detailed estimation of AMT in large vessels in HD, avoiding the oversights of ex vivo studies or cell cultures that lack the brain’s functional and structural heterogeneity. Second, 2PM is inherently limited in penetration depth to a few hundred micrometers in the brain tissue. While imaging up to ~250 μm is a reasonable range covering 1/4 of the cortical thickness, it is still less than the depth of the core HD-affected region, i.e., striatum ( > millimeters below the surface). Imaging the striatum would require either cortex resorption or implanting invasive optical probes (e.g., gradient-index microendoscope lenses). While such approaches have been tested for deep brain imaging^136,137^, the implantation disrupts the tissue and blood vessels via transection of microvessels and compression of neighboring tissue, leading to bleeding and reactive vascular remodeling that can take weeks to stabilize, all confounding the measures of barrier permeability^138^. Moreover, in contrast to the short imaging span during acute preparations herein, the implanted chronic lenses elicit glial scarring (∼50 μm thick), with ongoing astrogliosis in the affected zone^139^. Choosing this approach could confound the data. Lastly, it cannot be excluded that craniotomy and dura removal might alter local vascular or inflammatory conditions differently in R6/2 and WT brains, and the use of e.g., thinned-skull imaging, or closed cranial window preparations, or alternative experimental imaging approaches could mitigate this potential issue. However, the arteriolar predominance of the phenotype, rather than a stronger venular involvement expected from an inflammatory response, together with concordant mouse and human histology and genotype- and treatment-dependent proteomic signatures, argues against the findings being solely attributable to the microsurgical preparation for imaging.

### Reporting summary

Further information on research design is available in the Nature Portfolio Reporting Summary linked to this article.

## Supplementary information

Supplementary Information

Description of Additional Supplementary Files

Supplementary Data 1

Supplementary Data 2

Supplementary Data 3

Supplementary Data 4

Supplementary Data 5

Supplementary Data 6

Supplementary Data 7

Supplementary Movie 1

Supplementary Movie 2

Supplementary Movie 3

Supplementary Movie 4

Reporting Summary

Transparent Peer Review file

## Source data

Source Data

## Data Availability

All numerical data and full statistical outputs are in Supplementary Data. Source data are provided with this paper in the Source Data file, and were deposited under Findable, Accessible, Interoperable and Reusable (FAIR) principle in Zenodo open access depository (https://doi.org/10.5281/zenodo.21399792). The mass spectrometry proteomics data have been deposited to the ProteomeXchange Consortium via the PRIDE partner repository with the dataset identifier PXD081397. Color-vision-accessible versions of all figures were deposited on Figshare (https://doi.org/10.6084/m9.figshare.33069248). Owing to their multi-terabyte volumes, the raw imaging datasets are available from the corresponding author upon request. Source data are provided with this paper.
