# Supplementary material for: Huntington’s disease vasculopathy is barrier-type-selective and arteriolar-dominant with spatially-coupled astrogliosis and reversible by S1pr1 stimulation
Source: Nat Commun. 2026 Sep 12;17:10775. doi: 10.1038/s41467-026-77474-4 (PMC13661524; doi:10.1038/s41467-026-77474-4)
Supplement: Supplementary file 1 [file 41467_2026_77474_MOESM14_ESM.pdf]

nature portfolio

Corresponding author(s): Krzysztof Kucharz (kucharz@sund.ku.dk)

Last updated by author(s): 16/8/26

Reporting Summary

Nature Portfolio wishes to improve the reproducibility of the work that we publish. This form provides structure for consistency and transparency in reporting. For further information on Nature Portfolio policies, see our Editorial Policies and the Editorial Policy Checklist.

Statistics

For all statistical analyses, confirm that the following items are present in the figure legend, table legend, main text, or Methods section.

n/a	Confirmed	
☐	☒	The exact sample size (*n*) for each experimental group/condition, given as a discrete number and unit of measurement
☐	☒	A statement on whether measurements were taken from distinct samples or whether the same sample was measured repeatedly
☐	☒	The statistical test(s) used AND whether they are one- or two-sided<br>*Only common tests should be described solely by name; describe more complex techniques in the Methods section.*
☐	☒	A description of all covariates tested
☐	☒	A description of any assumptions or corrections, such as tests of normality and adjustment for multiple comparisons
☐	☒	A full description of the statistical parameters including central tendency (e.g. means) or other basic estimates (e.g. regression coefficient) AND variation (e.g. standard deviation) or associated estimates of uncertainty (e.g. confidence intervals)
☐	☒	For null hypothesis testing, the test statistic (e.g. *F*, *t*, *r*) with confidence intervals, effect sizes, degrees of freedom and *P* value noted<br>*Give P values as exact values whenever suitable.*
☒	☐	For Bayesian analysis, information on the choice of priors and Markov chain Monte Carlo settings
☒	☐	For hierarchical and complex designs, identification of the appropriate level for tests and full reporting of outcomes
☐	☒	Estimates of effect sizes (e.g. Cohen's *d*, Pearson's *r*), indicating how they were calculated

Our web collection on statistics for biologists contains articles on many of the points above.

Software and code

Policy information about availability of computer code

Data collection	Data were collected using LAS AF v.2.4 (Leica Microsystems) for in vivo two-photon microscopy, Spike2 v.7.02a (CED) for electrophysiology and systemic physiology acquisition, ZEN 3.1 (Zeiss) for Axio Scan.Z1 image acquisition, and Spectronaut v.19.5.241126.62635 (Biognosys) in directDIA mode for mass-spectrometry raw-file processing. LC–MS/MS acquisition was performed on an Orbitrap Astral mass spectrometer coupled to an Evosep One system, and peptide concentration was measured with a Nanodrop 2000.
Data analysis	Data were analyzed in ImageJ v.1.52a (NIH) for image analysis, including movement correction and quantification; Amira v.6 (FEI Visualization Sciences Group) for 3D reconstructions; Prism v.10.2 (GraphPad) and OriginPro 2018 (OriginLab) for statistical analyses and plotting; and Python using an in-house Clinical Knowledge Graph (CKG)-based pipeline for proteomic normalization, filtering, imputation, statistical analysis and visualization. Raw proteomic files were processed in Spectronaut v.19.5.241126.62635.

For manuscripts utilizing custom algorithms or software that are central to the research but not yet described in published literature, software must be made available to editors and reviewers. We strongly encourage code deposition in a community repository (e.g. GitHub). See the Nature Portfolio guidelines for submitting code & software for further information.

Data

Policy information about availability of data

All manuscripts must include a data availability statement. This statement should provide the following information, where applicable:

- Accession codes, unique identifiers, or web links for publicly available datasets
- A description of any restrictions on data availability
- For clinical datasets or third party data, please ensure that the statement adheres to our policy

All numerical data and full statistical outputs are in Supplementary Data. Source data are provided with this paper in the Source Data file, and were deposited under Findable, Accessible, Interoperable and Reusable (FAIR) principle in Zenodo open access depository (https://doi.org/10.5281/zenodo.21399792). The mass spectrometry proteomics data have been deposited to the ProteomeXchange Consortium via the PRIDE partner repository with the dataset identifier PXD081397. Color-vision-accessible versions of all figures were deposited on Figshare (https://doi.org/10.6084/m9.figshare.33069248). Owing to their multi-terabyte volumes, the raw two-photon imaging datasets are available from the corresponding author upon request.

Research involving human participants, their data, or biological material

Policy information about studies with human participants or human data. See also policy information about sex, gender (identity/presentation), and sexual orientation and race, ethnicity and racism.

Reporting on sex and gender	Both sexes were included in the human cohort. The human post-mortem dataset comprised HD donors (n=13; 8 male, 5 female) and non-HD donors (n=7; 4 male, 3 female). Donor sex was obtained from biobank records; gender identity was not collected. Sex-stratified analyses were performed for the human GFAP pathology.
Reporting on race, ethnicity, or other socially relevant groupings	No race, ethnicity, or other socially constructed grouping variable was collected or analyzed in this pathology study, and no such variable was used as a covariate.
Population characteristics	Human material comprised archived post-mortem cortical grey-matter sections from HD and non-HD donors. Available donor-level variables included diagnosis, sex, age at death, post-mortem interval, CAG repeat length, and Vonsattel grade, where available.
Recruitment	No participants were prospectively recruited. Chemically-fixed cortical sections were obtained as archived post-mortem material. HD and age- and sex-matched control cases were included only when vessels and meninges were well preserved, to minimize sectioning artefacts and ensure comparable tissue preservation across cohorts
Ethics oversight	Use of human tissue was approved under CHU de Québec Research Ethics Committee approval A13-02-1138. Informed consent for research brain donation was obtained from the legal next of kin according to protocols approved by the University of Washington Institutional Review Board that conform to the provisions of the Declaration of Helsinki and preserve patient anonymity.

Note that full information on the approval of the study protocol must also be provided in the manuscript.

Field-specific reporting

Please select the one below that is the best fit for your research. If you are not sure, read the appropriate sections before making your selection.

☒ Life sciences ☐ Behavioural & social sciences ☐ Ecological, evolutionary & environmental sciences

For a reference copy of the document with all sections, see nature.com/documents/nr-reporting-summary-flat.pdf

Life sciences study design

All studies must disclose on these points even when the disclosure is negative.

Sample size	Sample sizes were estimated on the basis of previous in vivo two-photon microscopy studies of paracellular permeability, transcytosis, and electrophysiology; no formal a priori power calculation was performed. Exact n values are provided in the Figures, Figure legends, Methods, and Supplementary Tables. Experimental units were individual mice or individual human donor cases
Data exclusions	Pre-established exclusion criteria were applied. One of 22 animals was excluded from in vivo electrophysiological recordings because of electrode noise. Two-photon datasets were excluded when brain movement exceeded >2 µm/min in x, y, or z coordinates, or when focus was lost during timelapse imaging (2/25 datasets for paracellular permeability and 1/25 for AMT). For mass spectrometry, two mice from the plasma group were excluded because of low protein yield and low identifications. No outlier testing or elimination was performed.
Replication	All statistical analyses were performed on independent biological replicates, defined as individual mice or individual human donor cases. In electrophysiology, each mouse underwent three repeated rounds of somatosensory stimulation, and repeated measurements were matched by mouse in the mixed-effects analysis. Findings were supported across complementary modalities, including in vivo imaging, immunohistochemistry, proteomics, and electrophysiology, and representative images were taken from experiments showing the quantified

phenotype.

Randomization	R6/2 mice were randomly allocated to the untreated and SEW2871-treated groups while maintaining balanced male-to-female ratios across groups. Data collection and downstream quantitative analyses were performed in randomized order.
Blinding	In vivo imaging could not be performed blinded because WT and R6/2 mice showed overt phenotypic differences; however, imaging procedures and acquisition parameters were identical across all groups. Immunohistochemistry analyses were performed blinded to group identity in both mouse samples (WT, R6/2 and R6/2-treated) and human samples (non-HD and HD). Post-acquisition analyses of electrophysiology, paracellular permeability, AMT and cell counts were conducted by an investigator blinded to the experimental conditions. Because an additional R6/2-treated group was introduced during revision, group allocation was not blinded in every experiment.Nonetheless, all outcomes were based on predefined quantitative imaging, proteomic and electrophysiological analysis pipelines rather than subjective scoring.

Reporting for specific materials, systems and methods

We require information from authors about some types of materials, experimental systems and methods used in many studies. Here, indicate whether each material, system or method listed is relevant to your study. If you are not sure if a list item applies to your research, read the appropriate section before selecting a response.

Materials & experimental systems

n/a	Involved in the study
☐	☒ Antibodies
☒	☐ Eukaryotic cell lines
☒	☐ Palaeontology and archaeology
☐	☒ Animals and other organisms
☐	☒ Clinical data
☒	☐ Dual use research of concern
☒	☐ Plants

Methods

n/a	Involved in the study
☒	☐ ChIP-seq
☒	☐ Flow cytometry
☒	☐ MRI-based neuroimaging

Antibodies

Antibodies used	Mouse tissue: anti-GFAP (1:500, Agilent/Dako, Z033429-2); anti-claudin-5, clone 4C3C2 (1:100, Thermo Scientific, 35-2500); anti-fibronectin, mouse monoclonal (1:200, Abcam, ab6328); anti-CD31 (1:400, BD Biosciences, BD550274); anti-α-smooth muscle actin, Cy3-conjugated mouse monoclonal (1:250, Sigma-Aldrich, C6198). Secondary antibodies: Alexa Fluor 488 goat anti-rat (1:500, Abcam, ab150157), Alexa Fluor 488 goat anti-mouse (1:500, ThermoFisher Scientific, A32731TR), and Alexa Fluor 647 donkey anti-rabbit (1:500, Abcam, ab150075). Human tissue: goat anti-collagen IV (1:500, MilliporeSigma, AB769); rabbit anti-GFAP (1:500, Dako, Z0334); mouse anti-αSMA conjugated with Cy3 (1:200, MilliporeSigma, C6198). Secondary antibodies: donkey anti-goat Alexa Fluor 488 (1:500, Invitrogen, A11055) and donkey anti-rabbit Alexa Fluor 647 (1:500, Invitrogen, A31573).
Validation	Commercial antibodies were used in recommended species/applications and were supported by expected anatomical staining and internal controls.

Animals and other research organisms

Policy information about studies involving animals; ARRIVE guidelines recommended for reporting animal research, and Sex and Gender in Research

Laboratory animals	Laboratory animals were 23–24-week-old heterozygous R6/2 mice carrying 266–393 CAG repeats, with age-matched wild-type littermates as controls on a B6CBA background. Animals were housed in climate-controlled ventilated cages at 50 ± 10% relative humidity, room temperature, under a 12 h light/12 h dark cycle, with ad libitum access to food and water.
Wild animals	This study did not involve wild animals.
Reporting on sex	Both male and female mice were included. In the mouse GFAP immunohistochemistry cohort, each genotype comprised 2 males and 3 females. Ad hoc sex-stratified re-analysis did not reveal overt sex-dependent divergence in the mouse gliosis/pathology readouts; therefore, given the limited statistical power after stratification, the primary quantitative analyses were performed on pooled data across sex.
Field-collected samples	This study did not involve field-collected samples.
Ethics oversight	All animal experiments were approved by the Danish Animal Experiments Inspectorate (permit #2019-15-0201-01655). Experiments followed ARRIVE guidelines (incl. 4R principle) and conformed to the European Council Convention for the Protection of Vertebrate Animals Used for Experimental and Other Scientific Purposes.

Note that full information on the approval of the study protocol must also be provided in the manuscript.

Clinical data

Policy information about clinical studies
All manuscripts should comply with the ICMJE guidelines for publication of clinical research and a completed CONSORT checklist must be included with all submissions.

Clinical trial registration	This study did not involve a clinical trial.
Study protocol	No prospective interventional clinical study protocol applies, because the human component analyzed archived post-mortem tissue and associated donor metadata.
Data collection	Human data were derived from archived post-mortem cortical grey-matter sections and associated donor metadata.
Outcomes	For the human cohort, the prespecified outcomes were histopathological measurements of GFAP-positive astrocyte density and signal intensity across vessel-associated territories and cortical depth, including periarteriolar, perivenular, and capillary territories. Analyses also examined relationships to sex, CAG repeat length, Vonsattel grade, age at death, and post-mortem interval.

Plants

Seed stocks	This study did not involve plants or plant material.
Novel plant genotypes	This study did not involve generation of new plant genotypes.
Authentication	No plant material was used.
