# Supplementary material for: Huntington’s disease vasculopathy is barrier-type-selective and arteriolar-dominant with spatially-coupled astrogliosis and reversible by S1pr1 stimulation
Source: Nat Commun. 2026 Sep 12;17:10775. doi: 10.1038/s41467-026-77474-4 (PMC13661524; doi:10.1038/s41467-026-77474-4)
Supplement: Supplementary file 2 [file 41467_2026_77474_MOESM15_ESM.pdf]

nature portfolio

Peer Review File

Huntington's disease vasculopathy is barrier-type-selective and arteriolar-dominant with spatially-coupled astrogliosis and reversible by S1pr1 stimulation

Corresponding Author: Dr Krzysztof Kucharz

This file contains all reviewer reports in order by version, followed by all author rebuttals in order by version.

Version 0:

Reviewer comments:

Reviewer #1

(Remarks to the Author)
The article "Vascular dysfunction in Huntington's disease is located at the blood-CSF barrier and is rescued by a sphingosine-1-phosphate receptor 1 agonist" by Kucharz et al. presents a highly interesting and technically impressive study of vascular dysfunction in Huntington's disease (HD). The authors primarily use in vivo approaches and include two-photon imaging, electrophysiology, proteomics, and IHC in the R6/2 mouse model, with findings further supported by human IHC staining. The study demonstrates that stimulating S1pr1 with an agonist can rescue some of the observed deficits, notably by suppressing adsorptive-mediated transcytosis (AMT) in arterioles, though not in other vessel types.

Comments

R6/2 mouse model considerations:
The authors used heterozygous R6/2 mice carrying 266–393 CAG repeats, which allows for slower disease progression and longer survival. The study describes the 23-24-week-old mice as representing a late, severe disease stage. However, prior studies (e.g., Pardo et al., 2017; PMID: 28117381) have reported BBB alterations and tracer leakage consistent with BBB breakdown in late-stage R6/2 mice. Interestingly, this study did not observe significant leakage at the capillary bed, which would typically be expected at such an advanced disease stage. The authors might wish to discuss this discrepancy further. Additionally, to strengthen their conclusions and better reconcile these divergent findings, the authors could consider immunostaining for tight junction proteins (e.g., claudin-5, occludin) which could help assess potential structural alterations not detectable by tracer leakage alone. This approach could help clarify whether the absence of capillary leakage reflects a true preservation of BBB integrity or rather a limitation of the detection method used.

Molecular mechanisms and transcytosis:
While the work convincingly shows vessel-type-specific dysfunction, the underlying molecular mechanisms remain insufficiently explored. The proteomic analysis offers valuable but correlative rather than causal insights. The reported inverse relationship between paracellular leakage and AMT is particularly interesting but mechanistically unexplained. Additionally, the study focuses primarily on AMT and paracellular transport through BSA-A488 and NaFluo imaging, respectively, without directly investigating receptor-mediated transcytosis (RMT), which could provide further mechanistic depth.

Brain region focus:
HD pathology predominantly affects the striatum (including the caudate nucleus and putamen). However, the present study mainly examines cortical regions. While this choice might be due to technical considerations related to imaging, exploring vascular pathology in the striatum would improve the study's disease relevance.

Role of pericytes and VSMCs:
Although the article mentions mutant huntingtin (mHtt) aggregates in pericytes and vascular smooth muscle cells (VSMCs) within the microvascular network, the in-depth analysis of barrier dysfunction focuses mainly on endothelial cells and astrocytes. Further exploration of how pericyte or VSMC dysfunction might contribute to the observed vascular pathology would strengthen the study.

Human capillary phenotype:
The inclusion of human IHC data is a notable strength, but the manuscript does not describe in detail the capillary phenotype or whether distinct patterns of leakage were observed in the human brain samples. Providing these details would improve the translational impact of the findings.

Agonist administration details:
The manuscript states that SEW2871 was administered at 10 µg/g for six days, but it does not provide a citation or further methodological details. It would be helpful to include references for this dosing regimen and to discuss whether oral gavage or other administration routes were considered. Additionally, SEW2871 is known to have side effects that could influence its long-term utility (see Flemming et al., 2018; PMID: 28538086).

Overall, this is a well-executed and innovative study with significant potential impact on our understanding of vascular dysfunction in HD. Addressing the points above would further enhance the clarity and depth of the work.

Reviewer #2

(Remarks to the Author)
I co-reviewed this manuscript with one of the reviewers who provided the listed reports. This is part of the Nature Communications initiative to facilitate training in peer review and to provide appropriate recognition for Early Career Researchers who co-review manuscripts.

Reviewer #3

(Remarks to the Author)
Kucharz and colleagues investigate the vascular dysfunction that occurs in the R6/2 mouse model of Huntington's disease (HD). This work builds on previous observations that mutant Huntingtin aggregates in all major components of the vascular unit and this is accompanied by loss of BBB integrity in striatum and putamen, with increased transcytosis and paracellular transport (Drouin-Ouellet et al, Ann Neurol, 2015). The present work focuses on pinpointing more in detail the precise anatomical location and mechanisms of such alterations.
More precisely, Kucharz and colleagues demonstrate, via in vivo 2-photon microscopy, that solute leakage is confined to the blood–CSF barrier (BCSFB), at the large sub pial vessels, while capillary permeability remains largely intact.
On the other hand, adsorptive mediated transcytosis (AMT), that is normally suppressed in healthy conditions, is largely upregulated across the vascular bed, but to a larger extent in arterioles and penetrating arterioles.
Notably, treatment with SEW2871, an agonist of S1P-receptor-1, normalises BCSFB leakage and dampens arteriolar AMT, but leaves capillaries/venules little changed.
Astrocyte activation is congruent with the pattern of vascular alterations, being greater at the pial surface and around arterioles in both animal models and human samples of the disease.
The authors also perform a CSF proteome analysis, which shows upregulation in inflammatory and vascular mediators. Interestingly, some of these alterations were reverted by SEW2871 treatment, suggesting that the altered vascular properties can thus be reflected to some extent by the changes in CSF proteome composition.
Finally, the authors observe cortical hyperexcitability, and propose that the observed changes in vascular permeability could contribute to some of the electrical alterations seen in the superficial layers of the cortex.
Overall, this manuscript offers a detailed in vivo description of HD vasculopathy and pinpoints its alterations to specific vascular segments. Fundamentally, it also provides a potential therapeutic strategy via a druggable target, S1PR1, which has already been tested in neurodegenerative and autoimmune diseases.
The following points should be addressed before considering publication:

• Data interpretation and relevance to disease progression: despite the detailed in vivo characterization, the molecular mechanisms of such vascular alterations remain elusive. Do the authors have a hypothesis for the molecular mechanism(s) underlying vasculopathy in HD? Also, it is unclear whether vascular changes contribute at all to the pathology of HD. Given the very late time-point, it is possible that such alterations are just consequence of the extensive neurodegeneration and do not play a role in disease progression. Similarly, it is unclear whether normalizing such changes has any effect on the disease course.
• Potential preparation artifact: the paracellular leakage in the pial vessels, although specific to R6/2 model, could be exacerbated by the invasive imaging technique (craniotomy and removal of the dura). Can the authors exclude confirm the observation with a less invasive technique (e.g. histology, thin-skull preparation)
• Origin of gliosis response: increased vessel permeability and AMT could have an effect also on microglia and on recruited immune cells which, in turn, could drive the increased astrogliosis.
• Interpretation of CSF proteome results: the authors attempt to draw parallels between the changes they observe in vascular permeability and modifications in CSF proteome. The link remains elusive. Treatment with the S1PR1 agonist only partially reverts the phenotype. A more balanced description of the results is warranted.
• Interpretation of the neurophysiology results: changes in electrical excitability cannot be unequivocally ascribed to the vasculopathy (Of note changes in excitability were not checked after S1PR1 agonist treatment). Also for this point a more balanced description of the results is warranted.
• Protein concentration in proteome analysis: The authors find 1638 proteins in plasma and 4746 proteins in CSF. Is this expected, given that average protein concentration in mouse plasma is 60 mg/ml and in CSF 0,3-0,4 mg/ml (PMID 24677814; 23272901)? Also, do the authors perform any albumin removal step?

Minor points:
• The GFAP activation pattern seems to have a sex bias in human samples. Did the authors observe also sex differences in the animal model?
• The legends sometimes describe results and sometimes describe the figure (e.g. figure 4B vs figure 4D). Please choose a uniform style.
• Figure 3: please correct the legend for figure 3E and 3F
• Figure 4: please correct the legend for figure 4B and 4C.
• Extended data figure 2: please correct the typo in line 35 (show demonstrate)
• Video 4: constrictions are hard to catch for the non-expert and no comparison with control is provided. Quantification of the data would make the observation more relevant.

Reviewer #4

(Remarks to the Author)
I co-reviewed this manuscript with one of the reviewers who provided the listed reports. This is part of the Nature Communications initiative to facilitate training in peer review and to provide appropriate recognition for Early Career Researchers who co-review manuscripts.

Reviewer #5

(Remarks to the Author)
The manuscript by Kucharz et al. it is very well written and illustrated, and provides an elegant and thorough characterization of heterogeneous vascular alterations in the Huntington's disease (HD) R6/2 mouse model and in human postmortem samples. The authors demonstrate significant blood–brain barrier (BBB) and blood–cerebrospinal fluid barrier (BCSFB) dysfunction and further show that treatment with a sphingosine-1-phosphate receptor 1 (S1PR1) agonist, SEW2871, can reverse some of these alterations. These findings highlight potential therapeutic avenues for HD and represent a valuable contribution to the field.
However, major revisions are required to strengthen the validity of the model and clarify mechanistic links. In particular, the choice and characterization of the HD model, the interpretation of the S1PR1-related effects, and the integration of some experimental data require further clarification and additional evidence.

Major Comments:

1. The R6/2 mouse model used in the study carries >200 CAG repeats, which is significantly higher than the original R6/2 line and may alter the expected HD phenotype. Please provide supporting data or literature demonstrating that these mice still exhibit a robust and relevant HD phenotype, or validate some results with a more appropiate model to ensure validity of the results.

2. S1PR1 is expressed not only in brain endothelial cells (BECs) but also in other neural and non-neural cell types. Therefore, systemic S1PR1 agonism cannot be attributed exclusively to effects on BECs. Please expand the discussion to clarify potential off-target effects and the contribution of other S1PR1-expressing cells. Also, please discuss the rationale for selecting SEW2871 drug and its dose, its selectivity for S1PR1 receptor and whether other S1P receptor subtypes may be involved. How is S1PR1 expression in HD brains? Please also discuss whether additional drug effects on other brain cells could contribute to the observed results.

3. The experiments demonstrating cortical hyperexcitability are technically elegant (Figure 5), although do not provide novel information, as hyperexcitability in HD cortex has been previously reported in diverse mouse models. However, the manuscript currently lacks a direct mechanistic link between cortical hyperexcitability and the vascular dysfunction or astrocytic alterations highlighted in the rest of the manuscript. The integration of cortical hyperexcitability with BBB/BCSFB dysfunction is therefore needed for the coherence of the manuscript. Also, how supplementary video 4 related to this set of data is also not clear. To strengthen the relevance of the results and link them to neuronal excitability, data showing that SEW2871 treatment reverse hyperexcitability in R6/2 mice would be necessary.

4. Differential results in male or female HD patient samples are very interesting. Did authors use both female and male HD mice in the present manuscript? Do the results show sex differences as in HD patient samples? Please provide such information.

Minor Comments

1.In human postmortem analysis, the manuscript states that cortical sections with "well-preserved vessels and meninges" were selected (Page 7, line 7). Clarify potential bias introduced by selecting only samples with intact meninges.
2. The observation of stronger astrocyte activation around arterioles versus venules is intriguing. Consider discussing potential mechanisms, including the possible role of astrocytic TGFβ signaling.
3. Supplementary Video 3. Page 4, line 3: Clarify methodology for identifying perivascular macrophages.
4. Supplementary Video 2,3 and 4: Please provide both WT and HD videos to support data.
5. Paracellular permeability experiments: Clarify whether sequential measurements were performed in the same animals and, if so, specify the time intervals between assays.

Abstract. Line 27, should say “reversed” instead of “rescued”

Version 1:

Reviewer comments:

Reviewer #1

(Remarks to the Author)
The authors have done a very good job addressing the reviewer comments in a constructive and substantive manner. Importantly, they went beyond clarification and added new experimental data (e.g., claudin-5 IHC and expanded human analyses), which strengthens the core conclusions. The revised manuscript is clearer, better contextualised within the existing literature, and more balanced in its interpretation of the findings. While some mechanistic aspects remain necessarily correlative, the authors now appropriately acknowledge these limitations. Overall, the revision significantly improves the quality and impact of the study.

Reviewer #3

(Remarks to the Author)
In the current revised version, the authors have made an effort to improve the manuscript and to reply to most of our points. The work is interesting, methodologically sound for the most part, and extensive.

However, one point remains unsatisfactory. Specifically, concerning the authors’ claim that the use of an open cranial window is the "gold standard" for reaching deeper vessels, we politely disagree. We are aware—from our own experimental work and research focus—of the various imaging preparations used for CNS vasculature and their effects on the tissue. The authors’ "gold standard" argument for the open cranial window is misleading. It is true that a closed cranial window does introduce optical scattering; however, such closed cranial preparations routinely allow imaging at depths well beyond 50 µm —which is consistent with what the cited literature reports—and are widely used to resolve structures such as small as dendritic spines, structures far below the scale of what the authors aim to measure (tracer extravasation). As we clarified in our original comment, an already inflamed model such as R6/2 could be considerably more susceptible to surgical artifacts than a healthy control. Similarly, the reversibility with SEW2871 is not conclusive: it could very well act on surgical inflammation rather than on the underlying pathological process. The use of an open cranial window, in the absence of further confirmation techniques, is therefore a methodological limitation of the study.

We are aware that performing further experiments to corroborate this finding may be challenging. A reasonable proposition would therefore be for the authors to mention this limitation in the text and to suggest the use of closed cranial window preparations or alternative experimental approaches which do not introduce experimental artefacts (e.g., histology) in future work.

Reviewer #4

(Remarks to the Author)
I co-reviewed this manuscript with one of the reviewers who provided the listed reports. This is part of the Nature Communications initiative to facilitate training in peer review and to provide appropriate recognition for Early Career Researchers who co-review manuscripts.

Reviewer #5

(Remarks to the Author)
I would like to thank the authors for their efforts in revising the manuscript and addressing all my comments. All my concerns have been satisfactorily answered, exceeding my expectations. The manuscript is now excellent, and I have no further comments.

Open Access This Peer Review File is licensed under a Creative Commons Attribution 4.0 International License, which permits use, sharing, adaptation, distribution and reproduction in any medium or format, as long as you give appropriate credit to the original author(s) and the source, provide a link to the Creative Commons license, and indicate if changes were made.
In cases where reviewers are anonymous, credit should be given to 'Anonymous Referee' and the source.
The images or other third party material in this Peer Review File are included in the article's Creative Commons license, unless indicated otherwise in a credit line to the material. If material is not included in the article's Creative Commons license and your intended use is not permitted by statutory regulation or exceeds the permitted use, you will need to obtain permission directly from the copyright holder.
To view a copy of this license, visit https://creativecommons.org/licenses/by/4.0/

Dear Reviewers,

We sincerely thank you for careful evaluation of our manuscript, and for thoughtful, constructive, and insightful comments. All responses and changes in the manuscript have been color-coded in green for readability.

Summary of additions and clarifications:

- We **expanded and clarified the rationale for the R6/2 model, in particular, the long-CAG R6/2 line**, supported by a careful literature review and a clear comparison to other HD models used in vascular/BBB research.

- We **reframed and clarified the proteomics interpretation**, explaining what it can and cannot conclude, and how it supports the mechanistic narrative.

- We **added new immunohistochemistry mouse experiments** for claudin-5 that complement and support our in vivo two-photon permeability data.

- We **added new electrophysiology data,** with detailed analysis, showing strong reversal of the barrier phenotype but no rapid rescue of late-stage hyperexcitability.

- We **performed new sex-stratified analyses in mice** for the relevant gliosis readouts and reported these results transparently (with appropriate caution about statistical power).

- We **expanded the human pathology section with quantitative analyses of capillary phenotype**, of GFAP-based vessel/capillary gliosis, to better describe vessel-type patterns and strengthen cross-species alignment.

- **Lastly, we made additional targeted edits** throughout the manuscript (clarified key analysis steps, legends, and included additional figures/videos presentation, supplementary tables, and supplementary discussion where Reviewers noted ambiguity, to make the study easier to evaluate, interpret and reproduce.

Below, we list the detailed, point-by-point response to each Reviewer's comments:

Krzysztof Kucharz and
Martin Lauritzen

on behalf of all authors

REVIEWER #1

Reviewer #1 (Remarks to the Author):
The article "Vascular dysfunction in Huntington's disease is located at the blood-CSF barrier and is rescued by a sphingosine-1-phosphate receptor 1 agonist" by Kucharz et al. presents a highly interesting and technically impressive study of vascular dysfunction in Huntington's disease (HD). The authors primarily use in vivo approaches and include two-photon imaging, electrophysiology, proteomics, and IHC in the R6/2 mouse model, with findings further supported by human IHC staining. The study demonstrates that stimulating S1pr1 with an agonist can rescue some of the observed deficits, notably by suppressing adsorptive-mediated transcytosis (AMT) in arterioles, though not in other vessel types.

We thank Reviewer #1 for his/her appreciation of our work and insightful comments.

Rev1_Comment #1. R6/2 mouse model considerations: The authors used heterozygous R6/2 mice carrying 266–393 CAG repeats, which allows for slower disease progression and longer survival. The study describes the 23-24-week-old mice as representing a late, severe disease stage. However, prior studies (Di Pardo, Amico, Scalabri, et al., 2017)) have reported BBB alterations and tracer leakage consistent with BBB breakdown in late-stage R6/2 mice. Interestingly, this study did not observe significant leakage at the capillary bed, which would typically be expected at such an advanced disease stage. The authors might wish to discuss this discrepancy further.

We thank the Reviewer for this question, which allows us to clarify the primary advance of our work and how it integrates with the existing, sometimes conflicting literature.
Several seminal studies have established that BBB impairment is a feature both in HD patients and in R6/2 mice, evidencing both paracellular and AMT alterations. We have previously shown reduced claudin-5 and occludin and increased endothelial vesicle density in the striatum of HD patients and R6/2 mice, together with DCE-MRI evidence of increased permeability that correlates with disease burden (Drouin-Ouellet et al., 2015). Subsequently, Di Pardo et al. showed that BBB disruption is an early event in R6/2, before overt neurodegeneration (Di Pardo, Amico, Scalabri, et al., 2017).

Yet, key controversies remained. The main issue, excluding the presence of leakage at all, is the debate over the mode of barrier failure. Much of the HD literature inferred paracellular leakage (Di Pardo, Amico, Scalabri, et al., 2017). By contrast, Drouin-Ouellet et al. explicitly described concurrent increased endothelial vesicles in R6/2 striatum, consistent with a mixed paracellular/transcytotic phenotype (Drouin-Ouellet et al., 2015). Opposing both, other work, particularly in non-striatal regions, has reported largely intact cortical BBB structure, with vascular remodeling without breakdown of the barrier (Tan et al., 2025). This leads to uncertainty about whether BBB leakage in HD is widespread or regionally restricted, and whether capillaries or larger vessels (and which barriers: BBB vs BCSFB) are primarily affected. Moreover, classical leakage assays in HD and other CNS diseases rely heavily on bulk tracers such as Evans blue, which tend to bind plasma proteins, provide little, if no information on the transport route, and may also provide false positives (Bolden, Olson, & Cox, 2023). Thus, to understand barrier dysfunction is to understand the means of its failure.

Our study directly addresses these gaps by providing a high-resolution, spatially resolved, and pathway-specific analysis of vascular dysfunction, separating paracellular leakage (small solute diffusion across TJs) from increased vesicular transport (AMT disinhibition), and we localize these processes to defined vascular segments and barrier types. This zonated pattern provides a mechanistic framework that reconciles earlier HD data: the striatal TJ and vesicle alterations reported by Drouin-Ouellet et al. likely reflect a local convergence of both pathways in a region with high metabolic and inflammatory stress (Drouin-Ouellet et al., 2015), whereas the preservation of cortical capillary structure observed in some humans aligns with our finding that capillary BBB can remain structurally intact even when upstream arterioles and pial vessels are dysfunctional (Tan et al., 2025).

Thus, our data move the field beyond the binary concept of an intact versus "leaky" BBB in HD and instead support a model of compartmentalized, mechanism-specific vascular dysfunction-integrating and resolving prior, seemingly divergent, observations and reveal a previously unknown regional variability in HD across the cerebrovascular tree.

We have now highlighted it in the Discussion section in the revised manuscript ***[PAGE 9, PAGE 14].***

If of interest, we also kindly refer the Reviewer to our response to Rev5_Comment #1a and #1b, where we explain the rationale for selecting the long CAG-repeat R6/2 model and provide a model-by-model discussion of HD, with supporting evidence that R6/2 is an appropriate model for BCSFB/BBB studies.

Rev1_Comment #2. Additionally, to strengthen their conclusions and better reconcile these divergent findings, the authors could consider immunostaining for tight junction proteins (e.g., claudin-5, occludin), which could help assess potential structural alterations not detectable by tracer leakage alone. This approach could help clarify whether the absence of capillary leakage reflects a true preservation of BBB integrity or rather a limitation of the detection method used.

We thank the Reviewer for this constructive suggestion. We agree that TJ proteins are fundamental structural components of the paracellular barrier. For instance, our previous work reported reduced expression of claudin-5 in R6/2 mice, consistent with the paracellular leakage (Drouin-Ouellet et al., 2015). To complete our dataset, we have now performed the immunohistochemistry (IHC) in WT, R6/2 and R6/2 treaded mice. Our data shows that R6/2 mice exhibit loss of claudin-5 with the relative reduction being greatest in arterioles (arterioles loose 2x more claudin-5 than capillaries and venules). Treatment increases claudin-5 at TJs selectively in arterioles, with no effect in capillaries and venules (Response Figure 1). This suggests that the reversal of paracellular permeability upon treatment may be arteriole-driven process.

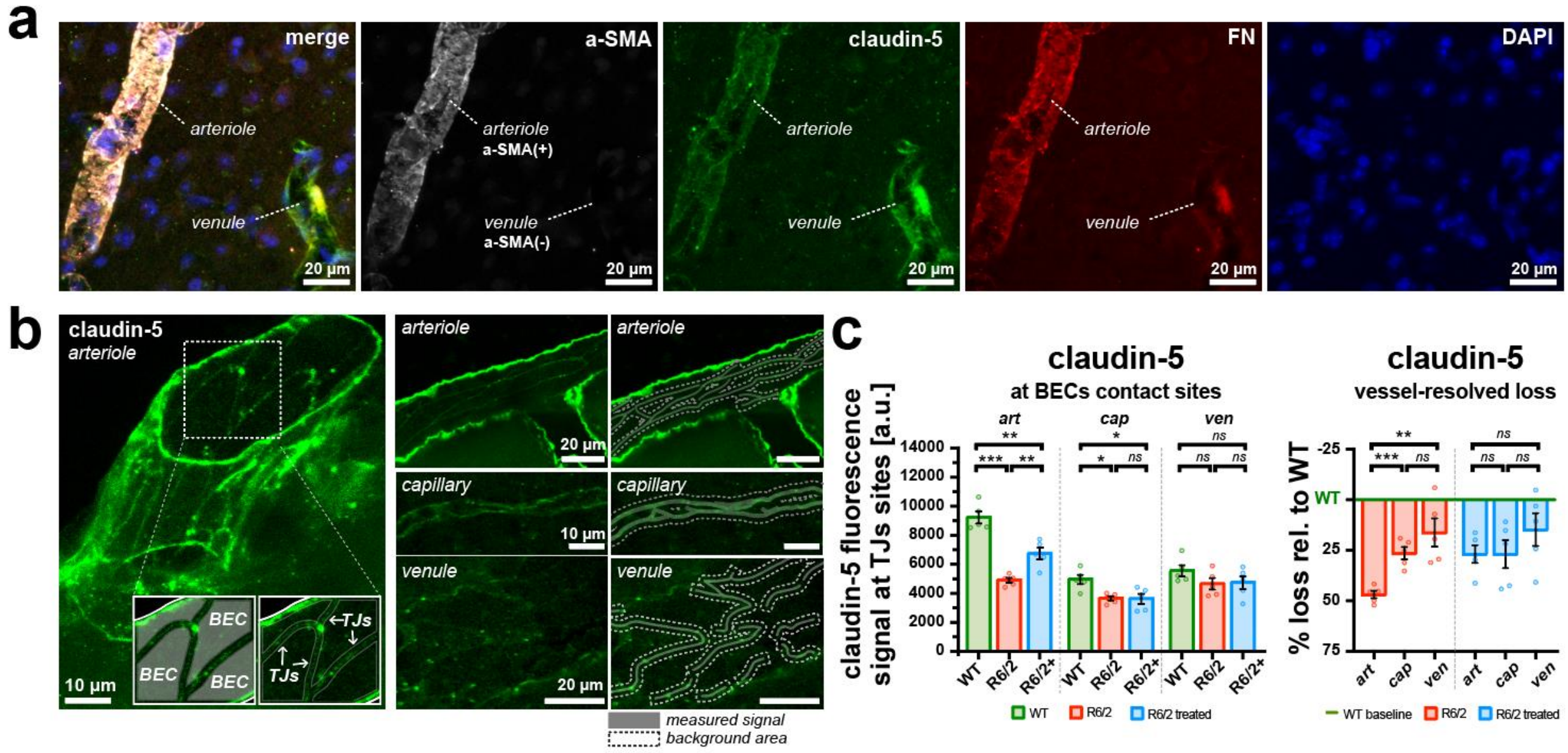

Response Figure 1. Arterioles show the largest reduction in claudin-5 (Cldn-5) in R6/2 mice. **a** Immunohistochemical images of cortical vessels stained for claudin-5. Presence of α-SMA distinguishes arterioles from venules, while fibronectin (FN) outlines the vessels. **b** Left panel: representative image showing the discrete pattern of claudin-5 signal at brain endothelial cell (BEC) contact sites, at the expected location of tight junctions (TJs). Right panel: claudin-5 signal was quantified by subtracting the background signal adjacent to the TJs (dashed lines) from the signal measured at the TJs (shaded gray area). **c** Comparison of claudin-5 signal changes in arterioles (art), capillaries (cap), and venules (ven). Left panel shows the loss of claudin-5 in R6/2 mice, right panel shows that the relative reduction is greatest in arterioles. Treatment increases claudin-5 at TJs selectively in arterioles, with no effect in capillaries and venules. **All panels**: n_{WT}= 5; $n_{R6/2}$= 5, $n_{R6/2treated}$=5. Data is shown as average ± SEM; two-tailed t-test; *=p<0.05; **=*p*<0.01; ***=*p*<0.001; *ns*=non-significant.

However, we respectfully argue that our direct in vivo functional measurements provide a more sensitive, definitive and physiologically relevant assessment than static, post-mortem IHC. NaFluo is one of the more sensitive measures available as it has the lowest molecular weight of any routinely used fluorescent marker (Kucharz, Kutuzov, et al., 2022; Kutuzov, Flyvbjerg, & Lauritzen, 2018; Mathiesen Janiurek et al., 2019).

The central question is not merely whether TJ proteins are present, but whether the barrier is functionally competent. This is because the relationship between TJ protein levels detected by IHC and functional permeability is not always linear. A vessel can exhibit apparently normal staining for claudin-5 ex vivo yet be functionally compromised in vivo due to e.g., mislocalization from the junctional strand [rapid claudin-5 disassembly/redistribution at endothelial junctions after permeability-inducing stimuli (Kondo et al., 2009)], or dynamic regulation (stimulus-dependent internalization and recycling of claudin-5 with concurrent barrier opening/closure (Stamatovic et al., 2009)], that may not be captured by an IHC snapshot. Conversely, a modest reduction in protein expression does not necessarily translate into a functionally significant leak (e.g., size-selective BBB loosening without large-molecule leakage in claudin-5 deficiency; (Nitta et al., 2003); and tissue/bed-dependent preserved basal permeability despite substantial claudin-5 loss (Richards et al., 2022).

*We have now expanded our dataset with claudin-5 assessment in Figure 3, with appropriate descriptions in results **[PAGE 5]** and discussed these new insights in the Discussion section **[PAGE 9].***

Rev1_Comment #3a. Molecular mechanisms and transcytosis: While the work convincingly shows vessel-type-specific dysfunction, the underlying molecular mechanisms remain insufficiently explored. The proteomic analysis offers valuable but correlative rather than causal insights.

We thank the Reviewer for this thoughtful comment. This is a fair point, but our primary aim was to spatially localize and phenotypically define barrier dysfunction, and within this framework, the plasma/CSF proteomics was not meant as a stand-alone causal proof, but as an unbiased systems-level readout constraining the plausible mechanisms underlying observed phenotypes:

***Complement / innate immune axis.** CSF from R6/2 mice showed increased complement C1q subunits and microglial/macrophage markers (e.g., Cd68), alongside chemokines such as Cxcl12. Complement C1q is known to promote endothelial activation, vascular remodeling, and can aggravate brain injury and BBB dysfunction in vivo (Kouser et al., 2015). gC1qR signaling in brain microvascular endothelium also directly modulates permeability, as recently evidenced, e.g., in inflammatory responses post- stroke (Delgardo et al., 2023). Together with elevated chemokines, these changes point to a causal contribution of venular immune cell recruitment and complement-mediated endothelial injury to the paracellular leakage we functionally localize to pial vessels.*

***Vesicle trafficking and AMT machinery.** In parallel, we observe increased Rab8b and related vesicle-trafficking proteins and growth factors such as Vegfc, whereas Rab8a is relatively decreased, in CSF and/or plasma from R6/2 mice. Rab GTPases, including Rab8 family members, are core regulators of polarized membrane trafficking and recycling endosome function, which are central to transcytotic routes in endothelial cells (Garcia-Castillo, Chinnapen, & Lencer, 2017; Villasenor et al., 2019)). Increased Rab8b is therefore mechanistically coherent with the pronounced AMT disinhibition we see in penetrating arterioles, which are transcriptionally enriched for transport and vesicle-trafficking genes in independent datasets.*

***S1P/S1pr1-linked signatures.** Our functional data show that S1pr1 agonism normalizes BCSFB leakage and selectively suppresses AMT in arterioles. Proteomically, SEW2871 treatment partially reverses Rab8b and Plaur upregulation and reduces inflammatory markers such as Cxcl10 and Cd14, consistent with dampened vesicle trafficking and improved junctional integrity at the endothelium. This converges with prior work demonstrating that ApoM-bound S1P and pharmacological S1pr1 stimulation (including*

SEW2871) maintain low paracellular permeability across the BBB and suppress AMT in arterioles (Mathiesen Janiurek et al., 2019) and more generally that S1pr1 signaling enhances endothelial barrier function (Wilkerson & Argraves, 2014).

*We have now clarified this in the Discussion section **[PAGE 11].***

> *"(…) The observation that SEW2871 shifts protein modules that are functionally linked to S1P/S1pr1-dependent control of AMT and paracellular permeability, strengthens the argument that these molecular changes are not generic "late-stage noise" but are mechanistically relevant to the barrier defects (…). However, the definitive causality ultimately requires targeted manipulations of specific candidates, e.g., endothelial-restricted modulation of Rab8b or pharmacological / complement-blocking interventions guided by our proteome signature and cell-type-resolved proteomics or transcriptomics(Garcia-Castillo, Chinnapen, & Lencer, 2017; Villasenor et al., 2019). In this scenario, our findings provide the field with a barrier-relevant proteomic landscape and a mechanistic framework with a prioritized list of molecular pathways for future causal interrogation, and may inform the development of targeted therapeutic strategies in HD, specifically those aimed at barriers and vessels (…)"*

Rev1_Comment #3b. The reported inverse relationship between paracellular leakage and AMT is particularly interesting but mechanistically unexplained.

We appreciate the Reviewer's insight, and we don't have all the answers yet. However, we have added now added text discussing the inverse paracellular leakage - AMT relationship

> *"(…) Here, we used a "slowed-down" R6/2 phenotype provides a stable and sufficiently wide experimental window (23–24 weeks) to perform complex, low-throughput 2PM (see Supplementary Notes). Intriguingly, in our HD model, the most affected arterioles tended to engage one route of barrier failure at a time, i.e., vessels with high paracellular leakage showed only modest vesicular uptake, and vice versa. This mutual exclusivity may reflect underlying vessel-specific regulation and possibly sequential dynamics. Acute stroke studies have shown that BBB disruption unfolds in phases – an initial surge in transcytosis followed by later tight-junction opening, rather than both mechanisms acting concurrently (Knowland et al., 2014). By analogy, in chronic HD, arterioles may be at different stages or under distinct stimuli, some manifesting early AMT-driven dysfunction while others progress to junctional breakdown, yielding the observed inverse correlation. (…)"*

*We have now added this notion to the Discussion section **[PAGE 10].***

Rev1_Comment #3c. Additionally, the study focuses primarily on AMT and paracellular transport through BSA-A488 and NaFluo imaging, respectively, without directly investigating receptor-mediated transcytosis (RMT), which could provide further mechanistic depth.

We appreciate the Reviewer's suggestion that including an RMT component could provide additional mechanistic depth. However, our decision to focus on AMT and paracellular permeability was a deliberate, pragmatic choice given key biological limitations. Unlike AMT and paracellular routes – which can be readily interrogated in vivo with our protocols (e.g., BSA-A488 for AMT, NaFluo for paracellular leakage), currently, there is no broadly applicable in vivo probe for RMT.
This is because each RMT route is defined by a specific receptor–ligand interaction, and "no receptor investigated to date is optimal" as a generic transport tracer. Indeed, roughly a dozen distinct RMT receptors have been identified at the BBB (e.g. the transferrin receptor (TfR), insulin and lipoprotein

receptors, CD98hc), each with its own expression profile and transport kinetics (Haqqani, Belanger, & Stanimirovic, 2024) . Studying RMT comprehensively would thus require parallel investigations of multiple receptor systems – for example, TfR versus CD98hc-mediated uptake, which are known to yield different biodistribution patterns (Khoury et al., 2025; Kucharz et al., 2021; Pornnoppadol et al., 2024) a scope that far exceeds the scale of a single study.

Even if one were to select a single "model" RMT pathway (such as TfR-mediated uptake) as a surrogate, the conclusions would be limited to that receptor alone and the very probe used, and might not generalize across the diverse RMT mechanisms.

We wholeheartedly agree that if robust, generalized RMT tracers or carriers existed, we would use them, but in light of these limitations, we chose to concentrate on transport pathways that could be reliably quantified in vivo, while acknowledging RMT as a critical avenue for future investigation once suitable tools become available.

We have now noted this in the Discussion section ***[PAGE 10].***

> *"(…) Our focus on AMT and paracellular permeability and not RMT was a deliberate and pragmatic choice. Unlike these pathways, RMT lacks a broadly applicable in vivo tracer, as it relies on multiple receptor specific ligand systems with distinct kinetics and biodistribution (Haqqani, Belanger, & Stanimirovic, 2024; Khoury et al., 2025; Kucharz et al., 2021; Pornnoppadol et al., 2024). Studying RMT comprehensively would provide a more holistic view on BBB/BCSFB dysfunction in pathology, and important future direction for HD, and other brain disorders (..)"*

Rev1_Comment #4. Brain region focus: HD pathology predominantly affects the striatum (including the caudate nucleus and putamen). However, the present study mainly examines cortical regions. While this choice might be due to technical considerations related to imaging, exploring vascular pathology in the striatum would improve the study's disease relevance.

We appreciate the Reviewer's concern and fully acknowledge that the striatum is a primary site of neurodegeneration in HD. Its pathological importance is indisputable. However, we are confident that our cortical findings are highly relevant to the broader pathology of HD.

First, the cortical atrophy and cortical thinning occurs early, and often in the preclinical stage of HD alongside or even beyond striatal changes, and its progression has been linked with the extent of the impairment of cognitive, memory and executive function (Hu et al., 2025). This indicates that cortical pathology is not a late by-product of striatal loss, but at the core of HD neurodegeneration (Montoya et al., 2006) . Second, cortical vascular dysfunction may also directly impact cortico-striatal circuits. For example, recent human data show that vascular remodeling in the HD cortex correlates with the extent of striatal degeneration and earlier symptom onset (Tan et al., 2025), suggesting that cortical vascular deficits exacerbate network impairment in HD. Noteworthy, the same study evidenced that extracellular matrix BBB integrity remains intact in the HD cerebral cortex.

Our focus on the cortex was driven by practical considerations of in vivo two-photon microscopy (2PM), which is inherently limited in penetration depth to a few hundred micrometers in the brain tissue. While imaging up to ~250 µm is a reasonable range covering 1/4th of the cortical thickness, it is still less than the location of the striatum (>millimeters below the surface). Imaging striatum would require either cortex resorption or implanting invasive optical probes (e.g. gradient-index microendoscope lenses). While such approaches have been tested for deep brain imaging (Barretto et al., 2011; Jung et al., 2004); the implantation disrupts the tissue and blood vessels via transection of microvessels and compression of neighboring tissue, leading to bleeding and reactive vascular remodeling that can take weeks to stabilize,

all confounding the measures of barriers permeability (Yang & Cui, 2023). Moreover, in contrast to the short imaging span during acute preparations herein, the chronic implanted lenses elicit glial scarring (~50 µm thick), with ongoing astrogliosis in the affected zone (Pernici, Kemp, & Murray, 2019). Such injury responses would further confound the data.

*We have now added these remarks to the Discussion, to both contextualize the broader relevance of our cortical findings **[PAGE 14]** and to clarify imaging considerations **[PAGE 26].***

Rev1_Comment #5. Role of pericytes and VSMCs: Although the article mentions mutant huntingtin (mHtt) aggregates in pericytes and vascular smooth muscle cells (VSMCs) within the microvascular network, the in-depth analysis of barrier dysfunction focuses mainly on endothelial cells and astrocytes. Further exploration of how pericyte or VSMC dysfunction might contribute to the observed vascular pathology would strengthen the study.

We thank the Reviewer for highlighting this important point. Pericytes and VSMCs are integral components of the neurovascular unit (NVU), known to harbor mHtt aggregates in HD, and their dysfunction is suggested to contribute to vascular pathologies (Cheng et al., 2018; Hsiao et al., 2015; Padel et al., 2018). However, in our functional mapping study, linking them to vessel leakage is challenging. First, they are not uniformly distributed, they are structurally heterogeneous, and exhibit distinct, vessel segment-specific roles across arterioles, capillaries and venules (Cai et al., 2018; Hall et al., 2014; Hill et al., 2015; Khennouf et al., 2018; Oztop-Cakmak, Solaroglu, & Gursoy-Ozdemir, 2017). This complexity is further elevated by the absence of precise markers to distinguish pericytes from, e.g., VSMCs in vivo (Hall et al., 2014; Hill et al., 2015; Vanlandewijck et al., 2018b). Mapping each type with its distinct mechanistic contribution to each type of vessel, in physiology, then in disease, is highly challenging and would require pericyte-specific lineage tracing or genetic manipulation. This alone prompts a separate, extensive research program outside the design of the present work.
However, acknowledging their potential contribution to our observations, we have now elaborated:

> *"(…) While paracellular permeability and AMT, are intrinsic to endothelial cells, BECs can be influenced, besides astrocytes, by other cells of NVU. For instance, dysfunctional pericytes could fail to provide the stabilizing signals to endothelial cells, contributing to the disinhibition of AMT (Armulik et al., 2010; Daneman et al., 2010). Hypothetically, pathology in VSMCs could also lead to chronic alterations in blood flow and shear stress, which could secondarily compromise endothelial function (Tarbell, 2010), e.g., via shedding of glycocalyx (Harding et al., 2018) (Kucharz, Mathiesen Janiurek, et al., 2022). Notably, the segments most enriched in VSMCs (arterioles) and pericytes (capillaries) are also those in which we observed the most distinct differences in susceptibility to pathology. This pattern is consistent with segment specific mural cell contributions to BCSFB and BBB maintenance. However, dedicated studies are still needed to determine how the spatial organization and heterogeneity of mural cell populations, collectively or individually, regulate barrier in health and disease. (…)."*

*This note has been now incorporated into the Discussion in the revised manuscript **[PAGE 13].***

Rev1_Comment #6. Human capillary phenotype: The inclusion of human IHC data is a notable strength, but the manuscript does not describe in detail the capillary phenotype or whether distinct patterns of leakage were observed in the human brain samples. Providing these details would improve the translational impact of the findings.

We thank the Reviewer for highlighting the value of our human data and for the excellent suggestion to expand our dataset. We agree that quantitative assessment of human capillary leakage phenotypes would strengthen the translational impact.

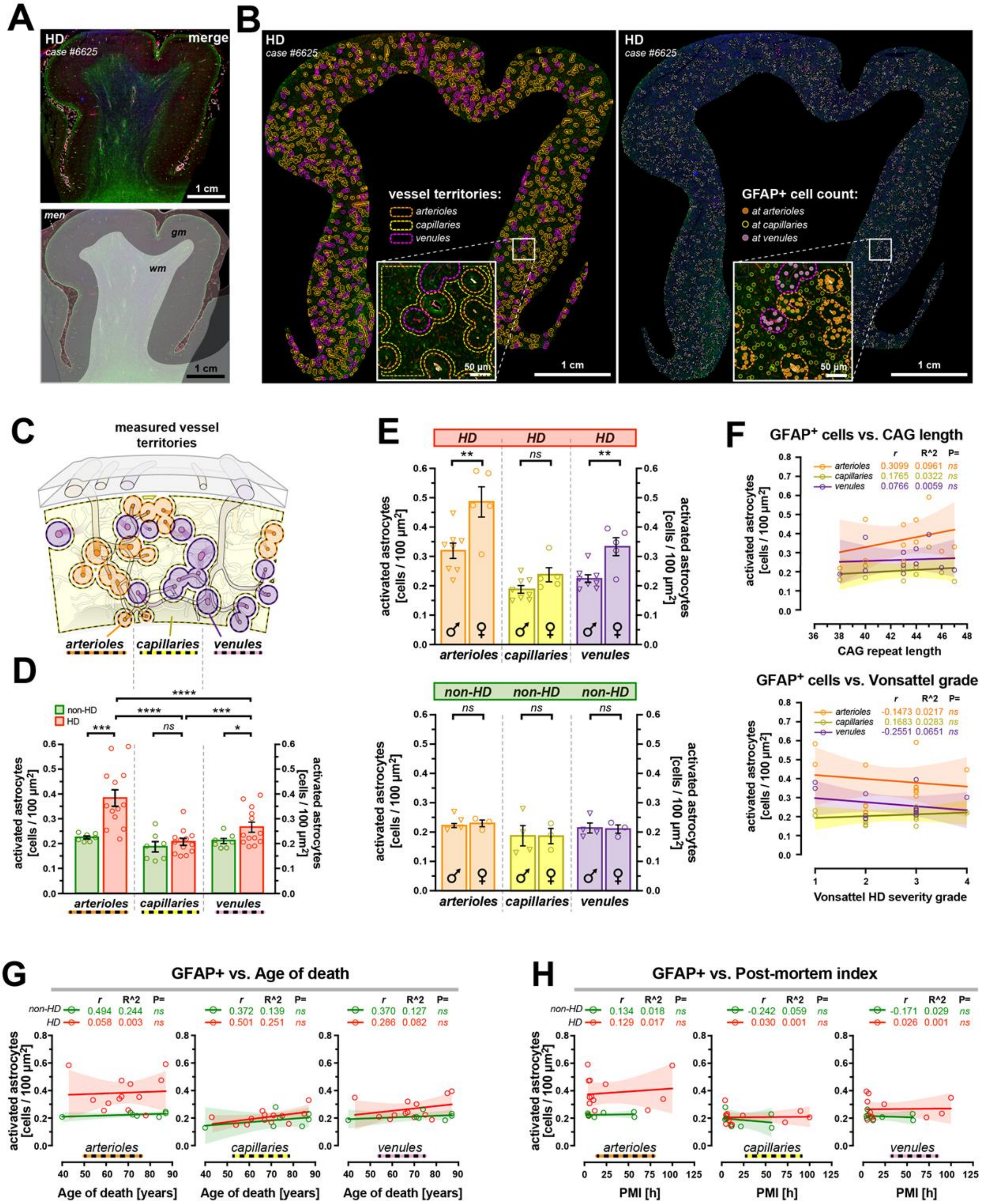

Response Figure 2. Revised quantitative analyses of human *post-mortem* brain sections with inclusion of capillaries. **a** Immunofluorescence image of the cortical region from an HD human brain with a schematic representation of anatomical features. **b** Grey-matter panels corresponding to the region shown in a, with periarteriolar (orange), pericapillary (yellow) and perivenular (purple) territories defined using a 20 µm distance cutoff. GFAP+ astrocytes were quantified within each territory;

cells located in overlapping regions were excluded from both arteriole and venule counts. **c** Schematic drawing of human cortical brain vasculature depicting the location and spatial orientation of analyzed vessel territories. **d** Density of GFAP+ astrocytes in periarteriolar, perivenular and capillary territories in non-HD and HD cases. HD disease phenotype exhibits the enrichment of activated astrocytes at arterioles, with a moderate increase at venules, and the lack of a significant increase at capillaries.
e Sex-stratified analysis of GFAP+ astrocyte density by vessel type. In HD cases, females show higher astrocyte density than males in periarteriolar and perivenular territories, with a trend towards increase in capillary territories. In non-HD cases, GFAP+ astrocyte density is low, similar across vessel types, and with no difference between sexes. **f** Correlation analyses showing that in HD human brains, the density of activated astrocytes is not correlated with donor CAG repeat length or Vonsattel's grade of disease severity. **g-h** Internal controls demonstrating that GFAP+ astrocyte density across all vessel types is unaffected by the donor's age at death (g) or post-mortem tissue interval index (h).
All panels: Each point represents a single donor case, $n_{non\text{-}HD}$= 7 (4 males, 3 females); n_{HD}= 13 cases (8 males, 5 females). The thick lines show a linear fit with a shaded area representing a 90% confidence interval. *r* = Person's correlation coefficient, R^2 = goodness of fit. *p* = the probability that the observed data fits the expected distribution. Data is shown as average ± SEM; two-tailed t-test; *=p<0.05; **=*p*<0.01; ***=*p*<0.001; *ns*=non-significant.

Although, in fixed material, we cannot reliably compare capillary paracellular leakage and AMT to in vivo 2PM imaging paradigm, to complement our arterioles and venules dataset, we have now characterized human capillary phenotype with a robust histopathological analysis of astrocyte activation (GFAP+ cells) (Response Figure 2).
Our data shows that in HD and non-HD donors, capillaries lacked the prominent perivascular reactive astrogliosis defined by intense GFAP+ signal that was evident around arterioles and, to a lesser extent, venules. This is consistent with prior reports of largely preserved cortical BBB-associated features despite vascular remodeling in HD (Lin et al., 2013; Tan et al., 2025), and aligns closely with our functional data in R6/2 mice, where the capillary bed showed no increase in paracellular NaFluo leakage and was the least susceptible segment for AMT upregulation.

Together, this cross-species concordance supports our conclusion that HD exhibits a vessel-type-specific hierarchy of vascular vulnerability.

*We have now incorporated this new dataset into the results section [**PAGE 8**] as well as into **Figure 6** and **Supplementary Figure 8.***

Rev1_Comment #7a. Agonist administration details: The manuscript states that SEW2871 was administered at 10 ug/g for six days, but it does not provide a citation or further methodological details. It would be helpful to include references for this dosing regimen and to discuss whether oral gavage or other administration routes were considered.

We appreciate the Reviewer's request for clarification regarding dosing and route of administration. SEW2871 is a well-established S1PR1 selective agonist in vivo (Jo et al., 2005). Doses at 10 mg per kg have been used to achieve robust receptor engagement and biological effects in mice, including repeated daily intraperitoneal (i.p.) administration paradigms (Sanna et al., 2004). Related work in cerebrovascular disease models further supports daily (i.p. dosing schedules for SEW2871 as a practical approach for sustained, time-controlled receptor stimulation (Ichijo et al., 2015). The six-day duration was chosen as a proof of principle, mechanistic intervention to test short timescale reversibility of barrier dysfunction, based on our previous work (Kucharz, Mathiesen Janiurek, et al., 2022), rather than to model long term therapeutic utility.

Although, we agree that SEW2871 is orally active and has been administered orally in mice in other inflammatory disease contexts (Dong et al., 2014), here we deliberately used i.p. to maximize dosing accuracy and minimize welfare risks in late stage R6/2 mice. At this disease stage, animals commonly show motor impairment and oropharyngeal dysfunctions (Wood et al., 2008), which increases the likelihood of aspiration, variable delivery, and stress with repeated gavage. In contrast, intraperitoneal delivery

provides a controlled and reproducible systemic exposure across animals, which is particularly important for an imaging endpoint that is sensitive to inter individual pharmacokinetic variability.
We have now clarified the text to explicitly state:

> *"We used a 10 mg/kg dose and a six-day intraperitoneal (i.p.) administration paradigm to achieve robust receptor engagement, consistent with prior cerebrovascular disease models supporting daily intraperitoneal SEW2871 dosing as a practical schedule for sustained, time-controlled receptor stimulation (Ichijo et al., 2015; Kucharz, Mathiesen Janiurek, et al., 2022; Sanna et al., 2004)*
> *The drug was prepared in PBS containing 10% DMSO, with vehicle matched injections in control groups. We chose IP injection over oral gavage primarily for reasons of dosing accuracy and animal welfare, because late-stage R6/2 mice often exhibit significant motor impairments and swallowing difficulties, which can make daily oral gavage a stressful procedure and lead to unreliable drug administration. IP injection ensured that each animal received the precise intended dose"*

*This text has been added now to Methods section **[PAGE 17]***

Rev1_Comment #7b. Additionally, SEW2871 is known to have side effects that could influence its long-term utility (see Flemming et al., 2018; PMID: 28538086).

We appreciate the Reviewer's and agree on consideration of adverse effects and long-term limitations. Flemming et al. reported severe cardiac side effects of SEW2871 in a rat model of polymicrobial sepsis, underscoring that systemic context and inflammatory milieu can strongly shape safety and efficacy (Flemming et al., 2018). We explicitly note that:

> *"(…) Here, we used SEW2871 as a short-term pharmacological probe rather than a candidate for chronic treatment. Our experiments involved continuous physiological monitoring during anesthesia and imaging, enabling detection of major systemic instability, and we observed no overt procedural exclusions attributable to the drug under our conditions. However, overt side effects were observed by others in other species, i.e. in rats (Flemming et al., 2018), suggesting that translation would require careful cardiovascular and immunological safety profiling, and prioritization of clinically optimized S1P pathway modulators (…)."*

*This text has now been added to Discussion section **[PAGE 11]***

Rev1_Comment #8. Overall, this is a well-executed and innovative study with significant potential impact on our understanding of vascular dysfunction in HD. Addressing the points above would further enhance the clarity and depth of the work.

We thank Reviewer #1 for their kind words and encouraging, insightful comments.

REVIEWER #2

Reviewer #2 (Remarks to the Author):
I co-reviewed this manuscript with one of the Reviewers who provided the listed reports. This is part of the Nature Communications initiative to facilitate training in peer review and to provide appropriate recognition for Early Career Researchers who co-review manuscripts.

We thank Reviewer #2 for their time, input and effort in reviewing our manuscript.

REVIEWER #3

Reviewer #3 (Remarks to the Author):

Kucharz and colleagues investigate the vascular dysfunction that occurs in the R6/2 mouse model of Huntington's disease (HD). This work builds on previous observations that mutant Huntingtin aggregates in all major components of the vascular unit and this is accompanied by loss of BBB integrity in striatum and putamen, with increased transcytosis and paracellular transport (Drouin-Ouellet et al, Ann Neurol, 2015). The present work focuses on pinpointing more in detail the precise anatomical location and mechanisms of such alterations. More precisely, Kucharz and colleagues demonstrate, via in vivo 2-photon microscopy, that solute leakage is confined to the blood–CSF barrier (BCSFB), at the large sub pial vessels, while capillary permeability remains largely intact. On the other hand, adsorptive mediated transcytosis (AMT), that is normally suppressed in healthy conditions, is largely upregulated across the vascular bed, but to a larger extent in arterioles and penetrating arterioles. Notably, treatment with SEW2871, an agonist of S1P-receptor-1, normalizes BCSFB leakage and dampens arteriolar AMT, but leaves capillaries/venules little changed. Astrocyte activation is congruent with the pattern of vascular alterations, being greater at the pial surface and around arterioles in both animal models and human samples of the disease. The authors also perform a CSF proteome analysis, which shows upregulation in inflammatory and vascular mediators. Interestingly, some of these alterations were reverted by SEW2871 treatment, suggesting that the altered vascular properties can thus be reflected to some extent by the changes in CSF proteome composition. Finally, the authors observe cortical hyperexcitability, and propose that the observed changes in vascular permeability could contribute to some of the electrical alterations seen in the superficial layers of the cortex. Overall, this manuscript offers a detailed in vivo description of HD vasculopathy and pinpoints its alterations to specific vascular segments. Fundamentally, it also provides a potential therapeutic strategy via a druggable target, S1PR1, which has already been tested in neurodegenerative and autoimmune diseases.

We thank Reviewer #3 for their appreciation of our work and insightful comments.

The following points should be addressed before considering publication:

Rev3_Comment #1a. Data interpretation and relevance to disease progression: despite the detailed in vivo characterization, the molecular mechanisms of such vascular alterations remain elusive. Do the authors have a hypothesis for the molecular mechanism(s) underlying vasculopathy in HD?

We thank the Reviewer for this opportunity to elaborate on our mechanistic hypothesis.:

> *"(…) It is plausible that mHtt has direct effect on AMT because huntingtin is an essential integrator of intracellular vesicular trafficking, and mHtt can disrupt Rab-dependent pathways(Caviston & Holzbaur, 2009; del Toro et al., 2009; Hattula & Peranen, 2000). Here, our data suggest the S1PR1 and MFSD2A signaling axis as another central candidate. MFSD2A (Major Facilitator Superfamily Domain containing 2a) is a membrane transporter uniquely expressed by BECs, serving as a key regulator of BBB/BCSFB permeability by suppressing AMT(Ben-Zvi et al., 2014). Mfsd2a KO mice develop a leaky barrier from embryo through adult due to a dramatic increase in AMT despite intact TJs; Supporting a vesicle dominant leak phenotype rather than TJs collapse(Andreone et al., 2017; Ben-Zvi et al., 2014). Recent mechanistic findings directly link Mfsd2a to S1P signaling at the BBB. S1P in the perivascular space binds endothelial S1PR1 receptors to strengthen barrier function, with Mfsd2a being essential for exporting S1P from brain endothelial cells, acting in complex with the S1P transporter(Wang et al., 2020). This maintains an S1P rich microenvironment around the vasculature, necessary for formation and maintenance of the BBB(Wang et al., 2020), whereas in Mfsd2a deficient mice, S1P levels in the endothelial basement membrane and extracellular matrix are greatly reduced, leading to barrier breakdown(Wang et al., 2020). Consistently, deficits in S1P delivery mimic effects of Mfsd2a loss: ApoM, the plasma*

carrier of S1P, is required to present S1P to BBB endothelium and ApoM-/- exhibit increased permeability due to elevated AMT with no change in TJs(Mathiesen Janiurek et al., 2019), very much like Mfsd2a deficient mice. Notably, similar to R6/2 mice, the barrier deficits in ApoM-/- mice can also be reversed by administering an S1P1 agonist, confirming that the phenotype results from S1P signaling deficiency(Mathiesen Janiurek et al., 2019). In addition, similarly to R6/2, ApoM-/- mice arterioles exhibited the highest degree of AMT disinhibition(Mathiesen Janiurek et al., 2019). This arteriolar-specific susceptibility in both Apom-/- and HD mice is further supported by vascular zonation along cerebral vessels, i.e., S1PR1 expression is highest in arterioles axis(Vanlandewijck et al., 2018b), and arteriolar endothelium is specialized for caveolae dependent processes, amplifying the consequences of AMT disinhibition and providing a basis for preferential arteriolar vulnerability(Chow et al., 2020; Mathiesen Janiurek et al., 2019).
Lastly, there is also emerging disease relevance in clinical HD. Single cell dissection of human brain vasculature identified that immune activation co-occurs with concomitant downregulation of MFSD2A alongside other proteins critical for maintenance of BBB/BCSFB integrity in brains of HD patients (Garcia et al., 2022). (…)"

Overall, these independent lines of evidence support an endothelial intrinsic contribution in HD that plausibly converges on S1pr1 signaling to drive the distinct, segment biased barrier phenotypes we reveal herein.

*We have now added this paragraph to the Discussion section in the revised manuscript **[PAGE 9].***

Rev3_Comment #1b. Also, it is unclear whether vascular changes contribute at all to the pathology of HD. Given the very late time-point, it is possible that such alterations are just consequence of the extensive neurodegeneration and do not play a role in disease progression.

*This, indeed, is a fundamental question in the field, not only for HD, but also for other progressive neurodegenerative diseases. We now discuss in the revised manuscript **[Discussion section, PAGE 14]**:*

"(…) While our late-stage experiments do not resolve temporal causality, i.e., whether vascular changes initiate pathology, several observations argue against barriers breakdown as purely end stage, nonspecific consequence of neurodegeneration. Cerebrovascular and blood-brain barrier abnormalities can occur early in HD, including functional and ultrastructural barrier alterations in R6/2 mice and human tissue that precede neuronal loss (Drouin-Ouellet et al., 2015). Physiological imaging in individuals with HD also reports impaired cerebrovascular reactivity, consistent with an early neurovascular component (Chan et al., 2021). More broadly, HD pathology evolves for years before diagnosable onset, leaving ample window for progressive neurovascular contributions (Ross et al., 2014), with MRI assessment of perivascular spaces in premanifest HD suggests impairment of vascular clearance pathways, even when global effects are subtle (Coleman et al., 2024). We propose that the relationship between vascular abnormalities and neurodegeneration is likely a vicious cycle. Early, paracellular leakage at the BCSFB or increased AMT at the BBB allows for chronic exposure of brain parenchyma to neurotoxic and pro-inflammatory blood-derived components (e.g., albumin, complement factors, cytokines). This may create a toxic microenvironment that accelerates neuronal stress, synaptic dysfunction, and ultimately, neurodegeneration. In turn, degenerating neurons and glia can release factors that further compromise vascular integrity, perpetuating the cycle. (…)"

Rev3_Comment #1c. Similarly, it is unclear whether normalizing such changes has any effect on the disease course.

> *To address this, we performed electrophysiological recordings in R6/2 mice that had been treated with the S1PR1 agonist SEW2871. We analyzed R6/2 mice after the same six-day SEW2871 regimen that normalized sub-pial paracellular leakage and reduced AMT selectively in arterioles. Despite this robust vascular rescue, treated R6/2 mice remained electrophysiologically abnormal, showing persistently elevated ΣEPSPs and ΣIPSPs across stimulation frequencies, together with earlier EPSP onset and delayed IPSP onset relative to WT (Response Figure 3). Thus, short-term S1pr1 agonism dissociated two pathological levels: a rapidly reversible barrier phenotype and a late-stage cortical circuit phenotype that remained established at the time of analysis. These data indicate that although S1pr1 engagement retains clear therapeutic value in HD by restoring barrier integrity even at advanced disease stage, it also identifies hyperexcitability as a downstream phenotype less amenable to acute correction.*

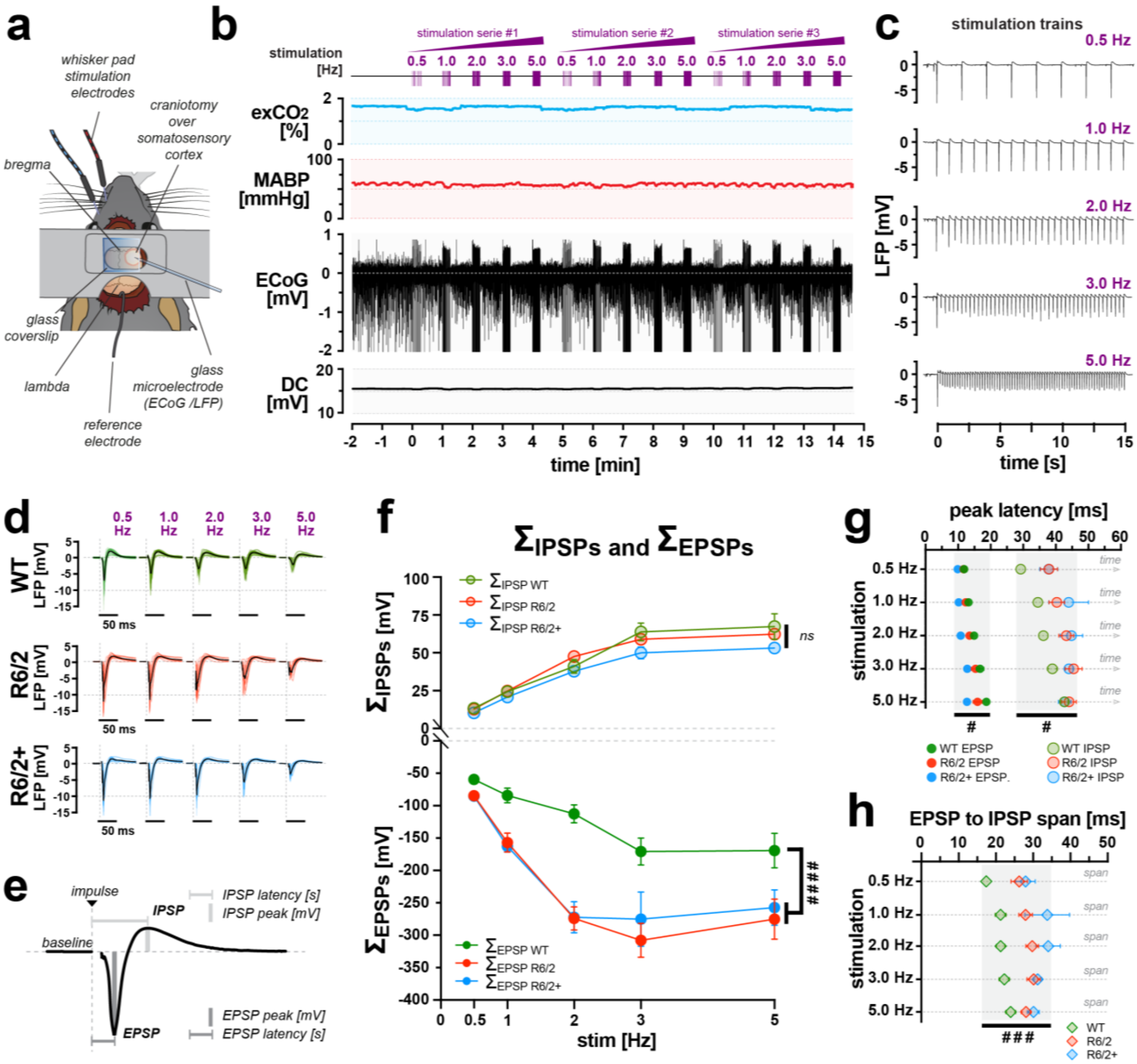

Response Figure 3. Apical barrel cortex hyperexcitability is an entrenched late-stage phenotype resisting barrier restoration. (A) Schematic drawing showing the placement of electrodes for somatosensory stimulation of whisker barrel cortex. **(B)** Examples of concurrent recording of brain electrophysiological activity (electrocorticogram, *ECoG*), direct current *(DC)*, and accompanying systemic parameters, i.e., exhaled CO_2 *($exCO_2$)* and mean arterial blood pressure *(MABP)*. The timeline shows the events of three series of 15 s stimulation trains ranging from 0.5 to

5.0 Hz. **(C)** Changes in local field potential *(LFP)* during trains of stimulations. **(D)** The mean traces of LFP responses (black) for WT, R6/2, and R6/2 treated (R6/2+) mice, with tracers representing individual mice (colored). **(E)** The principal components of LFP response to somatosensory stimulation. Stimulation onset (*impulse)* triggers excitatory post-synaptic potential (*EPSP*), followed by compensating inhibitory post-synaptic potential (*IPSP).* Both EPSP and IPSP were measured for peak (degree of potential change) and peak latency (peak time from stimulation onset). **(F)** Quantitative analyses of the total sum of EPSPs and IPSPs for each stimulation frequency in WT and R6/2 mice. Compared to WT, the R6/2 mice show a significant increase in neuronal excitability (EPSPs) across all stimulation frequencies. Treatment with SEW2871 does not normalize EPSPs and IPSPs in R6/2 mice (R6/2+). **(G-H)** Temporal profiles of synaptic activation showing that both R6/2 and R6/2 mice, regardless of the treatment, have a quicker initiation of excitatory postsynaptic potentials (EPSPs) and a pronounced lag in inhibitory postsynaptic potentials (IPSPs) relative to WT, leading to an extended activation period (EPSP-to-IPSP span).
All panels: n_{WT}=11, $n_{R6/2}$=10, $n_{R6/2+}$=7, mice. Asterisks indicate results from two-tailed t-test; *=p<0.05; **=*p*<0.01; ***=*p*<0.001; ****=p<0.0001; *ns*=non-significant. Hash signs denote the relevant omnibus effect from the two-way mixed-effects model (REML) shown in the graph: $^{\#}$=*p*<0.05; ; $^{\#\#\#}$=*p*<0.001; $^{\#\#\#\#}$=*p*<0.0001. All Data is shown as average ± SEM.

This entrenched state of pathology likely results from the cumulative effects of chronic exposure of neurons to a toxic microenvironment, cell-autonomous effects of mHtt, and/or altered glial function. Critically, this does not argue against potential therapeutic impact earlier in the disease course.

We now explain this in a paragraph added to the manuscript:

> *"(…) In patients, cortical hyperexcitability is a well-established feature of HD, detectable even at early disease stages (Nardone et al., 2007), and sensitivity to glutamatergic excitotoxicity increases with disease progression (Zeron et al., 2002), consistent with a multifactorial origin (Cepeda-Prado et al., 2012; Cepeda et al., 2019; Schippling et al., 2009). Our results show that while the six-day SEW2871 treatment robustly reversed the barrier pathology, it did not normalize cortical hyperexcitability at late disease stage. Because BBB/BCSFB abnormalities emerge early in both R6/2 mice and patients, these findings suggest a plausible early therapeutic window for vascular intervention (Drouin-Ouellet et al., 2015). Consistent with this, sustained, early modulation of the S1P axis in HD models may improve motor performance, extend survival, and preserve synaptic function (Di Pardo et al., 2014; Di Pardo, Amico, Scalabri, et al., 2017; Miguez et al., 2015). In addition, barrier leakage can drive durable circuit remodeling by albumin-driven excitatory synaptogenesis. Blocking these downstream pathways may prevent later hyperexcitability, reinforcing the prevention versus reversal treatment strategies (Bar-Klein et al., 2014; van Vliet et al., 2007; Weissberg et al., 2015) (…)".*

*We have now updated the Results section [**PAGE 7**] and **Figure 5** panels with this new data and highlighted these points in the revised Discussion section [**PAGE 13**].*

*We also kindly refer the Reviewer to the response to **Rev5_Comment #4b**, where we discuss the related mechanistic rationale.*

Rev3_Comment #2. Potential preparation artifact: the paracellular leakage in the pial vessels, although specific to R6/2 model, could be exacerbated by the invasive imaging technique (craniotomy and removal of the dura). Can the authors exclude confirm the observation with a less invasive technique (e.g. histology, thin-skull preparation).

We appreciate the Reviewer's careful consideration of potential experimental artifacts. We selected the open skull craniotomy preparation because it remains the established gold standard for the high resolution, deep tissue two photon imaging required to address our specific questions. Less invasive approaches have important limitations for deep imaging. In particular, thin skull preparations introduce substantial optical scattering from residual bone, which degrades resolution at depths beyond approximately 50 µm (Cramer et al., 2021; Isshiki & Okabe, 2014; Tran & Gordon, 2015). In practice, this would preclude robust quantification and comparison of tracer extravasation in the superficial BCSFB zone

(0 to 60 µm) versus the deeper BBB capillary bed (120 to 180 µm), which is a central within animal comparison in our study.

However, we are confident our findings do not reflect surgical artifact for several convergent reasons. First, in our in vivo two photon assays, the procedure does not measurably disrupt barrier function in healthy operated mice, as blood circulating fluorescent albumin is not detected within perivascular macrophages under baseline conditions, unlike pathological states with disinhibited transcytosis (Mathiesen Janiurek et al., 2019). Second, across our prior work, the same cranial window microsurgery preserves neuronal, astrocytic, pericyte, and smooth muscle morphology and intracellular Ca2+ dynamics, including spontaneous and evoked signals (Fordsmann et al., 2019; Grubb et al., 2020; Khennouf et al., 2018; Kucharz & Lauritzen, 2018; Lind et al., 2013), and we observe intact neurovascular coupling (Cai et al., 2018) (Fordsmann et al., 2019; Hall et al., 2014; Kucharz & Lauritzen, 2018; Kucharz et al., 2017) using two photon imaging, electrophysiology, intrinsic optical imaging, and laser speckle imaging. Third, when we previously left the dura intact, acute versus chronic windows did not alter endothelial transcytosis of nanoparticle drug carriers across the BBB, but those probes are orders of magnitude brighter than albumin and therefore uniquely compatible with chronic window imaging (Kucharz et al., 2021). Finally, the paracellular leakage was specific to R6/2 mice, minimal in identically operated littermates, and fully reversed by SEW2871, which is not expected to occur the leakage was a surgical artifact.

Rev3_Comment #3. Origin of gliosis response: increased vessel permeability and AMT could have an effect also on microglia and on recruited immune cells which, in turn, could drive the increased astrogliosis.

We agree with the Reviewer's insightful comment, and our existing multimodal dataset provides several convergent lines supporting the involvement of recruited immune cells and microglia.

First, our blood and CSF mass spectrometry signature in R6/2 mice is enriched for factors consistent with myeloid engagement and complement activity in CNS-facing compartments, including elevated Cxcl12, Cd68, and C1q in CSF, together with treatment-sensitive immune access markers such as Cd14 and Cxcl10. In the context of barrier compromise, CXCL12 is a key endothelial chemokine that regulates leukocyte positioning and entry at the blood brain barrier, and perturbations of the CXCL12 axis permit parenchymal leukocyte invasion in neuroinflammatory settings, providing a mechanistic bridge from barrier dysfunction to recruited immune cells (Cruz-Orengo et al., 2011; Man et al., 2012; McCandless et al., 2006). Second, our in vivo imaging shows that transcytosed albumin is taken up by perivascular macrophages (cells identity verified in our previous work (Mathiesen Janiurek et al., 2019), demonstrating direct delivery of blood borne cargo to immune competent cells that can amplify local cytokine and complement cascades [Supplementary Video 3;, see also Rev5_Comment #8]. Third, the partial normalization of these CSF inflammatory and vesicular markers with S1pr1 agonism is consistent with reduced immune access when barrier function improves, supporting directionality from barrier failure to immune activation. Lastly, mechanistically, activated microglia can induce reactive astrocyte programs via IL1 alpha, TNF, and C1q, and albumin can further potentiate astrocyte reactivity through TGF beta receptor signaling, aligning well with the Reviewer's proposed sequence (Cacheaux et al., 2009; Ivens et al., 2007; Liddelow et al., 2017).

We have now mentioned it in the Discussion section ***[PAGE 13]***

Rev3_Comment #4. Interpretation of CSF proteome results: the authors attempt to draw parallels between the changes they observe in vascular permeability and modifications in CSF proteome. The link remains elusive. Treatment with the S1PR1 agonist only partially reverts the phenotype. A more balanced description of the results is warranted.

This is a fair comment, and the manuscript have been revised, accordingly, to more clearly describe the CSF proteome as a "molecular signature" of the overall pathological state, rather than a direct, one-to-one readout of vascular permeability.

We also thank the Reviewer, because that led us to add a new thought to the discussion:

> *"(…) CSF proteome in late-stage HD had at least two components. One comprised proteins that arise directly from BBB/BCSFB vascular deficits and are sensitive to S1PR1 agonism (e.g., proteins leaked from blood or released by acutely activated cells). The other likely reflected downstream pathologies, such as advanced neurodegeneration or chronic, self-perpetuating neuroinflammation, that are not reversible by short-term vascular repair. This supports our hypothesis of a therapeutic window [mentioned in Rev3_Comment #1c], and although vascular integrity can be restored, reversing the full spectrum of pathological changes may require earlier or more prolonged intervention (...)".*

*The revised text in Discussion **[PAGE 11]** now incorporates this notion.*

Rev3_Comment #5. Interpretation of the neurophysiology results: changes in electrical excitability cannot be unequivocally ascribed to the vasculopathy (Of note changes in excitability were not checked after S1PR1 agonist treatment). Also for this point a more balanced description of the results is warranted.

We agree with the Reviewer that a direct causal link cannot be unequivocally established from electrophysiological data. We have revised the manuscript to more clearly present the hyperexcitability as a functional neuronal correlate that is consistent with, and likely exacerbated by, the chronic pathological brain environment created by the vascular dysfunction we describe. We now explicitly acknowledge that other factors, including cell-autonomous effects of mHtt on neuronal ion channels and synaptic function, can also contribute to this phenotype.

*We have also now added the following sections to the Discussion **[PAGE 12].***

> *"(…) Although activated astrocytes may locally release neurotrophic factors and antioxidants, they can also acquire neurotoxic properties, secreting reactive oxygen species and excitotoxic metabolites such as quinolinic acid, driving cell damage(Palpagama et al., 2019). This chronic astrogliosis may further compromise essential astrocytic functions involved in the regulation of neurotransmission (Obermeier, Daneman, & Ransohoff, 2013; Palpagama et al., 2019). The extravasated serum albumin binds to TGFβ receptors on astrocytes, triggering signaling cascade (SMAD2 phosphorylation) that results in the downregulation of the potassium channel Kir4.1 and the glutamate transporter EAAT2 (Verhoog et al., 2020). Consistent with this framework, R6/2 mice exhibited increased neuronal excitability, with earlier EPSP onset and delayed IPSP onset.*
> *Although these data do not establish direct causality between barrier dysfunction, astrocyte activation, and altered neurotransmission, there is strong mechanistic precedent that barrier failure can promote hyperexcitability through albumin-driven astrocytic TGFβ signaling, impaired potassium and glutamate homeostasis, and enhanced excitatory synaptogenesis (Cacheaux et al., 2009; Heinemann, Kaufer, & Friedman, 2012; Ivens et al., 2007; Weissberg et al., 2015) (…)"*

We believe that this cautious interpretation provides now a more balanced view on the relationship between vascular and neuronal pathology in late-stage HD.

Rev3_Comment #6. Protein concentration in proteome analysis: The authors find 1638 proteins in plasma and 4746 proteins in CSF. Is this expected, given that average protein concentration in mouse plasma is 60 mg/ml and in CSF 0,3-0,4 mg/ml (PMID 24677814; 23272901)? Also, do the authors perform any albumin removal step?

We thank the Reviewer for this astute technical question, yes this is expected. The Reviewer is correct that the total protein concentration is approximately 100-150-fold lower in CSF compared to plasma. However, we respectfully note that the number of unique proteins identified by mass spectrometry is a function of both instrument sensitivity and sample matrix complexity, not just total protein concentration. The identification depth in discovery proteomics is driven primarily by sample dynamic range and peptide complexity rather than bulk protein mass.

Plasma is dominated by a small set of abundant proteins; albumin alone contributes about half of total plasma proteins and the most abundant 22 proteins account for approximately 99 percent of total plasma protein mass, which restricts sampling of lower abundance species in global mass spectrometry (Geyer et al., 2017; Ignjatovic et al., 2019; Liu et al., 2006). In contrast, CSF has lower total protein but a less extreme abundance distribution and is enriched for central nervous system derived proteins, enabling modern workflows to identify thousands of proteins and many that are not detected in plasma (Cankar et al., 2024; Guldbrandsen et al., 2014).

Regarding albumin removal, we confirm that our workflow did not include immunoaffinity depletion or albumin subtraction in either biofluid. We intentionally avoided depletion because it may also remove non target proteins, including albumin associated species, and can introduce quantitative bias, increase in variability, or loss of candidate biomarkers. (Gunther et al., 2015; Tu et al., 2010; Yocum et al., 2005).

We have now added an explicit Methods clarification with this cautionary note, stating that samples were analyzed without depletion using direct digestion, supporting deep coverage without depletion ***[PAGE 20].***

Rev3_Comment #7. Minor points: The GFAP activation pattern seems to have a sex bias in human samples. Did the authors observe also sex differences in the animal model?

We included both male and female mice in the study (per genotype: 2 males and 3 females; total n = 5). In response to the Reviewer's comment, we re-analyzed the GFAP activation pattern after stratifying the mouse data by sex. This analysis did not reveal an overt sex-dependent divergence in the mouse cohorts. Instead, the same overall pattern was preserved in both males and females, namely a preferential increase in GFAP+ astrocytes in periarteriolar, but not perivenular, territories in R6/2 mice (Response Figure 4). However, this result should be treated with caution, because of the low statistical power. We therefore considered pooling across sexes to be the most statistically responsible representation of the mouse dataset for the primary analysis.

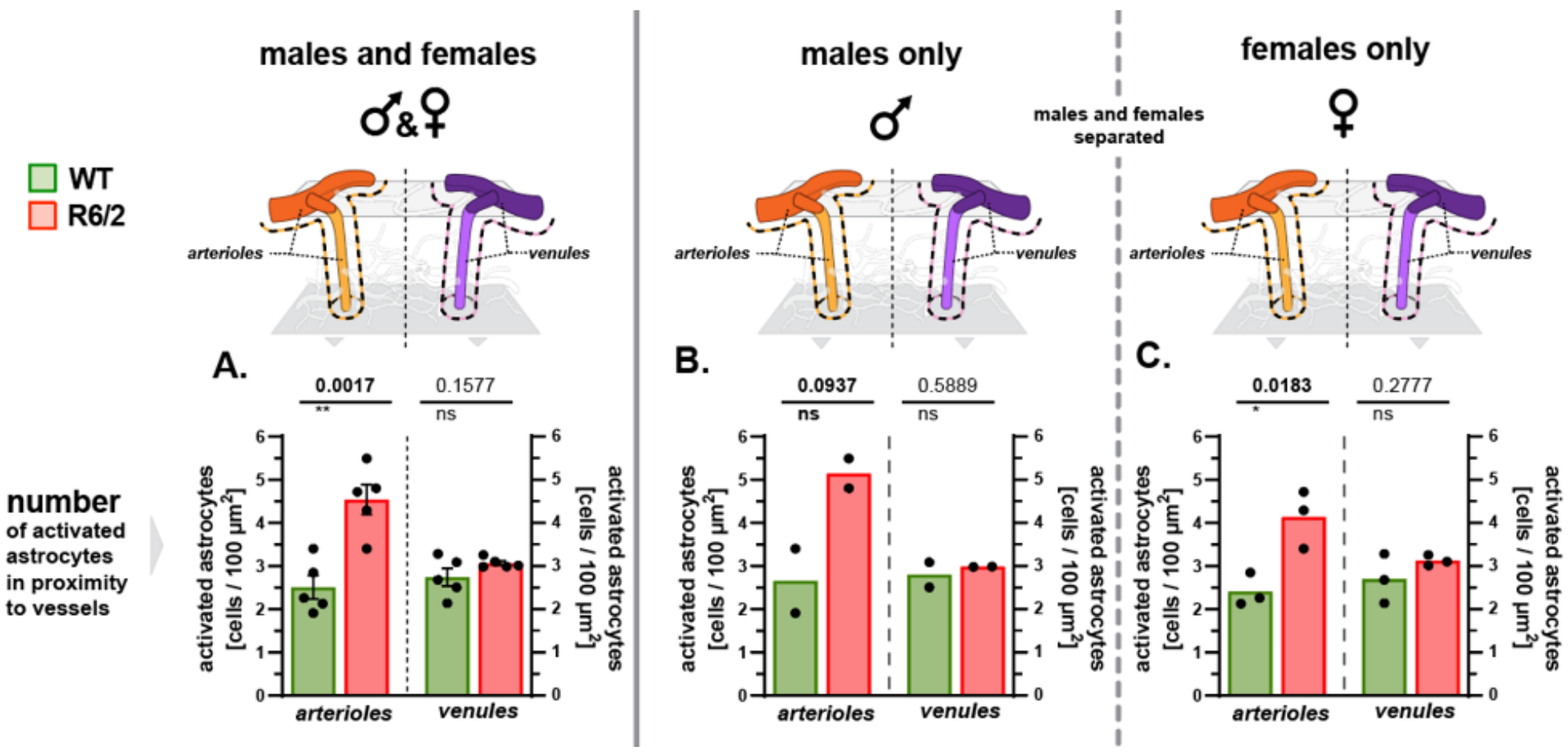

Response Figure 4. No overt sex-specific differences in astrocyte activation patterns between WT and R6/2 mice. Upper panels, schematics showing the periarteriolar and perivenular territories used for quantification. Lower panels, quantification of activated astrocytes in **(A)** pooled data and separately in **(B)** males and **(C)** females.

Irrespective of sex, GFAP+ astrocytes showed a tendency toward preferential increase around arterioles. All panels: two-tailed t-test; *P<0.05, **P<0.01, ***P<0.001; ns, not significant; nWT = 5 and nR6/2 = 5 mice (2 males and 3 females per genotype). Data are shown as mean ± SEM, except in panels B and C, where SEM and SD are not shown because population distribution estimates are not reliable at this sample size.

Still, the lack of overt sex effect in mice vs. robust effect in human may reflect the influence of hormonal cycles or the more complex genetic and environmental backgrounds in humans. This apparent discrepancy is not unexpected in HD, where sex effects may be reflected in clinical endpoints. Large clinical cohorts report sex related differences in specific symptom trajectories, but the direction and magnitude vary across measures and studies. (Zielonka et al., 2013; Zielonka & Stawinska-Witoszynska, 2020). Similarly, R6/2 HD transgenic mouse model can show wide range of sex-dependent phenotypes (Wood et al., 2010). However, to our knowledge, sex-stratified analyses of GFAP+ astrocyte activation in R6/2 mice have not been yet reported in the literature.

*Nonetheless, we have now clarified that we used pooled group of both sexes for quantitative immunohistochemistry analyses after ad hoc analyses revealed no overt differences between males and females **[PAGE 25]**.*

Rev3_Comment #8. The legends sometimes describe results and sometimes describe the figure (e.g. figure 4B vs figure 4D). Please choose a uniform style.

We thank the Reviewer for pointing this out. We have now reviewed all figure legends and have revised them to ensure a consistent and uniform style throughout the manuscript and to keep the same cadence across panels.

Rev3_Comment #9. Figure 3: please correct the legend for figure 3E and 3F

We thank the Reviewer. We have reviewed and corrected the legend for Figure 3 to ensure accuracy and clarity regarding panels for the two analyzed biofluids.

Rev3_Comment #10. Figure 4: please correct the legend for figure 4B and 4C.

We thank the Reviewer. We have reviewed and corrected the legend for Figure 4 to ensure accuracy and clarity.

Rev3_Comment #11. Extended data figure 2: please correct the typo in line 35 (show demonstrate)

We thank the Reviewer for catching this typo, and it has now been corrected.

Rev3_Comment #12. Video 4: constrictions are hard to catch for the non-expert and no comparison with control is provided. Quantification of the data would make the observation more relevant.

We appreciate this feedback. The purpose of Supplementary Video 4 was to provide a qualitative illustration of the vascular dynamics. Importantly, such constrictions can occur within seconds, whereas our volumetric scans were acquired every 7.5 min because the imaging paradigm was optimized for high spatial resolution across large cortical volumes and for AMT assessment, rather than for capturing rapid diameter fluctuations. Under these conditions, quantitative analysis of constriction frequency or kinetics would not be reliable. A rigorous quantification would therefore require a separate study with dedicated

methods optimized for fast vascular events across vessels of different diameters. We have therefore kept Video 4 as a qualitative observation considering it as an interesting direction for future investigations.

To improve clarity, we added annotations to the video to highlight the moments of arteriolar constriction and included a corresponding video from a WT animal for comparison, with time-resolved colormap of changes for both for easier evaluation.

REVIEWER #4

Reviewer #4 (Remarks to the Author):
I co-reviewed this manuscript with one of the Reviewers who provided the listed reports. This is part of the Nature Communications initiative to facilitate training in peer review and to provide appropriate recognition for Early Career Researchers who co-review manuscripts.

We thank Reviewer #4 for their time, input and effort in reviewing our manuscript.

REVIEWER #5

Reviewer #5 (Remarks to the Author):

The manuscript by Kucharz et al. it is very well written and illustrated, and provides an elegant and thorough characterization of heterogeneous vascular alterations in the Huntington's disease (HD) R6/2 mouse model and in human postmortem samples. The authors demonstrate significant blood–brain barrier (BBB) and blood–cerebrospinal fluid barrier (BCSFB) dysfunction and further show that treatment with a sphingosine-1-phosphate receptor 1 (S1PR1) agonist, SEW2871, can reverse some of these alterations. These findings highlight potential therapeutic avenues for HD and represent a valuable contribution to the field. However, major revisions are required to strengthen the validity of the model and clarify mechanistic links. In particular, the choice and characterization of the HD model, the interpretation of the S1PR1-related effects, and the integration of some experimental data require further clarification and additional evidence. Major Comments:

We thank Reviewer #5 for their appreciation of our work and insightful comments.

Rev5_Comment #1a: The R6/2 mouse model used in the study carries >200 CAG repeats, which is significantly higher than the original R6/2 line and may alter the expected HD phenotype. Please provide supporting data or literature demonstrating that these mice still exhibit a robust and relevant HD phenotype (…)

We appreciate the Reviewer's insightful comment regarding the specific R6/2 line used in our study. While it is true that this line carries a higher repeat number than the original R6/2 colony, this does not invalidate the model but rather refines its utility.
Our use of an R6/2 line, in addition, with an expanded CAG repeat range (266–393) was a deliberate, strategic choice designed to balance the robust pathology required for mechanistic study, and make it feasible to perform two-photon imaging in vivo across three experimental groups in otherwise rapidly progressing disease

We have now prepared a separate, detailed discussion paragraph in Supplementary material, where we explicitly explain the rationale of using longer repeat length, the preservation of core R6/2 phenotype and imaging considerations ***[Supplementary Note, PAGE 15].***

Rationale for Using the Long-CAG variant of R6/2 model of HD

"(…) A key benefit of using the high-CAG R6/2 line is its prolonged disease course. There exists a well-documented, paradoxical relationship between CAG length and disease progression. R6/2 mice with extremely expanded CAG tracts (>300) have a slower progression and extended lifespan compared to the conventional ~150 CAG R6/2 mice (Tang et al., 2011) (Cummings et al., 2012). This phenomenon is well documented: increasing the R6/2 CAG from ~110 to 150 accelerates disease, but further expansion to ~210–310 ameliorates severity and delays onset (Cummings et al., 2012). For instance, Tang et al. reported that R6/2 mice carrying ~300 CAGs live longer and develop symptoms more gradually than the ~150 CAG line (Tang et al., 2011). This allowed us to perform technically demanding in vivo two-photon imaging at advanced disease stages, which would be infeasible in standard R6/2 mice that typically reach endpoint by ~12-14 weeks.

Despite the extended timeline, the high-CAG R6/2 model retains a robust HD-like phenotype, encompassing the hallmark neuropathological and behavioral deficits of HD. Like conventional R6/2 mice, the >300 CAG lines show progressive brain atrophy (striatum and cortex), weight loss, and motor dysfunction (Etxeberria-Rekalde et al., 2020). Notably, significant cortical and striatal neurodegeneration with gliosis and huntingtin aggregation (Etxeberria-Rekalde et al., 2020), classic features of HD, are still present in the long-CAG line, albeit on a delayed schedule. For example, Tang et al. observed that high-CAG R6/2 mice form mutant huntingtin (mHTT) inclusions and transcriptional changes similar to ~150 CAG line, but with later onset (Tang et al., 2011). Interestingly, the ultra-long CAG expansions can alter the subcellular pathology, i.e., R6/2^{Q300} mice tend to accumulate more small cytoplasmic aggregates (versus large nuclear inclusions in R6/2^{Q150}) (Tang et al., 2011), yet they ultimately develop the same pathogenic endpoints (synaptic dysfunction, neuronal loss). Importantly, key HD pathological hallmarks are robustly expressed in this model. By 14–16 weeks, long-CAG R6/2 mice exhibit: (i) neuroinflammation and gliosis: increased GFAP^{+} astrocytes and activated microglia paralleling early neuronal injury (Etxeberria-Rekalde et al., 2020); (ii) pronounced cortical atrophy and neuron loss, akin to human HD where cortex and striatum undergo progressive degeneration (Southwell et al., 2015) (Etxeberria-Rekalde et al., 2020); (iii) synaptic and electrophysiological dysfunction, including cortical hyperexcitability and impaired corticostriatal transmission, as shown in R6/2 lines of various CAG lengths (Barry et al., 2022; Cummings et al., 2012); and (iv) mHTT aggregation and ubiquitination throughout the brain (Etxeberria-Rekalde et al., 2020)). These outcomes reinforce that the long-CAG R6/2 mice still manifest a robust and relevant HD phenotype.

Notably, the "slowed-down" R6/2 phenotype provided also a more stable and sufficiently wide experimental window (~2.5 weeks) to perform complex two-photon imaging. This technique has low throughput, yielding one animal imaging session per day. Using slow-progressing high-CAG R6/2 is a feat that allows imaging that would be technically unfeasible in more aggressive lines where mice undergo rapid decline and imminent mortality. This extended trajectory enabled the experiments to be conducted across all experimental groups over ~2 weeks without confounding disease stage, i.e., in each group and across the groups, the first imaged mouse was not systematically healthier than the last. As a result, we could treat the entire cohort as being at approximately the same disease timepoint, and perform comparisons between the groups (WT vs. R6/2 vs. R6/2 treated mice)(…)"

In short, the long-CAG R6/2 mice provided a larger window to investigate barriers dysfunction in late-stage HD, a central goal of our study. This choice balanced severity with survivability and feasibility of model for imaging, providing the "best of both worlds" scenario: preserving the robust vascular phenotype of the R6/2 line while offering the temporal resolution necessary for advanced imaging. Crucially, the pathology observed in our long-CAG R6/2 line remains pathophysiologically relevant to HD, as detailed above.

We explicitly reference the reader to this Supplementary Note it in the Methods ***[****animal model:* ***PAGE 15;*** *methodological considerations:* ***[PAGE 26],*** *Results* ***[PAGE 3]*** *and Discussion [****PAGE 10****] of the revised manuscript.*

Rev5_Comment #1b: (…) or validate results with a more appropriate model to ensure validity of the results.

While we acknowledge that no single model perfectly recapitulates every aspect of human HD, the current evidence indicates that R6/2 mice uniquely fulfill the criteria of exhibiting HD-like vascular deficits (including cortical BBB disruption) ***(See Response Table 1).***

Model	Cortical Pathology<br>(Atrophy, Gliosis, Synaptic Loss)	BCSFB/BBB Disruption
**R6/2**<br>*(Exon-1)*	Severe, early-onset; robust atrophy and gliosis (Etxeberria-Rekalde et al., 2020).	**YES**. Significant leakage and AMT disinhibition (Di Pardo, Amico, Scalabri, et al., 2017).
**YAC128**<br>*(Full)*	Late-onset; atrophy visible by 9–12 months (Slow et al., 2003).	**NO**. BBB is intact under normal conditions, but can be breached by systemic LPS administration (Franciosi et al., 2012).
**BACHD**<br>*(Full)*	Gradual; significant atrophy only after 12 months (Spampanato et al., 2008).	**NO.** Integrity maintained throughout life, normal cerebrovascular function (Mantovani et al., 2016).
**Knock-in**<br>*(Q140/150/Q175)*	Typically, very slow; subtle changes until 12–22 months (Bayram-Weston et al., 2012; Smith et al., 2014).	**NO(?)**. No reported leakage. Due to late onset age, possibility of interference of aging processes on BCSFB/BBB for baseline recordings (Yang et al., 2020).
**Human HD**<br>*(ground truth)*	Hallmark cortical thinning and neurodegeneration.	**YES.** Documented leakage and vascular changes (Drouin-Ouellet et al., 2015).

Response Table 1. Comparison of R6/2 s with YAC128, BACHD, knock-in, and human HD in terms of cortical and BBB dysfunction.

We thank the Reviewer for prompting this detailed comparison. We recognize that this is important information that warrants extended discussion, and has now been implemented in the manuscript in the form of table above AND supplementary material/discussion section, where we explicitly explain the rationale of choosing R6/2 over other models ***[Supplementary Notes, PAGE 14****].*

"(…) Consideration of Alternative HD Models

The R6/2 model of HD is the only HD mouse model in which BBB defects have been consistently demonstrated in contrast to BCSFB/BBB HD phenotypes absent in other HD mouse models (YAC128, BACHD, knock-in). Di Pardo et al. showed that R6/2 mice develop significant BBB leakage at symptom onset (~6–8 weeks), far earlier than previously assumed, starting at early disease (6 weeks), and worsening by the late stage (12 weeks) (Di Pardo, Amico, Scalabri, et al., 2017)). This indicates that the breach of BBB integrity, preceding or coincident with motor symptom onset in R6/2 mice (Di Pardo, Amico, Scalabri, et al., 2017)). Our current data agree with this: we showed ~4-fold higher paracellular tracer leakage in cortical microvessels (particularly surface vessels) in R6/2 compared to WT controls. Notably, the R6/2 model reproduces the BBB breakdown seen in HD, and does so in a spatially relevant manner (affecting cortex and striatum, the same regions as in HD patients) (Drouin-Ouellet et al., 2015))

Although distinct HD models have their merits, we concluded that none would as readily allow us to interrogate late-stage cortical BBB pathology as R6/2, because:

1) ***YAC128*** *(full-length human HTT, ~128 CAG) model reproduces many HD features over a prolonged timescale (12–18 months). However, BBB disruption is minimal in YAC128, especially at early stages. In baseline conditions, young YAC128 mice show no significant tracer leakage; even at 12 months, only subtle vascular changes are noted (e.g. slightly*

increased microvessels density and reduced diameter) with intact barrier function (Franciosi et al., 2012). These mice, require an external insult (chronic LPS inflammation) to unmask BBB leakage, –LPS triggered striatal fibrinogen extravasation and vessel wall thickening, but untreated YAC128 mice did not exhibit increased BBB permeability (Franciosi et al., 2012). Moreover, to reach a comparable disease stage to R6/2, YAC128 mice would need to be ~12–15 months old, which presents practical challenges for two-photon imaging.

2) ***BACHD*** *(full-length HTT BAC, ~97 CAG): BACHD mice express mutant huntingtin ubiquitously and show progressive motor impairments starting at ~3 months (Mantovani et al., 2016). However, their neuropathology is notably mild through mid-life, and BACHD mice have normal striatal neuron counts at 12–15 months, with no signs of BBB degeneration, even in 12-month-old mice. Given the absence of overt neurodegeneration or inflammatory pathology at the ages typically studied, the BBB in BACHD is presumed to intact (Mantovani et al., 2016), with authors explicitly cautioning against the use of this model for preclinical studies in HD.*
3) ***Knock-in models (Hdh^Q140, Hdh^Q150, zQ175, etc.)****: Knock-in (KI) mice have the mutant CAG expanded in the native mouse Htt gene, preserving endogenous regulation. They are genetically precise but slow-progressing, and to our knowledge, there is no evidence of spontaneous BBB leakage in Q140 or Q175 mice even at 1+ year of age, consistent with their milder HD pathology (Menalled, 2005; Smith et al., 2014). As such, detecting and imaging BBB dysfunction in them would be impractical, having to age cohorts to 18–24 months, and even then, the changes might be too subtle. At the same time, the late onset age would not only dramatically increase the risk of mortality related surgical procedures and imaging anesthesia, but also be confounded by presence of deleterious aging processes that gradually compromise BCSFB/BBB (Yang et al., 2020). Therefore, using KI mice would not meet our experimental need for a robust, measurable BBB phenotype in a reasonable timeframe.*

Beyond gross leakage, R6/2 mice exhibit a rich spectrum of neurovascular pathology that closely mirrors human HD. For example, R6/2 brains have aberrant microvascular remodeling – increased vessel density but reduced vessel diameter – suggestive of pro-angiogenic yet maladaptive changes (Drouin-Ouellet et al., 2015) and mHTT aggregates deposit in blood vessels and perivascular cells of R6/2 mice, as in HD patients (Drouin-Ouellet et al., 2015)). Notably, we have previously found mHTT in all major cellular components of the neurovascular unit accompanied by substantial BBB leakage in the striatum changes (Drouin-Ouellet et al., 2015), and confirmed similar vascular and BBB changes in human HD post-mortem brains, firmly linking the R6/2 vascular phenotype to the human condition changes (Drouin-Ouellet et al., 2015).

Overall, alternative models either lack substantial cortical BBB disruption (YAC128, BACHD, KI) or require very long durations to manifest any BBB phenotype. By contrast, the R6/2 model – including our high-CAG variant, exhibits early, robust BBB dysfunction, closely paralleling human HD (…)"

We now explicitly point the reader to this information in the Methods ***[PAGE 15]****, Results* ***[PAGE 3]****, and Discussion section* ***[PAGE 10]*** *in the revised manuscript.*

Rev5_Comment #2a: S1PR1 is expressed not only in brain endothelial cells (BECs) but also in other neural and non-neural cell types. Therefore, systemic S1PR1 agonism cannot be attributed exclusively to effects on BECs. Please expand the discussion to clarify potential off-target effects and the contribution of other S1PR1-expressing cells. Also, please discuss the rationale for selecting SEW2871 drug and its dose, its selectivity for S1PR1 receptor and whether other S1P receptor subtypes may be involved. How is S1PR1 expression in HD brains? Please also discuss whether additional drug effects on other brain cells could contribute to the observed results.

We appreciate the Reviewer's insightful comment and fully acknowledge that S1PR1 is expressed on multiple CNS cell types, and in peripheral tissue, but so far, the evidence points to the direct and key involvement of endothelial S1PR1s in the failure and rescue of the barriers function. Specifically:

"(…) Although S1PR1 is not endothelial-specific, the rescue pattern observed here is most consistent with a predominantly endothelial action of SEW2871. S1pr1 is abundant in endothelial cells, especially enriched in arterioles, with markedly lower expression in neurons and astrocytes, and minimal expression in microglia (Garcia et al., 2022; Li et al., 2025; Nitzsche et al., 2021; Vanlandewijck et al., 2018a; Yang et al., 2023). This distribution supports the point that a systemically accessible agonist would be expected to exert prominent effects at the endothelial barrier, particularly in arterioles. Astrocytes do express S1PR1, and astrocytic signaling can shape the BBB function in disease contexts (Yang et al., 2023). Neuronal S1PR1 can also modulate intrinsic properties and excitability in a circuit and cell type-dependent manner (Mork et al., 2022). Systemically, S1pr1 agonism can also reduce inflammation by promoting anti-inflammatory macrophage phenotypes and restraining lymphopoiesis (Blaho et al., 2015; Sanchez, 2016; Sun et al., 2024), benefiting barriers strength. Here, the SEW2871 effect on claudin-5 expression and AMT was largely confined to arterioles with no detectable rescue in capillaries or venules. If SEW2871 was acting primarily via general immunosuppression, one might expect a more uniform and widespread rescue across all vessel types. In addition, our observation is supported by previous findings in ApoM KO mice, where endothelial S1PR1 signaling deficits produce a barrier phenotype dominated by increased AMT in arterioles (Mathiesen Janiurek et al., 2019), closely mirroring HD phenotype in our study. While we cannot exclude a secondary, beneficial effect on S1PR1 stimulation of other cell types, in the context of barriers rescue, the most plausible model appears to be direct action on compromised endothelium. Noteworthy, while S1P reduced levels has been observed in animal models, including R6/2 mice (Di Pardo, Amico, Basit, et al., 2017; Pirhaji et al., 2016), the transcript levels of S1PR1 appear to be unchanged in HD patients (Garcia et al., 2022), suggesting that rather than S1PR1 downregulation, a reduced blood-borne S1P levels that may play role in impaired S1PR1 signaling.(…)"

*We have now expanded the Discussion section with the paragraph above **[PAGE 12]***

Rev5_Comment #2b (following on specific excerpt from Rev5_Comment #2a): "(…) Also, please discuss the rationale for selecting SEW2871 drug and its dose, its selectivity for S1PR1 receptor and whether other S1P receptor subtypes may be involved (…)"

We thank the Reviewer and agree that drug choice and selectivity are central for interpretation and requires more detailed rationale.

We now explain:

"(…) We chose SEW2871 because it is a high affinity S1PR1 selective agonist developed to recapitulate S1PR1 driven biology (Jo et al., 2005)], without the broader subtype engagement of e.g. fingolimod, which after phosphorylation acts on S1PR1, S1PR3, S1PR4, and S1PR5 and functionally antagonizes S1PR1 through internalization and downregulation (Hannun & Obeid, 2008; Toman & Spiegel, 2002)]. Mechanistically, this matters for our study because across cell types and receptor subtypes; a S1PR1 selective agonist SEW2871 provides a cleaner pharmacological probe than a multi subtype modulator. (…)"

Regarding the dose and treatment timepoints, we kindly refer the Reviewer to the response to Reviever1_Comment7a, where we explain in details the rationale of our approach. Briefly, our regimen, 10 mg per kg daily i.p. injections for six days was chosen to achieve robust receptor engagement in vivo in line with prior works, maximizing dosing accuracy, while minimizing welfare risks associated with oral gavage in late stage R6/2 mice.

All information has now been implemented into the Methods section ***[PAGE 16]***

Rev5_Comment #3a: The experiments demonstrating cortical hyperexcitability are technically elegant (Figure 5), although do not provide novel information, as hyperexcitability in HD cortex has been previously reported in diverse mouse models.

We acknowledge that cortical hyperexcitability is not the manuscript's primary focus, but the sensory-evoked multichannel LFP data (Figure 5) remain important because they co-localize with vascular pathology in the same cortical regions. Although they resolve the exact mechanism, we respectfully defend the inclusion of these data on methodological and conceptual grounds.

Using in vivo local field potentials (LFPs) recordings during sensory stimulation allowed far higher spatial and laminar resolution than prior studies. Our approach yields precise timing of synaptic events, which enabled us to quantify dissociation between excitatory (EPSP) and inhibitory (IPSP) timing and to detect layer-specific response differences, previously unknown in HD. By contrast, earlier reports described cortical hyperactivity or network changes more globally, without dissecting stimulus-driven EPSP/IPSP dynamics or laminar specificity (Jiang et al., 2016; Walker et al., 2008), or were limited to in vitro model systems (Virlogeux et al., 2018). Our detailed analysis of evoked LFPs under sensory drive is therefore novel and provides a finer-grained quantification of cortical excitability than prior in vivo studies (Cepeda et al., 2010).
Thus, even if not central to the main conclusions, Figure 5's findings complement the manuscript's themes by reinforcing a common pathological focus. In summary, the laminar LFP results, although not tied to a single mechanism, strengthen the paper by providing high-resolution evidence of altered cortical excitability that is consistent with and spatially coincident with the vascular and astrocyte changes we report.

In addition, we have now incorporated new paragraph to discussion section, where we also describe our electrophysiological findings in the broad context of a therapeutic window (we kindly refer the Reviewer to the response Rev3_Comment #1c.).

Rev5_Comment #3b: However, the manuscript currently lacks a direct mechanistic link between cortical hyperexcitability and the vascular dysfunction or astrocytic alterations highlighted in the rest of the manuscript. The integration of cortical hyperexcitability with BBB/BCSFB dysfunction is therefore needed for the coherence of the manuscript.

We agree with this fair comment, and have now better integrated our in vivo electrophysiological findings in the broader context of BCSFB/BBB dysfunction (we kindly refer the Reviewer to the response to Rev3_Comment #1c.) and below in (Rev5_Comment #4b.).

Rev5_Comment #4a: Also, how supplementary video 4 related to this set of data is also not clear (…).

We appreciate the Reviewer's insightful comment on the video showing temporary, spontaneous constrictions that occur in arterioles. Interestingly, these spontaneous arteriolar tone changes could reflect altered neurovascular control secondary to cortical network hyperexcitability. Cortical hyperexcitability is well documented in Huntington's disease models and is closely linked to disrupted excitation inhibition balance and impaired GABAergic signaling (Cepeda et al., 2019; Hsu, Chang, & Chern, 2018). In parallel, specific cortical GABA interneuron subtypes can directly modulate arteriolar diameter via nitric oxide and other vasoactive mediators, providing a mechanistic route by which inhibitory circuit dysregulation could plausibly manifest as abnormal constrictions (Cauli et al., 2004; Lecrux & Hamel, 2016).

We emphasize, however, that this remains a hypothesis in our context and would necessitate a dedicated, separate study, to dissect neuronal, astrocytic, and mural cell contributions within the neurovascular unit.

In addition, we kindly refer the Reviewer to Rev5_Comment #9 where we revised the Supplementary Video 4, where we now clearly show the differences between WT and R6/2 mice.

Rev5_Comment #4b: (…) To strengthen the relevance of the results and link them to neuronal excitability, data showing that SEW2871 treatment reverse hyperexcitability in R6/2 mice would be necessary.

We thank the Reviewer for this insightful comment. Cortical hyperexcitability is a well-established HD phenotype. For example, Cepeda et al. *report that cortical pyramidal neurons in symptomatic R6/2 mice are hyperexcitable and display dysmorphic features (Cepeda et al., 2019). Importantly, chronic BBB/BCSFB dysfunction can drive such hyperexcitability via astrocytic mechanisms. In particular, sustained albumin extravasation activates astrocytic TGF-β signaling and excitatory synaptogenesis (Weissberg et al., 2015)]. Ivens* et al. *(2007) showed that astrocytic albumin uptake downregulates Kir4.1 channels and impairs K^+ buffering, producing NMDA-mediated neuronal hyperexcitability (Ivens et al., 2007). Likewise, Luo (2022) highlights that albumin/TGF-β exposure disrupts astrocytic K^+ and glutamate homeostasis, promoting network hyperexcitability (Luo, 2022). However, once albumin/TGF-β–mediated changes (e.g. excitatory synaptogenesis and K^+ channel deficits) are established, excitability should not be expected to be readily reversed (Luo, 2022; Weissberg et al., 2015).*

Accordingly, we have performed additional analysis. Expectedly, the data shows that the late-stage SEW2871 treatment did not affect the entrenched state of hyperexcitability. We kindly refer the Reviewer to the response to Rev3_Comment #1c., where we present additional analysis and interpret the data, integrating our e-phys findings into the vascular pathology narrative (response Rev3_Comment #1c.).

Rev5_Comment #5: Differential results in male or female HD patient samples are very interesting. Did authors use both female and male HD mice in the present manuscript? Do the results show sex differences as in HD patient samples? Please provide such information.

We thank the Reviewer for this important suggestion. We have now reanalyzed the data to address potential sex-specific differences in R6/2 mice, but haven't observed any overt sex-driven effects. We kindly refer the Reviewer to the response to Rev3_Comment #7, where present and discuss the results of recalculated data.

Minor Comments

Rev5_Comment #6: In human postmortem analysis, the manuscript states that cortical sections with "well-preserved vessels and meninges" were selected (PAGE 7, line 7). Clarify potential bias introduced by selecting only samples with intact meninges.

We appreciate the Reviewer's concern regarding potential sampling bias. We selected only samples with intact meninges to ensure comparable tissue preservation and to minimize sectioning artifacts at the cortical surface, which could compromise the results. This inclusion criterion was applied identically to HD and control cohorts, and we now clarify this in the revised manuscript ***[PAGE 7-8].***

Rev5_Comment #7: The observation of stronger astrocyte activation around arterioles versus venules is intriguing. Consider discussing potential mechanisms, including the possible role of astrocytic TGFβ signaling.

We agree that astrocytic TGF beta signaling is a plausible contributor to the astrocyte activation pattern. Indeed, serum albumin that extravasates into the brain parenchyma, rather than acting merely as an inert osmotic agent, may function as a signaling molecule. It binds to TGF-β receptors (TGF-βR) expressed on astrocytes. Specifically, albumin binds to the type II TGF-β receptor, which dimerizes with and activates the type I TGF-β receptor ALK5 (Verhoog et al., 2020). This leads to downregulation on homeostatic channels, i.e. Kir4.1 (impairing astrocyte capability to buffer extracellular K+, which depolarizes neuronal membranes, lowering the firing threshold, thus increasing excitability) (Verhoog et al., 2020). In addition, this signaling cascade also decreases the expression of astrocytic glutamate transporters (specifically EAAT2). This results in reduced clearance of synaptic glutamate, leading to neurotransmitter accumulation in the synaptic cleft and prolonged excitatory stimulation of neurons (Obermeier, Daneman, & Ransohoff, 2013). Crucial to the "long-lasting" nature of the hyperexcitability, albumin signaling also induces structural remodeling of the neuronal network. This pathway has been shown to induce excitatory synaptogenesis while having no effect on inhibitory synapses (Verhoog et al., 2020).

We have now infused the relevant aspects of TGF-βR -mediated signaling and albumin, as a mechanistically grounded hypothesis in the Discussion section of the revised manuscript ***[PAGE 12, 14].***

Rev5_Comment #8: Supplementary Video 3. PAGE 4, line 3: Clarify methodology for identifying perivascular macrophages.

*We thank the Reviewer for highlighting this point. In our previous work, we determined that the perivascular cells accumulating albumin during AMT disinhibition correspond to perivascular macrophages (****Response Figure 5****, (Mathiesen Janiurek et al., 2019).*

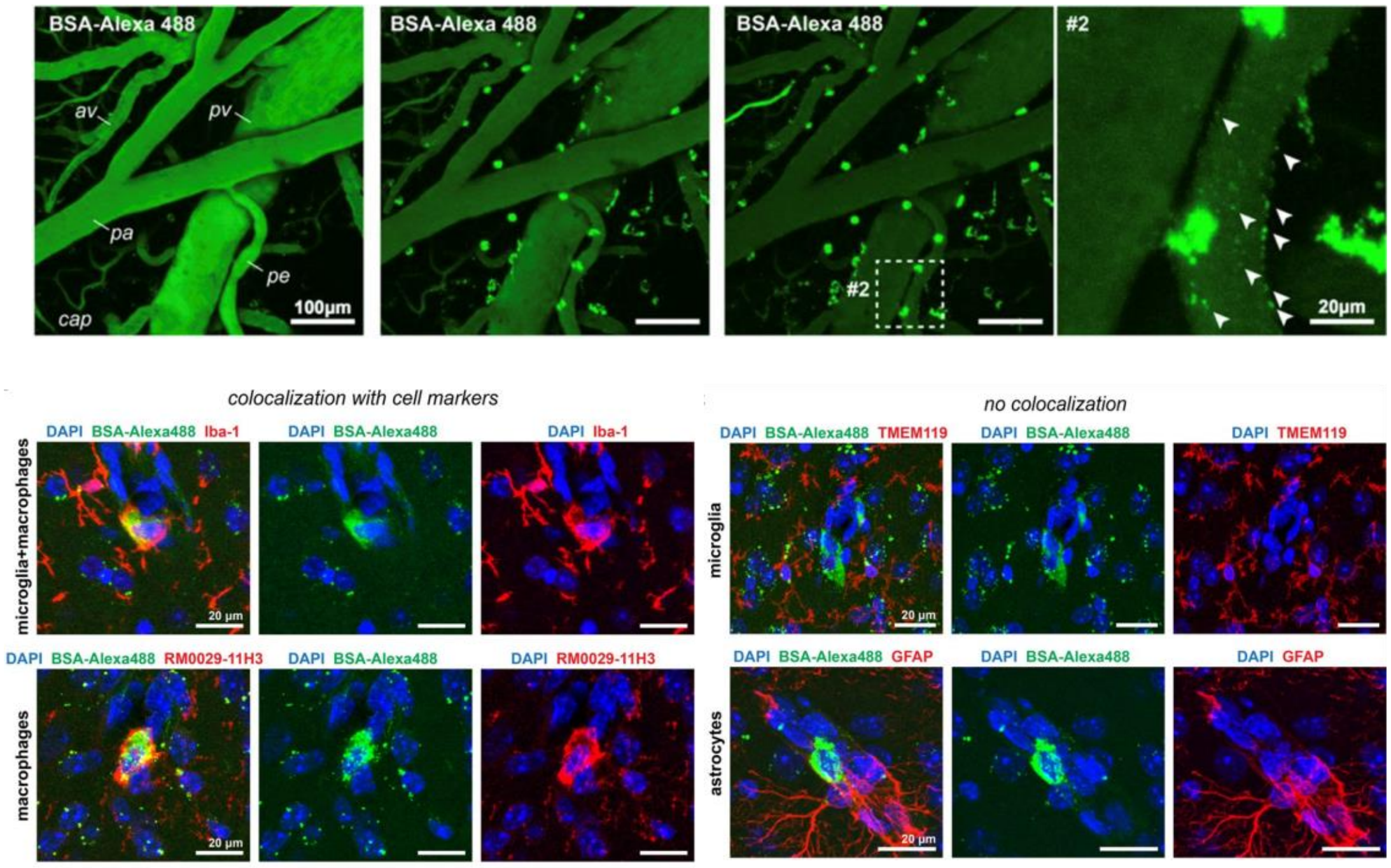

Response Figure 5. Perivascular cells that actively sequester albumin are perivascular macrophages. Upper panels are 2PM timelapse images showing albumin (BSA-A488) gradual uptake by cells occupying perivascular niche. They were identified in subsequent immunohistochemistry analyses as perivascular macrophages, and not microglia or astrocytes, by the absence of colocalization with TMEM119 and GFAP, respectively, and the positive signal from macrophage-specific RM0029-11H3 epitope. All data from (Mathiesen Janiurek et al., 2019) .

*We have now refined the text in the manuscript to explicitly state "(…) reminiscent of the perivascular macrophages that actively sequester transcytosed albumin, as identified in our earlier work (Mathiesen Janiurek et al., 2019)" **[PAGE 4]***

Of note, the observed uptake occurred under conditions replicating key features of BBB vascular organization in HD, including S1pr1-signaling–dependent AMT disinhibition and a heightened susceptibility of arterioles to AMT (Mathiesen Janiurek et al., 2019).

Rev5_Comment #9: Supplementary Video 2,3 and 4: Please provide both WT and HD videos to support data.

We have now provided corresponding WT video panels for Supplementary Videos 3, and 4 to facilitate direct comparison with the R6/2 HD phenotypes.

Supplementary Video 2.
The sole purpose of supplementary Video 2 was to illustrate the motility and dynamic behavior of AMT puncta, thereby supporting the methods used for their identification, rather than to highlight genotype-dependent differences. To clarify this, we have revised the text to state explicitly that such puncta were observed across all genotypes.
"(…) Within 120 min post-injection, and across all genotypes, we observed a gradual increase in dynamically moving AMT vesicles filled with BSA-A488 that was endocytosed from the bloodstream (…)"
We would like to note that at this magnification, which resolves individual vesicles within a small vessel segment, genotype differences cannot be reliably assessed.

Supplementary Video 3.
*In addition to the requested WT negative control, we now also provide a WT positive control, in which blood-circulating albumin (BSA-A488) was laser-extravasated into the perivascular space. The increased presence of BSA-A488 on the abluminal side, arising either from enhanced AMT in R6/2 mice or from albumin influx across a compromised barrier, is actively sequestered by cells occupying the niche of perivascular macrophage (**Response Figure 6**).*

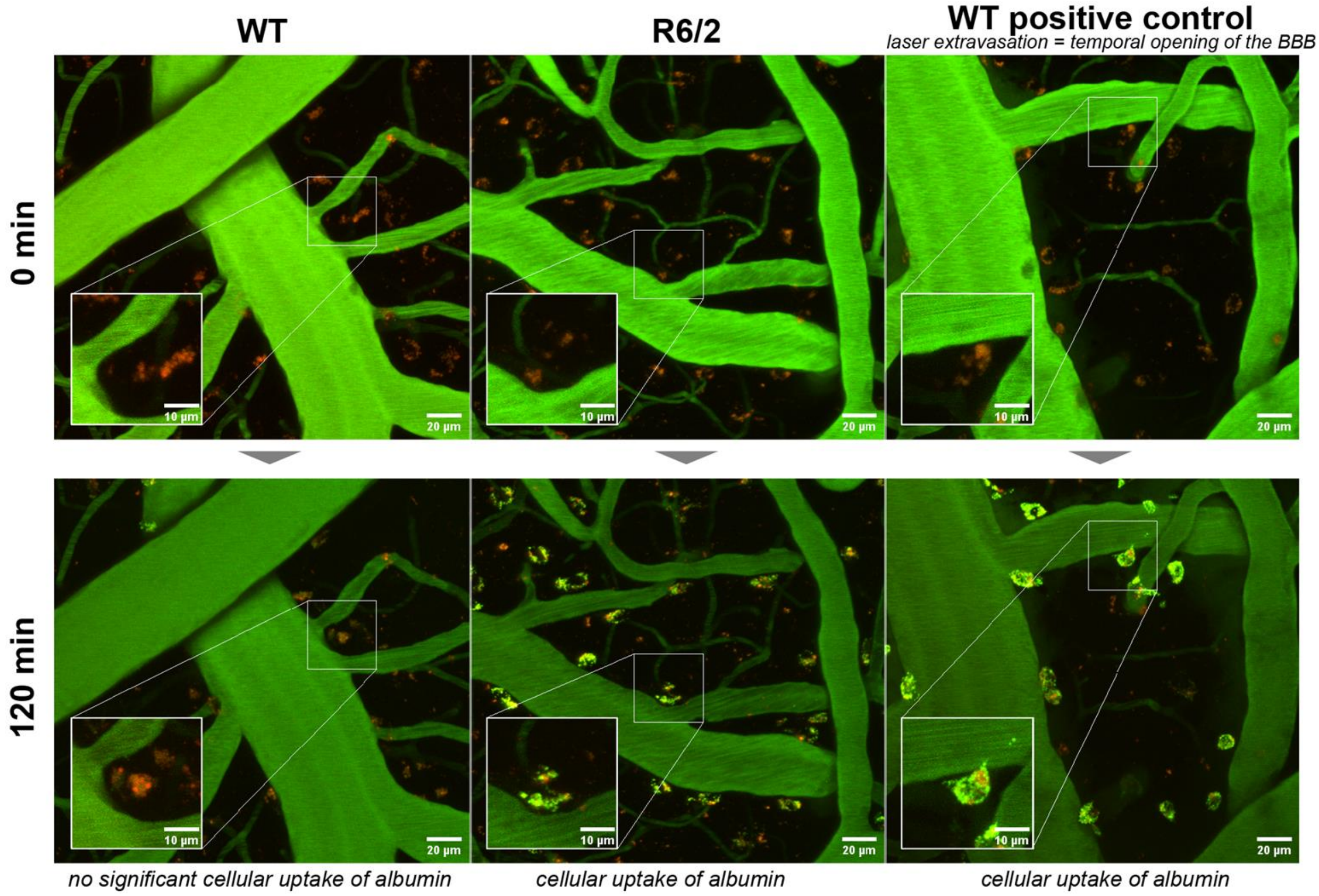

Response Figure 6. Parenchymal uptake of BSA-A488 over time. Magnified square insets highlight representative perivascular cells with evident uptake in R6/2 mice, but not in WT mice. As a positive control, blood-circulating BSA-A488 was laser-extravasated into the perivascular space in WT mice by transient laser-induced BBB opening. This demonstrates that increased uptake is driven by greater abluminal albumin exposure, rather than by an intrinsic change in the properties of the perivascular cells. Increased abluminal BSA-A488, whether arising from disinhibited AMT in R6/2 mice or from laser-induced extravasation in WT mice, is actively sequestered by cells occupying the perivascular macrophage niche. Time is relative to BSA-A488 i.v. injection.

Supplementary Video 4.

*In addition to the requested WT negative control video, and constriction markers (arrowheads), we now also provide a color-coded temporal map of vessel diameter changes, evident in R6/2, but not in WT mice (lower panels); (**Response Figure 7**).*

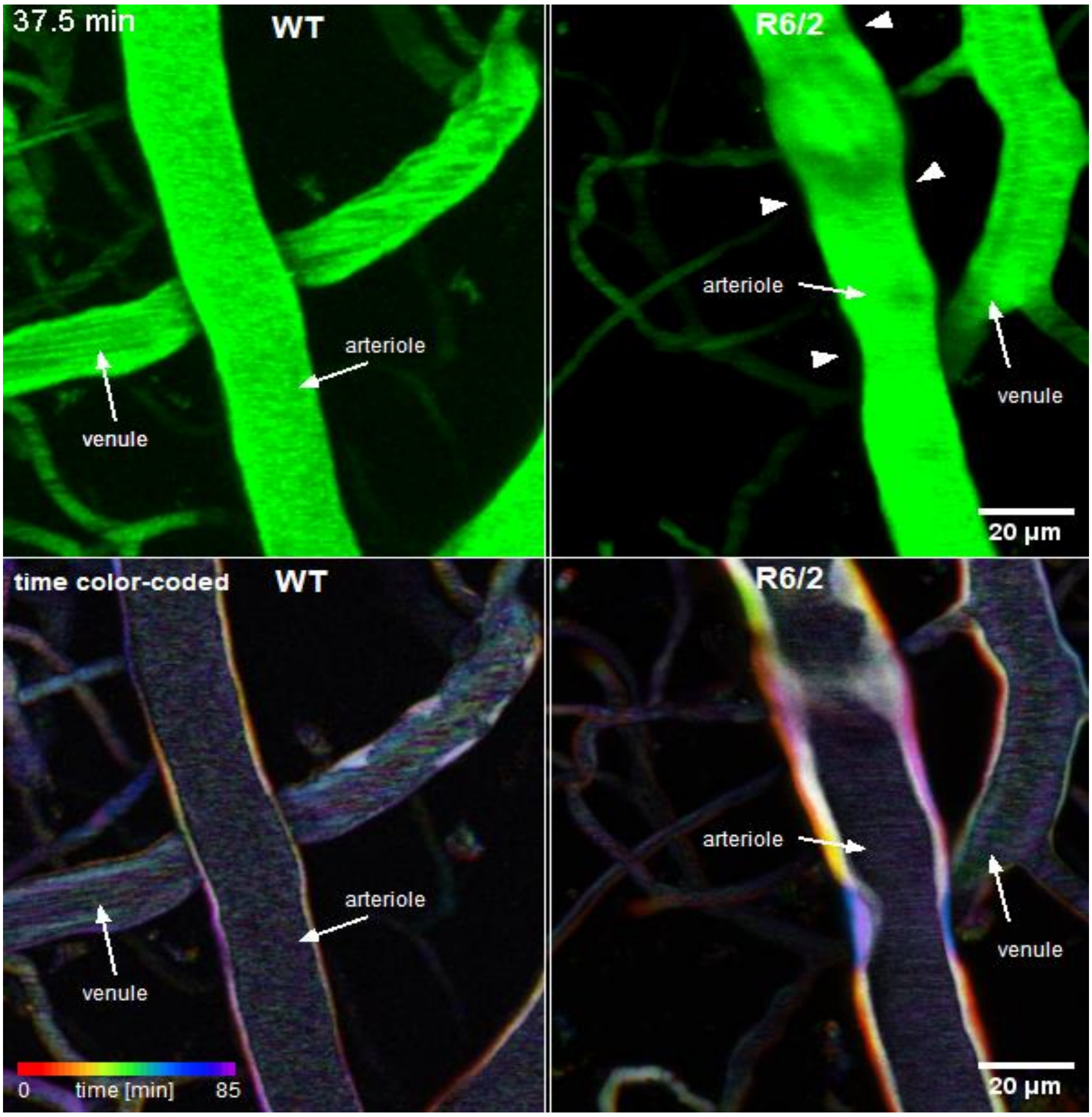

Response Figure 7*:* Snapshot of a timelapse recording of cortical vessels dynamics. Upper panels show spontaneous constrictions of cerebral arterioles present in R6/2 mice (arrowheads). Lower panels show color-coded temporal maps of vessel diameter changes, evident in R6/2, but not in WT mice. In contrast, venules exhibit no constrictions, regardless of the genotype.

Rev5_Comment #10: Paracellular permeability experiments: Clarify whether sequential measurements were performed in the same animals and, if so, specify the time intervals between assays.

> *We thank the Reviewer for highlighting this point. The scanning of anatomically distinct cortical regions in paracellular permeability experiments: the sub-pial volume (0-60 µm), primarily containing large BCSFB vessels and the capillary bed volume (120-180 µm) with vessels containing the BBB (Figure 1C) occurred simultaneously and within the same animals.*
>
> *We have now revised the Results text [**PAGE 3**]., and the **Supplementary Video 1** legend to better convey this information.*
>
> *In addition, regarding AMT and NaFluo assessments, we also clarified that also AMT and NaFluo leakage scans was performed in the same animals in a sequential matter:*
>
>> *"(…) All AMT and NaFluo leakage imaging was performed sequentially in the same animals. AMT was assessed first using BSA-Alexa488, which produces discrete, low intensity punctate signals, followed by evaluation of paracellular permeability with NaFluo, which generates a diffuse parenchymal signal several orders of magnitude brighter. Accordingly, laser power and detector sensitivity were reduced for NaFluo acquisition, effectively suppressing any residual BSA signal to background levels. NaFluo imaging was initiated 30 minutes after completion of BSA imaging. This approach enabled direct comparison of AMT and paracellular permeability within the same animals. As an internal control, TRITC-dextran was intravenously injected to confirm preserved barrier integrity and exclude any potential imaging-induced damage that could confound NaFluo measurements. (…)"*
>
> *This information can be now found in the revised Methods section on two-photon in vivo imaging setup and scanning timeline **[PAGE 17].***

Rev5_Comment #11: Abstract. Line 27, should say "reversed" instead of "rescued"

> *We agree with the Reviewer's point and recognize the fine nuance between the terms. We have now corrected the verbiage from 'rescued" to "reversed" throughout the revised manuscript, including the title.*

RESPONSES REFERENCES

Andreone, B. J., Chow, B. W., Tata, A., Lacoste, B., Ben-Zvi, A., Bullock, K., Deik, A. A., Ginty, D. D., Clish, C. B., & Gu, C. (2017). Blood-Brain Barrier Permeability Is Regulated by Lipid Transport-Dependent Suppression of Caveolae-Mediated Transcytosis. *Neuron*, *94*(3), 581-594 e585. https://doi.org/10.1016/j.neuron.2017.03.043

Armulik, A., Genove, G., Mae, M., Nisancioglu, M. H., Wallgard, E., Niaudet, C., He, L., Norlin, J., Lindblom, P., Strittmatter, K., Johansson, B. R., & Betsholtz, C. (2010). Pericytes regulate the blood-brain barrier. *Nature*, *468*(7323), 557-561. https://doi.org/10.1038/nature09522

Bar-Klein, G., Cacheaux, L. P., Kamintsky, L., Prager, O., Weissberg, I., Schoknecht, K., Cheng, P., Kim, S. Y., Wood, L., Heinemann, U., Kaufer, D., & Friedman, A. (2014). Losartan prevents acquired epilepsy via TGF-beta signaling suppression. *Annals of neurology*, *75*(6), 864-875. https://doi.org/10.1002/ana.24147

Barretto, R. P., Ko, T. H., Jung, J. C., Wang, T. J., Capps, G., Waters, A. C., Ziv, Y., Attardo, A., Recht, L., & Schnitzer, M. J. (2011). Time-lapse imaging of disease progression in deep brain areas using fluorescence microendoscopy. *Nat Med*, *17*(2), 223-228. https://doi.org/10.1038/nm.2292

Barry, J., Bui, M. T. N., Levine, M. S., & Cepeda, C. (2022). Synaptic pathology in Huntington's disease: Beyond the corticostriatal pathway. *Neurobiol Dis*, *162*, 105574. https://doi.org/10.1016/j.nbd.2021.105574

Bayram-Weston, Z., Torres, E. M., Jones, L., Dunnett, S. B., & Brooks, S. P. (2012). Light and electron microscopic characterization of the evolution of cellular pathology in the Hdh(CAG)150 Huntington's disease knock-in mouse. *Brain Res Bull*, *88*(2-3), 189-198. https://doi.org/10.1016/j.brainresbull.2011.03.014

Ben-Zvi, A., Lacoste, B., Kur, E., Andreone, B. J., Mayshar, Y., Yan, H., & Gu, C. (2014). Mfsd2a is critical for the formation and function of the blood-brain barrier. *Nature*, *509*(7501), 507-511. https://doi.org/10.1038/nature13324

Blaho, V. A., Galvani, S., Engelbrecht, E., Liu, C., Swendeman, S. L., Kono, M., Proia, R. L., Steinman, L., Han, M. H., & Hla, T. (2015). HDL-bound sphingosine-1-phosphate restrains lymphopoiesis and neuroinflammation. *Nature*, *523*(7560), 342-346. https://doi.org/10.1038/nature14462

Bolden, C. T., Olson, S. D., & Cox, C. S., Jr. (2023). A decade of blood-brain barrier permeability assays: Revisiting old traumatic brain injury rat data for new insights and experimental design. *Microvasc Res*, *145*, 104453. https://doi.org/10.1016/j.mvr.2022.104453

Cacheaux, L. P., Ivens, S., David, Y., Lakhter, A. J., Bar-Klein, G., Shapira, M., Heinemann, U., Friedman, A., & Kaufer, D. (2009). Transcriptome profiling reveals TGF-beta signaling involvement in epileptogenesis. *J Neurosci*, *29*(28), 8927-8935. https://doi.org/10.1523/JNEUROSCI.0430-09.2009

Cai, C., Fordsmann, J. C., Jensen, S. H., Gesslein, B., Lonstrup, M., Hald, B. O., Zambach, S. A., Brodin, B., & Lauritzen, M. J. (2018). Stimulation-induced increases in cerebral blood flow and local capillary vasoconstriction depend on conducted vascular responses. *Proc Natl Acad Sci U S A*, *115*(25), E5796-E5804. https://doi.org/10.1073/pnas.1707702115

Cankar, N., Beschorner, N., Tsopanidou, A., Qvist, F. L., Colaco, A. R., Andersen, M., Kjaerby, C., Delle, C., Lambert, M., Mundt, F., Weikop, P., Jucker, M., Mann, M., Skotte, N. H., & Nedergaard, M. (2024). Sleep deprivation leads to non-adaptive alterations in sleep microarchitecture and amyloid-beta accumulation in a murine Alzheimer model. *Cell Rep*, *43*(11), 114977. https://doi.org/10.1016/j.celrep.2024.114977

Cauli, B., Tong, X. K., Rancillac, A., Serluca, N., Lambolez, B., Rossier, J., & Hamel, E. (2004). Cortical GABA interneurons in neurovascular coupling: relays for subcortical vasoactive pathways. *J Neurosci*, *24*(41), 8940-8949. https://doi.org/10.1523/JNEUROSCI.3065-04.2004

Caviston, J. P., & Holzbaur, E. L. (2009). Huntingtin as an essential integrator of intracellular vesicular trafficking. *Trends Cell Biol*, *19*(4), 147-155. https://doi.org/10.1016/j.tcb.2009.01.005

Cepeda-Prado, E., Popp, S., Khan, U., Stefanov, D., Rodriguez, J., Menalled, L. B., Dow-Edwards, D., Small, S. A., & Moreno, H. (2012). R6/2 Huntington's disease mice develop early and progressive abnormal brain metabolism and seizures. *J Neurosci*, *32*(19), 6456-6467. https://doi.org/10.1523/JNEUROSCI.0388-12.2012

Cepeda, C., Cummings, D. M., Andre, V. M., Holley, S. M., & Levine, M. S. (2010). Genetic mouse models of Huntington's disease: focus on electrophysiological mechanisms. *ASN Neuro*, *2*(2), e00033. https://doi.org/10.1042/AN20090058

Cepeda, C., Oikonomou, K. D., Cummings, D., Barry, J., Yazon, V. W., Chen, D. T., Asai, J., Williams, C. K., & Vinters, H. V. (2019). Developmental origins of cortical hyperexcitability in Huntington's disease: Review and new observations. *Journal of neuroscience research*, *97*(12), 1624-1635. https://doi.org/10.1002/jnr.24503

Chan, S. T., Mercaldo, N. D., Kwong, K. K., Hersch, S. M., & Rosas, H. D. (2021). Impaired Cerebrovascular Reactivity in Huntington's Disease. *Front Physiol*, *12*, 663898. https://doi.org/10.3389/fphys.2021.663898

Cheng, J., Korte, N., Nortley, R., Sethi, H., Tang, Y., & Attwell, D. (2018). Targeting pericytes for therapeutic approaches to neurological disorders. *Acta Neuropathol*, *136*(4), 507-523. https://doi.org/10.1007/s00401-018-1893-0

Chow, B. W., Nunez, V., Kaplan, L., Granger, A. J., Bistrong, K., Zucker, H. L., Kumar, P., Sabatini, B. L., & Gu, C. (2020). Caveolae in CNS arterioles mediate neurovascular coupling. *Nature*, *579*(7797), 106-110. https://doi.org/10.1038/s41586-020-2026-1

Coleman, A., Langan, M. T., Verma, G., Knights, H., Sturrock, A., Leavitt, B. R., Tabrizi, S. J., Scahill, R. I., & Hobbs, N. Z. (2024). Assessment of Perivascular Space Morphometry Across the White Matter in Huntington's Disease Using MRI. *J Huntingtons Dis*, *13*(1), 91-101. https://doi.org/10.3233/JHD-231508

Cramer, S. W., Carter, R. E., Aronson, J. D., Kodandaramaiah, S. B., Ebner, T. J., & Chen, C. C. (2021). Through the looking glass: A review of cranial window technology for optical access to the brain. *J Neurosci Methods*, *354*, 109100. https://doi.org/10.1016/j.jneumeth.2021.109100

Cruz-Orengo, L., Holman, D. W., Dorsey, D., Zhou, L., Zhang, P., Wright, M., McCandless, E. E., Patel, J. R., Luker, G. D., Littman, D. R., Russell, J. H., & Klein, R. S. (2011). CXCR7 influences leukocyte entry into the CNS parenchyma by controlling abluminal CXCL12 abundance during autoimmunity. *J Exp Med*, *208*(2), 327-339. https://doi.org/10.1084/jem.20102010

Cummings, D. M., Alaghband, Y., Hickey, M. A., Joshi, P. R., Hong, S. C., Zhu, C., Ando, T. K., Andre, V. M., Cepeda, C., Watson, J. B., & Levine, M. S. (2012). A critical window of CAG repeat-length correlates with phenotype severity in the R6/2 mouse model of Huntington's disease. *J Neurophysiol*, *107*(2), 677-691. https://doi.org/10.1152/jn.00762.2011

Daneman, R., Zhou, L., Kebede, A. A., & Barres, B. A. (2010). Pericytes are required for blood-brain barrier integrity during embryogenesis. *Nature*, *468*(7323), 562-566. https://doi.org/10.1038/nature09513

del Toro, D., Alberch, J., Lazaro-Dieguez, F., Martin-Ibanez, R., Xifro, X., Egea, G., & Canals, J. M. (2009). Mutant huntingtin impairs post-Golgi trafficking to lysosomes by delocalizing optineurin/Rab8 complex from the Golgi apparatus. *Mol Biol Cell*, *20*(5), 1478-1492. https://doi.org/10.1091/mbc.e08-07-0726

Delgardo, M., Tang, A. J., Tudor, T., Pascual-Leone, A., & Connolly, E. S., Jr. (2023). Role of gC1qR as a modulator of endothelial cell permeability and contributor to post-stroke inflammation and edema formation. *Front Cell Neurosci*, *17*, 1123365. https://doi.org/10.3389/fncel.2023.1123365

Di Pardo, A., Amico, E., Basit, A., Armirotti, A., Joshi, P., Neely, M. D., Vuono, R., Castaldo, S., Digilio, A. F., Scalabri, F., Pepe, G., Elifani, F., Madonna, M., Jeong, S. K., Park, B. M., D'Esposito, M., Bowman, A. B., Barker, R. A., & Maglione, V. (2017). Defective Sphingosine-1-phosphate metabolism is a druggable target in Huntington's disease. *Sci Rep*, *7*(1), 5280. https://doi.org/10.1038/s41598-017-05709-y

Di Pardo, A., Amico, E., Favellato, M., Castrataro, R., Fucile, S., Squitieri, F., & Maglione, V. (2014). FTY720 (fingolimod) is a neuroprotective and disease-modifying agent in cellular and mouse models of Huntington disease. *Hum Mol Genet*, *23*(9), 2251-2265. https://doi.org/10.1093/hmg/ddt615

Di Pardo, A., Amico, E., Scalabri, F., Pepe, G., Castaldo, S., Elifani, F., Capocci, L., De Sanctis, C., Comerci, L., Pompeo, F., D'Esposito, M., Filosa, S., Crispi, S., & Maglione, V. (2017). Impairment of blood-brain barrier is an early event in R6/2 mouse model of Huntington Disease. *Sci Rep*, *7*, 41316. https://doi.org/10.1038/srep41316

Dong, J., Wang, H., Wu, G., Zhao, J., Zhang, L., Zuo, L., Zhu, W., Gong, J., Li, Y., Gu, L., & Li, J. (2014). Oral treatment with SEW2871, a sphingosine-1-phosphate type 1 receptor agonist, ameliorates experimental colitis in interleukin-10 gene deficient mice. *Clin Exp Immunol*, *177*(1), 94-101. https://doi.org/10.1111/cei.12304

Drouin-Ouellet, J., Sawiak, S. J., Cisbani, G., Lagace, M., Kuan, W. L., Saint-Pierre, M., Dury, R. J., Alata, W., St-Amour, I., Mason, S. L., Calon, F., Lacroix, S., Gowland, P. A., Francis, S. T., Barker, R. A., & Cicchetti, F. (2015). Cerebrovascular and blood-brain barrier impairments in Huntington's disease: Potential implications for its pathophysiology. *Annals of neurology*, *78*(2), 160-177. https://doi.org/10.1002/ana.24406

Etxeberria-Rekalde, E., Alzola-Aldamizetxebarria, S., Flunkert, S., Hable, I., Daurer, M., Neddens, J., & Hutter-Paier, B. (2020). Quantification of Huntington's Disease Related Markers in the R6/2 Mouse Model. *Front Mol Neurosci*, *13*, 617229. https://doi.org/10.3389/fnmol.2020.617229

Flemming, S., Burkard, N., Meir, M., Schick, M. A., Germer, C. T., & Schlegel, N. (2018). Sphingosine-1-Phosphate Receptor-1 Agonist Sew2871 Causes Severe Cardiac Side Effects and Does Not Improve Microvascular Barrier Breakdown in Sepsis. *Shock*, *49*(1), 71-81. https://doi.org/10.1097/SHK.0000000000000908

Fordsmann, J. C., Murmu, R. P., Cai, C., Brazhe, A., Thomsen, K. J., Zambach, S. A., Lonstrup, M., Lind, B. L., & Lauritzen, M. (2019). Spontaneous astrocytic Ca(2+) activity abounds in electrically suppressed ischemic penumbra of aged mice. *Glia*, *67*(1), 37-52. https://doi.org/10.1002/glia.23506

Franciosi, S., Ryu, J. K., Shim, Y., Hill, A., Connolly, C., Hayden, M. R., McLarnon, J. G., & Leavitt, B. R. (2012). Age-dependent neurovascular abnormalities and altered microglial morphology in the YAC128 mouse model of Huntington disease. *Neurobiol Dis*, *45*(1), 438-449. https://doi.org/10.1016/j.nbd.2011.09.003

Garcia-Castillo, M. D., Chinnapen, D. J., & Lencer, W. I. (2017). Membrane Transport across Polarized Epithelia. *Cold Spring Harb Perspect Biol*, *9*(9). https://doi.org/10.1101/cshperspect.a027912

Garcia, F. J., Sun, N., Lee, H., Godlewski, B., Mathys, H., Galani, K., Zhou, B., Jiang, X., Ng, A. P., Mantero, J., Tsai, L. H., Bennett, D. A., Sahin, M., Kellis, M., & Heiman, M. (2022). Single-cell dissection of the human brain vasculature. *Nature*, *603*(7903), 893-899. https://doi.org/10.1038/s41586-022-04521-7

Geyer, P. E., Holdt, L. M., Teupser, D., & Mann, M. (2017). Revisiting biomarker discovery by plasma proteomics. *Mol Syst Biol*, *13*(9), 942. https://doi.org/10.15252/msb.20156297

Grubb, S., Cai, C., Hald, B. O., Khennouf, L., Murmu, R. P., Jensen, A. G. K., Fordsmann, J., Zambach, S., & Lauritzen, M. (2020). Precapillary sphincters maintain perfusion in the cerebral cortex. *Nat Commun*, *11*(1), 395. https://doi.org/10.1038/s41467-020-14330-z

Guldbrandsen, A., Vethe, H., Farag, Y., Oveland, E., Garberg, H., Berle, M., Myhr, K. M., Opsahl, J. A., Barsnes, H., & Berven, F. S. (2014). In-depth characterization of the cerebrospinal fluid (CSF) proteome displayed through the CSF proteome resource (CSF-PR). *Mol Cell Proteomics*, *13*(11), 3152-3163. https://doi.org/10.1074/mcp.M114.038554

Gunther, R., Krause, E., Schumann, M., Blasig, I. E., & Haseloff, R. F. (2015). Depletion of highly abundant proteins from human cerebrospinal fluid: a cautionary note. *Mol Neurodegener*, *10*, 53. https://doi.org/10.1186/s13024-015-0050-7

Hall, C. N., Reynell, C., Gesslein, B., Hamilton, N. B., Mishra, A., Sutherland, B. A., O'Farrell, F. M., Buchan, A. M., Lauritzen, M., & Attwell, D. (2014). Capillary pericytes regulate cerebral blood flow in health and disease. *Nature*, *508*(7494), 55-60. https://doi.org/10.1038/nature13165

Hannun, Y. A., & Obeid, L. M. (2008). Principles of bioactive lipid signalling: lessons from sphingolipids. *Nat Rev Mol Cell Biol*, *9*(2), 139-150. https://doi.org/10.1038/nrm2329

Haqqani, A. S., Belanger, K., & Stanimirovic, D. B. (2024). Receptor-mediated transcytosis for brain delivery of therapeutics: receptor classes and criteria. *Front Drug Deliv*, *4*, 1360302. https://doi.org/10.3389/fddev.2024.1360302

Harding, I. C., Mitra, R., Mensah, S. A., Herman, I. M., & Ebong, E. E. (2018). Pro-atherosclerotic disturbed flow disrupts caveolin-1 expression, localization, and function via glycocalyx degradation. *J Transl Med*, *16*(1), 364. https://doi.org/10.1186/s12967-018-1721-2

Hattula, K., & Peranen, J. (2000). FIP-2, a coiled-coil protein, links Huntingtin to Rab8 and modulates cellular morphogenesis. *Current Biology*, *10*(24), 1603-1606. https://doi.org/10.1016/s0960-9822(00)00864-2

Heinemann, U., Kaufer, D., & Friedman, A. (2012). Blood-brain barrier dysfunction, TGFbeta signaling, and astrocyte dysfunction in epilepsy. *Glia*, *60*(8), 1251-1257. https://doi.org/10.1002/glia.22311

Hill, R. A., Tong, L., Yuan, P., Murikinati, S., Gupta, S., & Grutzendler, J. (2015). Regional Blood Flow in the Normal and Ischemic Brain Is Controlled by Arteriolar Smooth Muscle Cell Contractility and Not by Capillary Pericytes. *Neuron*, *87*(1), 95-110. https://doi.org/10.1016/j.neuron.2015.06.001

Hsiao, H. Y., Chen, Y. C., Huang, C. H., Chen, C. C., Hsu, Y. H., Chen, H. M., Chiu, F. L., Kuo, H. C., Chang, C., & Chern, Y. (2015). Aberrant astrocytes impair vascular reactivity in Huntington disease. *Annals of neurology*, *78*(2), 178-192. https://doi.org/10.1002/ana.24428

Hsu, Y. T., Chang, Y. G., & Chern, Y. (2018). Insights into GABA(A)ergic system alteration in Huntington's disease. *Open Biol*, *8*(12). https://doi.org/10.1098/rsob.180165

Hu, N., Chen, Z., Zhao, X., Peng, X., Wu, Y., Yang, K., & Sun, T. (2025). Endothelial Dysfunction in Huntington's Disease: Pathophysiology and Therapeutic Implications. *Int J Mol Sci*, *26*(4). https://doi.org/10.3390/ijms26041432

Ichijo, M., Ishibashi, S., Li, F., Yui, D., Miki, K., Mizusawa, H., & Yokota, T. (2015). Sphingosine-1-Phosphate Receptor-1 Selective Agonist Enhances Collateral Growth and Protects against Subsequent Stroke. *PLoS One*, *10*(9), e0138029. https://doi.org/10.1371/journal.pone.0138029

Ignjatovic, V., Geyer, P. E., Palaniappan, K. K., Chaaban, J. E., Omenn, G. S., Baker, M. S., Deutsch, E. W., & Schwenk, J. M. (2019). Mass Spectrometry-Based Plasma Proteomics: Considerations from Sample Collection to Achieving Translational Data. *J Proteome Res*, *18*(12), 4085-4097. https://doi.org/10.1021/acs.jproteome.9b00503

Isshiki, M., & Okabe, S. (2014). Evaluation of cranial window types for in vivo two-photon imaging of brain microstructures. *Microscopy (Oxf)*, *63*(1), 53-63. https://doi.org/10.1093/jmicro/dft043

Ivens, S., Kaufer, D., Flores, L. P., Bechmann, I., Zumsteg, D., Tomkins, O., Seiffert, E., Heinemann, U., & Friedman, A. (2007). TGF-beta receptor-mediated albumin uptake into astrocytes is involved in neocortical epileptogenesis. *Brain : a journal of neurology*, *130*(Pt 2), 535-547. https://doi.org/10.1093/brain/awl317

Jiang, R., Diaz-Castro, B., Looger, L. L., & Khakh, B. S. (2016). Dysfunctional Calcium and Glutamate Signaling in Striatal Astrocytes from Huntington's Disease Model Mice. *J Neurosci*, *36*(12), 3453-3470. https://doi.org/10.1523/JNEUROSCI.3693-15.2016

Jo, E., Sanna, M. G., Gonzalez-Cabrera, P. J., Thangada, S., Tigyi, G., Osborne, D. A., Hla, T., Parrill, A. L., & Rosen, H. (2005). S1P1-selective in vivo-active agonists from high-throughput screening: off-the-shelf chemical probes of receptor interactions, signaling, and fate. *Chem Biol*, *12*(6), 703-715. https://doi.org/10.1016/j.chembiol.2005.04.019

Jung, J. C., Mehta, A. D., Aksay, E., Stepnoski, R., & Schnitzer, M. J. (2004). In vivo mammalian brain imaging using one- and two-photon fluorescence microendoscopy. *J Neurophysiol*, *92*(5), 3121-3133. https://doi.org/10.1152/jn.00234.2004

Khennouf, L., Gesslein, B., Brazhe, A., Octeau, J. C., Kutuzov, N., Khakh, B. S., & Lauritzen, M. (2018). Active role of capillary pericytes during stimulation-induced activity and spreading depolarization. *Brain : a journal of neurology*, *141*(7), 2032-2046. https://doi.org/10.1093/brain/awy143

Khoury, N., Pizzo, M. E., Discenza, C. B., Joy, D., Tatarakis, D., Todorov, M. I., Negwer, M., Ha, C., De Melo, G. L., Sarrafha, L., Simon, M. J., Chan, D., Chau, R., Chew, K. S., Chow, J., Clemens, A., Robles-Colmenares, Y., Dugas, J. C., Duque, J., . . . Yu Zuchero, Y. J. (2025). Fc-engineered large molecules targeting blood-brain barrier transferrin receptor and CD98hc have distinct central nervous system and peripheral biodistribution. *Nat Commun*, *16*(1), 1822. https://doi.org/10.1038/s41467-025-57108-x

Knowland, D., Arac, A., Sekiguchi, K. J., Hsu, M., Lutz, S. E., Perrino, J., Steinberg, G. K., Barres, B. A., Nimmerjahn, A., & Agalliu, D. (2014). Stepwise recruitment of transcellular and paracellular pathways underlies blood-brain barrier breakdown in stroke. *Neuron*, *82*(3), 603-617. https://doi.org/10.1016/j.neuron.2014.03.003

Kondo, N., Ogawa, M., Wada, H., & Nishikawa, S. (2009). Thrombin induces rapid disassembly of claudin-5 from the tight junction of endothelial cells. *Exp Cell Res*, *315*(17), 2879-2887. https://doi.org/10.1016/j.yexcr.2009.07.031

Kouser, L., Madhukaran, S. P., Shastri, A., Saraon, A., Ferluga, J., Al-Mozaini, M., & Kishore, U. (2015). Emerging and Novel Functions of Complement Protein C1q. *Front Immunol*, *6*, 317. https://doi.org/10.3389/fimmu.2015.00317

Kucharz, K., Kristensen, K., Johnsen, K. B., Lund, M. A., Lonstrup, M., Moos, T., Andresen, T. L., & Lauritzen, M. J. (2021). Post-capillary venules are the key locus for transcytosis-mediated brain delivery of therapeutic nanoparticles. *Nat Commun*, *12*(1), 4121. https://doi.org/10.1038/s41467-021-24323-1

Kucharz, K., Kutuzov, N., Zhukov, O., Mathiesen Janiurek, M., & Lauritzen, M. (2022). Shedding Light on the Blood–Brain Barrier Transport with Two-Photon Microscopy In Vivo. *Pharmaceutical Research*, *39*(7), 1457-1468. https://doi.org/10.1007/s11095-022-03266-2

Kucharz, K., & Lauritzen, M. (2018). CaMKII-dependent endoplasmic reticulum fission by whisker stimulation and during cortical spreading depolarization. *Brain : a journal of neurology*, *141*(4), 1049-1062. https://doi.org/10.1093/brain/awy036

Kucharz, K., Mathiesen Janiurek, M., Christoffersen, C., & Lauritzen, M. (2022). Two-photon microscopy in vivo reveals brain vessel type-specific loss of glycocalyx caused by apoM/S1P signaling impairment. *bioRxiv*, 2022.2004.2011.487803. https://doi.org/10.1101/2022.04.11.487803

Kucharz, K., Sondergaard Rasmussen, I., Bach, A., Stromgaard, K., & Lauritzen, M. (2017). PSD-95 uncoupling from NMDA receptors by Tat-N-dimer ameliorates neuronal depolarization in cortical spreading depression. *J Cereb Blood Flow Metab*, *37*(5), 1820-1828. https://doi.org/10.1177/0271678X16645595

Kutuzov, N., Flyvbjerg, H., & Lauritzen, M. (2018). Contributions of the glycocalyx, endothelium, and extravascular compartment to the blood-brain barrier. *Proc Natl Acad Sci U S A*, *115*(40), E9429-E9438. https://doi.org/10.1073/pnas.1802155115

Lecrux, C., & Hamel, E. (2016). Neuronal networks and mediators of cortical neurovascular coupling responses in normal and altered brain states. *Philos Trans R Soc Lond B Biol Sci*, *371*(1705). https://doi.org/10.1098/rstb.2015.0350

Li, G., Zhang, H., Liu, M., Liu, Y., Shen, S., Li, S., & Zhu, W. (2025). The S1PR1-CCN1 axis drives endothelial-to-mesenchymal transition and vascular instability in brain arteriovenous malformations. *Eur J Med Res*, *30*(1), 1175. https://doi.org/10.1186/s40001-025-03484-5

Liddelow, S. A., Guttenplan, K. A., Clarke, L. E., Bennett, F. C., Bohlen, C. J., Schirmer, L., Bennett, M. L., Munch, A. E., Chung, W. S., Peterson, T. C., Wilton, D. K., Frouin, A., Napier, B. A., Panicker, N., Kumar, M., Buckwalter, M. S., Rowitch, D. H., Dawson, V. L., Dawson, T. M., . . . Barres, B. A. (2017). Neurotoxic reactive astrocytes are induced by activated microglia. *Nature*, *541*(7638), 481-487. https://doi.org/10.1038/nature21029

Lin, C. Y., Hsu, Y. H., Lin, M. H., Yang, T. H., Chen, H. M., Chen, Y. C., Hsiao, H. Y., Chen, C. C., Chern, Y., & Chang, C. (2013). Neurovascular abnormalities in humans and mice with Huntington's disease. *Experimental Neurology*, *250*, 20-30. https://doi.org/10.1016/j.expneurol.2013.08.019

Lind, B. L., Brazhe, A. R., Jessen, S. B., Tan, F. C., & Lauritzen, M. J. (2013). Rapid stimulus-evoked astrocyte Ca2+ elevations and hemodynamic responses in mouse somatosensory cortex in vivo. *Proc Natl Acad Sci U S A*, *110*(48), E4678-4687. https://doi.org/10.1073/pnas.1310065110

Liu, T., Qian, W. J., Mottaz, H. M., Gritsenko, M. A., Norbeck, A. D., Moore, R. J., Purvine, S. O., Camp, D. G., 2nd, & Smith, R. D. (2006). Evaluation of multiprotein immunoaffinity subtraction for plasma proteomics and candidate biomarker discovery using mass spectrometry. *Mol Cell Proteomics*, *5*(11), 2167-2174. https://doi.org/10.1074/mcp.T600039-MCP200

Luo, J. (2022). TGF-beta as a Key Modulator of Astrocyte Reactivity: Disease Relevance and Therapeutic Implications. *Biomedicines*, *10*(5). https://doi.org/10.3390/biomedicines10051206

Man, S., Tucky, B., Cotleur, A., Drazba, J., Takeshita, Y., & Ransohoff, R. M. (2012). CXCL12-induced monocyte-endothelial interactions promote lymphocyte transmigration across an in vitro blood-brain barrier. *Sci Transl Med*, *4*(119), 119ra114. https://doi.org/10.1126/scitranslmed.3003197

Mantovani, S., Gordon, R., Li, R., Christie, D. C., Kumar, V., & Woodruff, T. M. (2016). Motor deficits associated with Huntington's disease occur in the absence of striatal degeneration in BACHD transgenic mice. *Hum Mol Genet*, *25*(9), 1780-1791. https://doi.org/10.1093/hmg/ddw050

Mathiesen Janiurek, M., Soylu-Kucharz, R., Christoffersen, C., Kucharz, K., & Lauritzen, M. (2019). Apolipoprotein M-bound sphingosine-1-phosphate regulates blood-brain barrier paracellular permeability and transcytosis. *Elife*, *8*. https://doi.org/10.7554/eLife.49405

McCandless, E. E., Wang, Q., Woerner, B. M., Harper, J. M., & Klein, R. S. (2006). CXCL12 limits inflammation by localizing mononuclear infiltrates to the perivascular space during experimental autoimmune encephalomyelitis. *J Immunol*, *177*(11), 8053-8064. https://doi.org/10.4049/jimmunol.177.11.8053

Menalled, L. B. (2005). Knock-in mouse models of Huntington's disease. *NeuroRx*, *2*(3), 465-470. https://doi.org/10.1602/neurorx.2.3.465

Miguez, A., Garcia-Diaz Barriga, G., Brito, V., Straccia, M., Giralt, A., Gines, S., Canals, J. M., & Alberch, J. (2015). Fingolimod (FTY720) enhances hippocampal synaptic plasticity and memory in Huntington's disease by preventing p75NTR up-regulation and astrocyte-mediated inflammation. *Hum Mol Genet*, *24*(17), 4958-4970. https://doi.org/10.1093/hmg/ddv218

Montoya, A., Price, B. H., Menear, M., & Lepage, M. (2006). Brain imaging and cognitive dysfunctions in Huntington's disease. *J Psychiatry Neurosci*, *31*(1), 21-29. https://www.ncbi.nlm.nih.gov/pubmed/16496032
https://pmc.ncbi.nlm.nih.gov/articles/PMC1325063/

Mork, B. E., Lamerand, S. R., Zhou, S., Taylor, B. K., & Sheets, P. L. (2022). Sphingosine-1-phosphate receptor 1 agonist SEW2871 alters membrane properties of late-firing somatostatin expressing neurons in the central lateral amygdala. *Neuropharmacology*, *203*, 108885. https://doi.org/10.1016/j.neuropharm.2021.108885

Nardone, R., Lochner, P., Marth, R., Ausserer, H., Bratti, A., & Tezzon, F. (2007). Abnormal intracortical facilitation in early-stage Huntington's disease. *Clin Neurophysiol*, *118*(5), 1149-1154. https://doi.org/10.1016/j.clinph.2007.01.009

Nitta, T., Hata, M., Gotoh, S., Seo, Y., Sasaki, H., Hashimoto, N., Furuse, M., & Tsukita, S. (2003). Size-selective loosening of the blood-brain barrier in claudin-5-deficient mice. *J Cell Biol*, *161*(3), 653-660. https://doi.org/10.1083/jcb.200302070

Nitzsche, A., Poittevin, M., Benarab, A., Bonnin, P., Faraco, G., Uchida, H., Favre, J., Garcia-Bonilla, L., Garcia, M. C. L., Leger, P. L., Therond, P., Mathivet, T., Autret, G., Baudrie, V., Couty, L., Kono, M., Chevallier, A., Niazi, H., Tharaux, P. L., . . . Camerer, E. (2021). Endothelial S1P(1) Signaling Counteracts Infarct Expansion in Ischemic Stroke. *Circ Res*, *128*(3), 363-382. https://doi.org/10.1161/CIRCRESAHA.120.316711

Obermeier, B., Daneman, R., & Ransohoff, R. M. (2013). Development, maintenance and disruption of the blood-brain barrier. *Nat Med*, *19*(12), 1584-1596. https://doi.org/10.1038/nm.3407

Oztop-Cakmak, O., Solaroglu, I., & Gursoy-Ozdemir, Y. (2017). The Role of Pericytes in Neurovascular Unit: Emphasis on Stroke. *Curr Drug Targets*, *18*(12), 1386-1391. https://doi.org/10.2174/1389450117666160613104523

Padel, T., Roth, M., Gaceb, A., Li, J. Y., Bjorkqvist, M., & Paul, G. (2018). Brain pericyte activation occurs early in Huntington's disease. *Experimental Neurology*, *305*, 139-150. https://doi.org/10.1016/j.expneurol.2018.03.015

Palpagama, T. H., Waldvogel, H. J., Faull, R. L. M., & Kwakowsky, A. (2019). The Role of Microglia and Astrocytes in Huntington's Disease. *Front Mol Neurosci*, *12*, 258. https://doi.org/10.3389/fnmol.2019.00258

Pernici, C. D., Kemp, B. S., & Murray, T. A. (2019). Time course images of cellular injury and recovery in murine brain with high-resolution GRIN lens system. *Sci Rep*, *9*(1), 7946. https://doi.org/10.1038/s41598-019-44174-7

Pirhaji, L., Milani, P., Leidl, M., Curran, T., Avila-Pacheco, J., Clish, C. B., White, F. M., Saghatelian, A., & Fraenkel, E. (2016). Revealing disease-associated pathways by network integration of untargeted metabolomics. *Nat Methods*, *13*(9), 770-776. https://doi.org/10.1038/nmeth.3940

Pornnoppadol, G., Bond, L. G., Lucas, M. J., Zupancic, J. M., Kuo, Y. H., Zhang, B., Greineder, C. F., & Tessier, P. M. (2024). Bispecific antibody shuttles targeting CD98hc mediate efficient and long-lived brain delivery of IgGs. *Cell Chem Biol*, *31*(2), 361-372 e368. https://doi.org/10.1016/j.chembiol.2023.09.008

Richards, M., Nwadozi, E., Pal, S., Martinsson, P., Kaakinen, M., Gloger, M., Sjoberg, E., Koltowska, K., Betsholtz, C., Eklund, L., Nordling, S., & Claesson-Welsh, L. (2022). Claudin5 protects the peripheral endothelial barrier in an organ and vessel-type-specific manner. *Elife*, *11*. https://doi.org/10.7554/eLife.78517

Ross, C. A., Aylward, E. H., Wild, E. J., Langbehn, D. R., Long, J. D., Warner, J. H., Scahill, R. I., Leavitt, B. R., Stout, J. C., Paulsen, J. S., Reilmann, R., Unschuld, P. G., Wexler, A., Margolis, R. L., & Tabrizi, S. J. (2014). Huntington disease: natural history, biomarkers and prospects for therapeutics. *Nat Rev Neurol*, *10*(4), 204-216. https://doi.org/10.1038/nrneurol.2014.24

Sanchez, T. (2016). Sphingosine-1-Phosphate Signaling in Endothelial Disorders. *Curr Atheroscler Rep*, *18*(6), 31. https://doi.org/10.1007/s11883-016-0586-1

Sanna, M. G., Liao, J., Jo, E., Alfonso, C., Ahn, M. Y., Peterson, M. S., Webb, B., Lefebvre, S., Chun, J., Gray, N., & Rosen, H. (2004). Sphingosine 1-phosphate (S1P) receptor subtypes S1P1 and S1P3, respectively, regulate lymphocyte recirculation and heart rate. *J Biol Chem*, *279*(14), 13839-13848. https://doi.org/10.1074/jbc.M311743200

Schippling, S., Schneider, S. A., Bhatia, K. P., Munchau, A., Rothwell, J. C., Tabrizi, S. J., & Orth, M. (2009). Abnormal motor cortex excitability in preclinical and very early Huntington's disease. *Biol Psychiatry*, *65*(11), 959-965. https://doi.org/10.1016/j.biopsych.2008.12.026

Slow, E. J., van Raamsdonk, J., Rogers, D., Coleman, S. H., Graham, R. K., Deng, Y., Oh, R., Bissada, N., Hossain, S. M., Yang, Y. Z., Li, X. J., Simpson, E. M., Gutekunst, C. A., Leavitt, B. R., & Hayden, M. R. (2003). Selective striatal neuronal loss in a YAC128 mouse model of Huntington disease. *Hum Mol Genet*, *12*(13), 1555-1567. https://doi.org/10.1093/hmg/ddg169

Smith, G. A., Rocha, E. M., McLean, J. R., Hayes, M. A., Izen, S. C., Isacson, O., & Hallett, P. J. (2014). Progressive axonal transport and synaptic protein changes correlate with behavioral and neuropathological abnormalities in the heterozygous Q175 KI mouse model of Huntington's disease. *Hum Mol Genet*, *23*(17), 4510-4527. https://doi.org/10.1093/hmg/ddu166

Southwell, A. L., Franciosi, S., Villanueva, E. B., Xie, Y., Winter, L. A., Veeraraghavan, J., Jonason, A., Felczak, B., Zhang, W., Kovalik, V., Waltl, S., Hall, G., Pouladi, M. A., Smith, E. S., Bowers, W. J., Zauderer, M., & Hayden, M. R. (2015). Anti-semaphorin 4D immunotherapy ameliorates neuropathology and some cognitive impairment in the YAC128 mouse model of Huntington disease. *Neurobiol Dis*, *76*, 46-56. https://doi.org/10.1016/j.nbd.2015.01.002

Spampanato, J., Gu, X., Yang, X. W., & Mody, I. (2008). Progressive synaptic pathology of motor cortical neurons in a BAC transgenic mouse model of Huntington's disease. *Neuroscience*, *157*(3), 606-620. https://doi.org/10.1016/j.neuroscience.2008.09.020

Stamatovic, S. M., Keep, R. F., Wang, M. M., Jankovic, I., & Andjelkovic, A. V. (2009). Caveolae-mediated internalization of occludin and claudin-5 during CCL2-induced tight junction remodeling in brain endothelial cells. *J Biol Chem*, *284*(28), 19053-19066. https://doi.org/10.1074/jbc.M109.000521

Sun, G., Wang, B., Wu, X., Cheng, J., Ye, J., Wang, C., Zhu, H., & Liu, X. (2024). How do sphingosine-1-phosphate affect immune cells to resolve inflammation? *Front Immunol*, *15*, 1362459. https://doi.org/10.3389/fimmu.2024.1362459

Tan, A. Y. S., Martinez, L., Murray, H. C., Mehrabi, N. F., Tippett, L. J., Turner, C. P., Curtis, M. A., Faull, R. L. M., Dragunow, M., & Singh-Bains, M. K. (2025). Elucidating cortical neurovascular involvement in Huntington's disease using human brain tissue microarrays. *Neurobiol Dis*, *206*, 106829. https://doi.org/10.1016/j.nbd.2025.106829

Tang, B., Seredenina, T., Coppola, G., Kuhn, A., Geschwind, D. H., Luthi-Carter, R., & Thomas, E. A. (2011). Gene expression profiling of R6/2 transgenic mice with different CAG repeat lengths reveals genes associated with disease onset and progression in Huntington's disease. *Neurobiol Dis*, *42*(3), 459-467. https://doi.org/10.1016/j.nbd.2011.02.008

Tarbell, J. M. (2010). Shear stress and the endothelial transport barrier. *Cardiovasc Res*, *87*(2), 320-330. https://doi.org/10.1093/cvr/cvq146

Toman, R. E., & Spiegel, S. (2002). Lysophospholipid receptors in the nervous system. *Neurochem Res*, *27*(7-8), 619-627. https://doi.org/10.1023/a:1020219915922

Tran, C. H., & Gordon, G. R. (2015). Acute two-photon imaging of the neurovascular unit in the cortex of active mice. *Front Cell Neurosci*, *9*, 11. https://doi.org/10.3389/fncel.2015.00011

Tu, C., Rudnick, P. A., Martinez, M. Y., Cheek, K. L., Stein, S. E., Slebos, R. J., & Liebler, D. C. (2010). Depletion of abundant plasma proteins and limitations of plasma proteomics. *J Proteome Res*, *9*(10), 4982-4991. https://doi.org/10.1021/pr100646w

van Vliet, E. A., da Costa Araujo, S., Redeker, S., van Schaik, R., Aronica, E., & Gorter, J. A. (2007). Blood-brain barrier leakage may lead to progression of temporal lobe epilepsy. *Brain : a journal of neurology*, *130*(Pt 2), 521-534. https://doi.org/10.1093/brain/awl318

Vanlandewijck, M., He, L., Mae, M. A., Andrae, J., Ando, K., Del Gaudio, F., Nahar, K., Lebouvier, T., Lavina, B., Gouveia, L., Sun, Y., Raschperger, E., Rasanen, M., Zarb, Y., Mochizuki, N., Keller, A., Lendahl, U., & Betsholtz, C. (2018a). Author Correction: A molecular atlas of cell types and zonation in the brain vasculature. *Nature*, *560*(7716), E3. https://doi.org/10.1038/s41586-018-0232-x

Vanlandewijck, M., He, L., Mae, M. A., Andrae, J., Ando, K., Del Gaudio, F., Nahar, K., Lebouvier, T., Lavina, B., Gouveia, L., Sun, Y., Raschperger, E., Rasanen, M., Zarb, Y., Mochizuki, N., Keller, A., Lendahl, U., & Betsholtz, C. (2018b). A molecular atlas of cell types and zonation in the brain vasculature. *Nature*, *554*(7693), 475-480. https://doi.org/10.1038/nature25739

Verhoog, Q. P., Holtman, L., Aronica, E., & van Vliet, E. A. (2020). Astrocytes as Guardians of Neuronal Excitability: Mechanisms Underlying Epileptogenesis. *Front Neurol*, *11*, 591690. https://doi.org/10.3389/fneur.2020.591690

Villasenor, R., Lampe, J., Schwaninger, M., & Collin, L. (2019). Intracellular transport and regulation of transcytosis across the blood-brain barrier. *Cell Mol Life Sci*, *76*(6), 1081-1092. https://doi.org/10.1007/s00018-018-2982-x

Virlogeux, A., Moutaux, E., Christaller, W., Genoux, A., Bruyere, J., Fino, E., Charlot, B., Cazorla, M., & Saudou, F. (2018). Reconstituting Corticostriatal Network on-a-Chip Reveals the Contribution of the Presynaptic Compartment to Huntington's Disease. *Cell Rep*, *22*(1), 110-122. https://doi.org/10.1016/j.celrep.2017.12.013

Walker, A. G., Miller, B. R., Fritsch, J. N., Barton, S. J., & Rebec, G. V. (2008). Altered information processing in the prefrontal cortex of Huntington's disease mouse models. *J Neurosci*, *28*(36), 8973-8982. https://doi.org/10.1523/JNEUROSCI.2804-08.2008

Wang, Z., Zheng, Y., Wang, F., Zhong, J., Zhao, T., Xie, Q., Zhu, T., Ma, F., Tang, Q., Zhou, B., & Zhu, J. (2020). Mfsd2a and Spns2 are essential for sphingosine-1-phosphate transport in the formation and maintenance of the blood-brain barrier. *Sci Adv*, *6*(22), eaay8627. https://doi.org/10.1126/sciadv.aay8627

Weissberg, I., Wood, L., Kamintsky, L., Vazquez, O., Milikovsky, D. Z., Alexander, A., Oppenheim, H., Ardizzone, C., Becker, A., Frigerio, F., Vezzani, A., Buckwalter, M. S., Huguenard, J. R., Friedman, A., & Kaufer, D. (2015). Albumin induces excitatory synaptogenesis through astrocytic TGF-beta/ALK5 signaling in a model of acquired epilepsy following blood-brain barrier dysfunction. *Neurobiol Dis*, *78*, 115-125. https://doi.org/10.1016/j.nbd.2015.02.029

Wilkerson, B. A., & Argraves, K. M. (2014). The role of sphingosine-1-phosphate in endothelial barrier function. *Biochim Biophys Acta*, *1841*(10), 1403-1412. https://doi.org/10.1016/j.bbalip.2014.06.012

Wood, N. I., Carta, V., Milde, S., Skillings, E. A., McAllister, C. J., Ang, Y. L., Duguid, A., Wijesuriya, N., Afzal, S. M., Fernandes, J. X., Leong, T. W., & Morton, A. J. (2010). Responses to environmental enrichment differ with sex and genotype in a transgenic mouse model of Huntington's disease. *PLoS One*, *5*(2), e9077. https://doi.org/10.1371/journal.pone.0009077

Wood, N. I., Goodman, A. O., van der Burg, J. M., Gazeau, V., Brundin, P., Bjorkqvist, M., Petersen, A., Tabrizi, S. J., Barker, R. A., & Morton, A. J. (2008). Increased thirst and drinking in Huntington's disease and the R6/2 mouse. *Brain Res Bull*, *76*(1-2), 70-79. https://doi.org/10.1016/j.brainresbull.2007.12.007

Yang, A. C., Stevens, M. Y., Chen, M. B., Lee, D. P., Stahli, D., Gate, D., Contrepois, K., Chen, W., Iram, T., Zhang, L., Vest, R. T., Chaney, A., Lehallier, B., Olsson, N., du Bois, H., Hsieh, R., Cropper, H. C., Berdnik, D., Li, L., . . . Wyss-Coray, T. (2020). Physiological blood-brain transport is impaired with age by a shift in transcytosis. *Nature*, *583*(7816), 425-430. https://doi.org/10.1038/s41586-020-2453-z

Yang, L. X., Yao, Y. Y., Yang, J. R., Cheng, H. L., Zhu, X. J., & Zhang, Z. J. (2023). Sphingosine 1-phosphate receptor 1 regulates blood-brain barrier permeability in epileptic mice. *Neural Regen Res*, *18*(8), 1763-1769. https://doi.org/10.4103/1673-5374.360263

Yang, Q., & Cui, X. T. (2023). Advanced in vivo fluorescence microscopy of neural electronic interface. *MRS Bulletin*, *48*(5), 506-517. https://doi.org/10.1557/s43577-023-00530-7

Yocum, A. K., Yu, K., Oe, T., & Blair, I. A. (2005). Effect of immunoaffinity depletion of human serum during proteomic investigations. *J Proteome Res*, *4*(5), 1722-1731. https://doi.org/10.1021/pr0501721

Zeron, M. M., Hansson, O., Chen, N., Wellington, C. L., Leavitt, B. R., Brundin, P., Hayden, M. R., & Raymond, L. A. (2002). Increased sensitivity to N-methyl-D-aspartate receptor-mediated excitotoxicity in a mouse model of Huntington's disease. *Neuron*, *33*(6), 849-860. https://doi.org/S0896627302006153 [pii]

Zielonka, D., Marinus, J., Roos, R. A., De Michele, G., Di Donato, S., Putter, H., Marcinkowski, J., Squitieri, F., Bentivoglio, A. R., & Landwehrmeyer, G. B. (2013). The influence of gender on phenotype and disease progression in patients with Huntington's disease. *Parkinsonism Relat Disord*, *19*(2), 192-197. https://doi.org/10.1016/j.parkreldis.2012.09.012

Zielonka, D., & Stawinska-Witoszynska, B. (2020). Gender Differences in Non-sex Linked Disorders: Insights From Huntington's Disease. *Front Neurol*, *11*, 571. https://doi.org/10.3389/fneur.2020.00571

REVISION #2

Dear Reviewers,

We again sincerely thank you for careful evaluation of our manuscript, and for thoughtful, constructive, and insightful comments.

All the remaining responses and correction in the manuscript have been color-coded in blue for readability.

Summary of additions and clarifications (revision #2):

- We added the additional study limitation requested by **Reviewer 3**.

- During the revision, exploratory data analysis identified an additional finding that we considered may be relevant to the study and have therefore included one figure panel in the revised manuscript.

- As the revised manuscript remained below the maximum of 10 main-text display items, we moved the graphical summary of the findings from the Supplementary Information to the main manuscript. Accordingly, the former Supplementary Figure 9 is now Figure 7.

- We also made targeted revisions throughout the manuscript to clarify analytical procedures, update the figures (including online deposition of colorblind-friendly versions) and legends, to improve reproducibility and facilitate evaluation of the study. Additional corrections were made to comply with the Author Checklist, including shortening the manuscript title, the manuscript section order, Author Contributions and Competing Interests statements. All revisions and newly added sections are marked in blue.

Below, we list the detailed response to the remaining Reviewer's comments:

Krzysztof Kucharz and
Martin Lauritzen

on behalf of all authors

REVIEWER #1

Reviewer #1 (Remarks to the Author):
The authors have done a very good job addressing the reviewer comments in a constructive and substantive manner. Importantly, they went beyond clarification and added new experimental data (e.g., claudin-5 IHC and expanded human analyses), which strengthens the core conclusions. The revised manuscript is clearer, better contextualized within the existing literature, and more balanced in its interpretation of the findings. While some mechanistic aspects remain necessarily correlative, the authors now appropriately acknowledge these limitations.

Overall, the revision significantly improves the quality and impact of the study.

We thank Reviewer #1 for positive assessment of the revised manuscript, his/her kind words, appreciation of our work and insightful comments.

REVIEWER #3

Reviewer #3 (Remarks to the Author):

In the current revised version, the authors have made an effort to improve the manuscript and to reply to most of our points. The work is interesting, methodologically sound for the most part, and extensive.
However, one point remains unsatisfactory. Specifically, concerning the authors' claim that the use of an open cranial window is the "gold standard" for reaching deeper vessels, we politely disagree. We are aware—from our own experimental work and research focus—of the various imaging preparations used for CNS vasculature and their effects on the tissue. The authors' "gold standard" argument for the open cranial window is misleading. It is true that a closed cranial window does introduce optical scattering; however, such closed cranial preparations routinely allow imaging at depths well beyond 50 µm—which is consistent with what the cited literature reports—and are widely used to resolve structures such as small as dendritic spines, structures far below the scale of what the authors aim to measure (tracer extravasation). As we clarified in our original comment, an already inflamed model such as R6/2 could be considerably more susceptible to surgical artifacts than a healthy control. Similarly, the reversibility with SEW2871 is not conclusive: it could very well act on surgical inflammation rather than on the underlying pathological process. The use of an open cranial window, in the absence of further confirmation techniques, is therefore a methodological limitation of the study.

We are aware that performing further experiments to corroborate this finding may be challenging. A reasonable proposition would therefore be for the authors to mention this limitation in the text and to suggest the use of closed cranial window preparations or alternative experimental approaches which do not introduce experimental artefacts (e.g., histology) in future work.

We thank the Reviewer for returning to this important methodological issue and agree that describing the open cranial window as the "gold standard" was unnecessarily categorical.

Although tissue perturbation is not unique to two-photon microscopy and is inherent, to varying degrees, to most experimental techniques, we acknowledge the possibility that craniotomy and dura removal might influence local vascular or inflammatory conditions and may affect R6/2 and WT brains differently. Unfortunately, this genotype-dependent potential susceptibility for microsurgery cannot be isolated using a fully equivalent control because the same cranial access is required for the measurements.

While the distinct abnormalities observed in neighboring vessel types exposed to the same surgical environment, the vessel-selective effects of SEW2871, and the concordant claudin-5 and astrocyte findings obtained independently in mouse and human histology argue against the phenotype being explained solely by a nonspecific craniotomy effect, this possibility cannot be completely excluded.

We have therefore added this limitation to the Discussion and identified closed-window or thinned-skull imaging, as important directions for future validation.

> *"(…) Lastly, it cannot be excluded that craniotomy and dura removal might alter local vascular or inflammatory conditions differently in R6/2 and WT brains, and the use of e.g., thinned-skull imaging, or closed cranial window preparations, or alternative experimental imaging approaches could mitigate this potential issue. However, the arteriolar predominance of the phenotype, rather than a stronger venular involvement expected from an inflammatory response, together with concordant mouse and human histology and genotype- and treatment-dependent proteomic signatures, argues against the findings being solely attributable to the microsurgical preparation for imaging."*

*This information has now been added to the Methodological considerations/limitations of the study [**Page 26**].*

REVIEWER #4

Reviewer #4 (Remarks to the Author):
I co-reviewed this manuscript with one of the Reviewers who provided the listed reports. This is part of the Nature Communications initiative to facilitate training in peer review and to provide appropriate recognition for Early Career Researchers who co-review manuscripts.

We thank Reviewer #4 for co-reviewing the manuscript and for valuable contribution to its evaluation.

REVIEWER #5

Reviewer #5 (Remarks to the Author):

I would like to thank the authors for their efforts in revising the manuscript and addressing all my comments.

All my concerns have been satisfactorily answered, exceeding my expectations.

The manuscript is now excellent, and I have no further comments.

We sincerely thank Reviewer #5 for the very positive assessment of the revision and kind words. We greatly appreciate the Reviewer's careful evaluation and are pleased that all concerns have been satisfactorily addressed.

ADDITIONAL FINDING

We reported that CAG repeat length did not correlate with gross GFAP+ astrocyte density measured in human samples (Supplementary Fig. 8d). However, a complementary finding emerged from our exploratory analysis of already generated data during the revision process.

> Using correlation analysis, our data shows that the CAG length in patients may strongly predict arteriolar polarization in pathology, i.e. how much arterioles are affected compared to venules within each individual. Specifically, the periarteriolar-to-perivenular GFAP+ ratio (arteriolar GFAP+ density / venular GFAP+ density) increased with CAG repeat length (Fig. 6h; Supplementary Data 7).

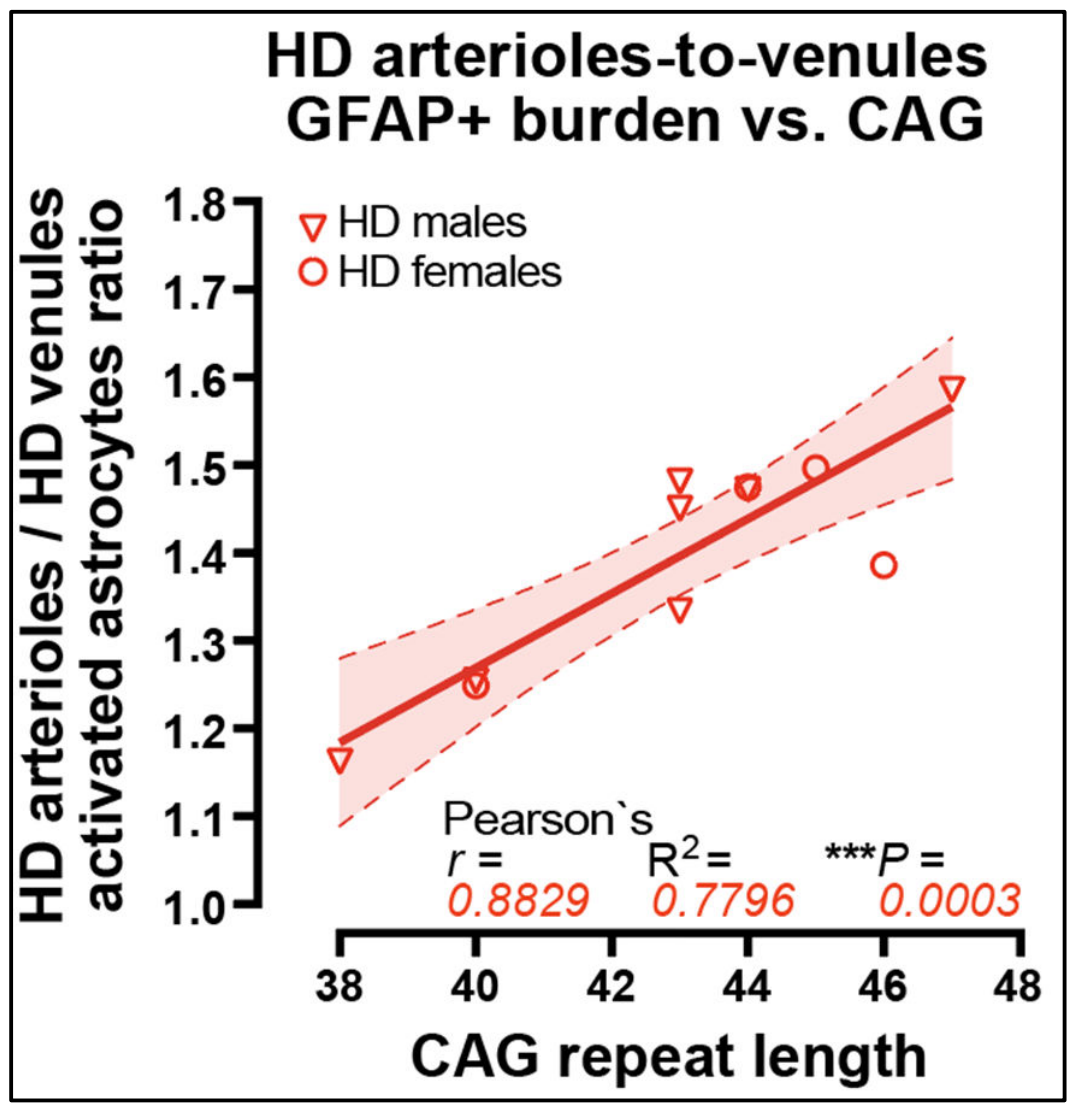

Figure 6h Pearson's correlation analysis showing that the arteriovenous asymmetry of astroglial activation increases with CAG repeat length, with progressively greater periarteriolar GFAP+ astrocyte activation relative to perivenular activation. *r*, Pearson's correlation coefficient; R^2, coefficient of determination; *p*, two-sided probability under the null hypothesis of no linear correlation. The line and shaded area indicate the linear regression fit and 95% confidence interval, respectively. Each point represents one HD case.

While this complementary finding does not alter any of the manuscript conclusions, we considered it to be appropriate to include it to results section. This panel has now been included as Fig 6h, and is mentioned in the Results section (Page 8).
