# Supplementary material for: Huntington’s disease vasculopathy is barrier-type-selective and arteriolar-dominant with spatially-coupled astrogliosis and reversible by S1pr1 stimulation
Source: Nat Commun. 2026 Sep 12;17:10775. doi: 10.1038/s41467-026-77474-4 (PMC13661524; doi:10.1038/s41467-026-77474-4)
Supplement: Supplementary file 3 [file 41467_2026_77474_MOESM1_ESM.pdf]

SUPPLEMENTARY INFORMATION

TITLE:

Huntington's disease vasculopathy is barrier-type-selective and arteriolar-dominant with spatially-coupled astrogliosis and reversible by S1PR1 stimulation

AUTHORS:

Krzysztof Kucharz[#1], Rana Soylu-Kucharz[1,2], Martine Saint-Pierre[3,4], Melanie Alpaugh[5], Filippa Liliendahl Qvist[6], Maria Björkqvist[2], Niels Henning Skotte[6,7], Francesca Cicchetti[3,4], Martin Johannes Lauritzen[1]

[1] Department of Neuroscience, University of Copenhagen, Copenhagen, Denmark,
[2] Department of Experimental Medical Science, Lund University, Lund, Sweden,
[3] Centre de Recherche du CHU de Québec, Axe Neurosciences, Québec, Canada,
[4]Département de Psychiatrie & Neurosciences, Université Laval, Québec, Canada,
[5] Department of Molecular and Cellular Biology, University of Guelph, Canada,
[6] Department of Drug Design and Pharmacology, University of Copenhagen, Denmark,
[7] NNF Center for Protein Research, Faculty of Health Sciences, University of Copenhagen, Denmark

[#]**Correspondence:** Krzysztof Kucharz (kucharz@sund.ku.dk);

CONTENTS:

Supplementary Figures (Pages **2-12**)
Supplementary Notes (Pages **13-15**)
Supplementary References (Pages **16-17**)

SUPPLEMENTARY FIGURES

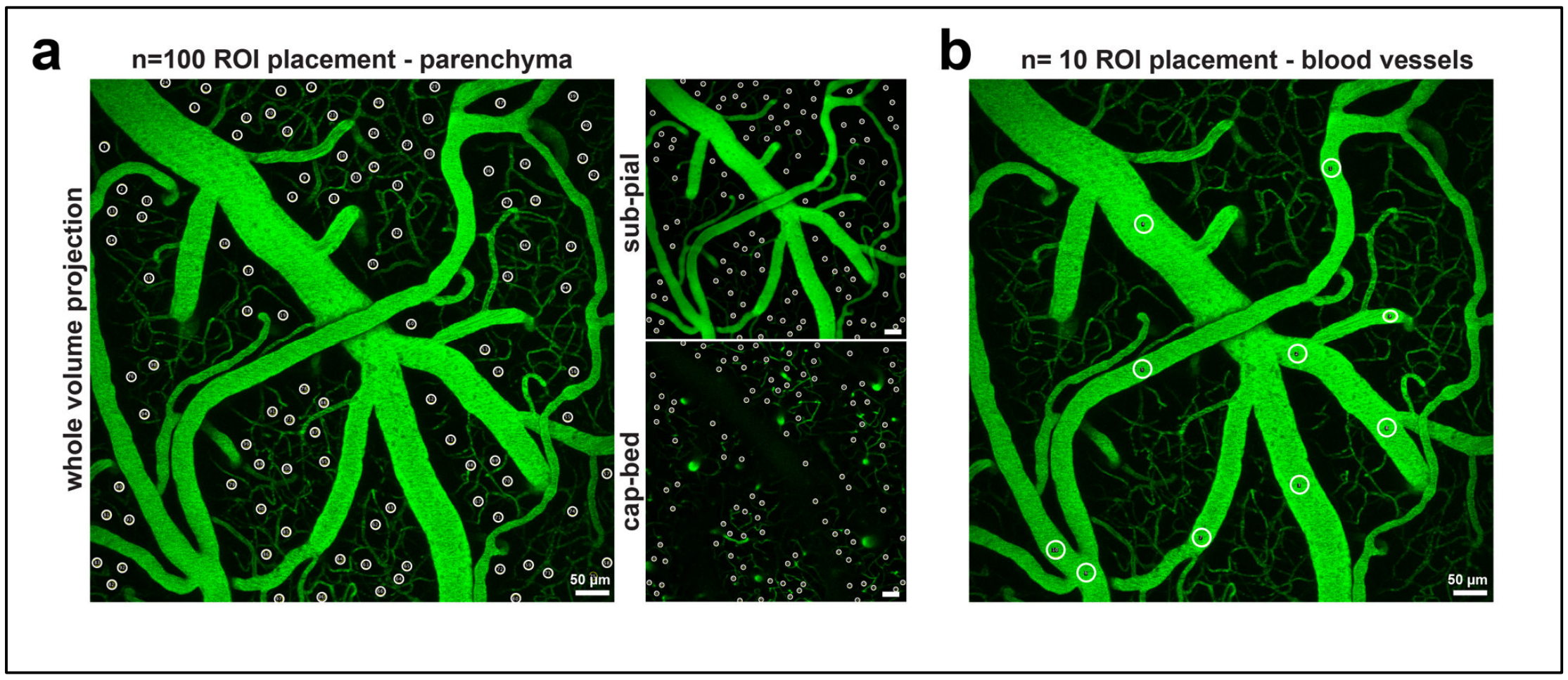

Supplementary Fig. 1. Placement of regions of interest (ROIs) to measure paracellular permeability using 2PM *in vivo.* a Placement of ROIs in the brain parenchyma (pROIs, white circles) to measure the NaFluo signal increase in the brain. The pROIs are placed in areas devoid of vessels, using as an anatomical reference the maximum intensity projection across the whole analyzed volume (left panel). Next, the same set of pROIs is reused for collecting the signal from *sub-pial* and capillary bed *(cap-bed)* volumes of the cortex**. b** Placement of the vessel ROIs (vROIs, white circles) to obtain the mean signal of NaFluo circulating in the bloodstream. n_{pROI} = 100 / animal, n_{vROI} = 10 / animal; in n_{WT}= 9; $n_{R6/2}$= 7; $n_{R6/2+}$= 7 mice.

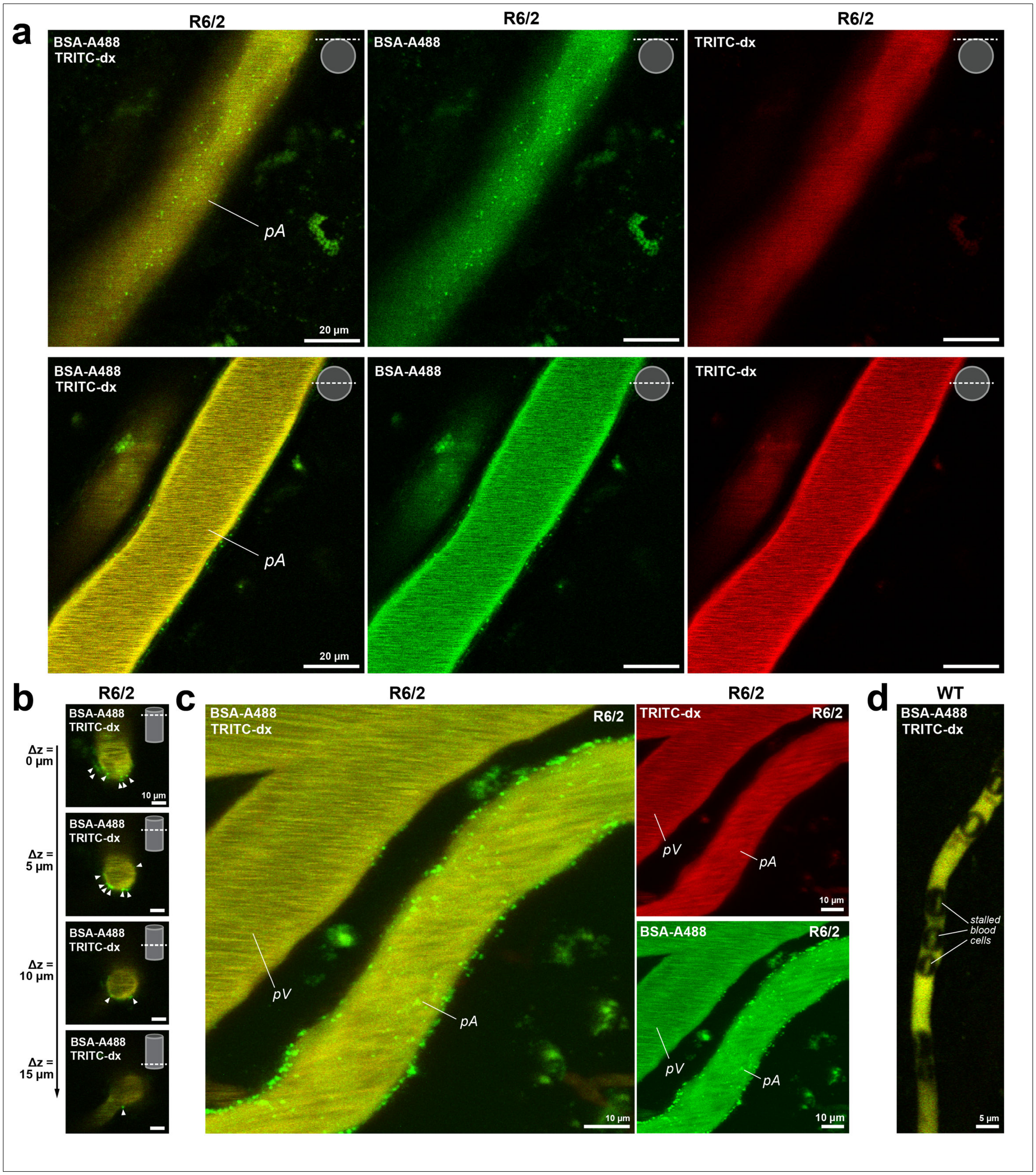

Supplementary Fig. 2. 2PM examples of AMT imaging in vivo. **a** Example images of AMT puncta (BSA-A488, green) imaged in a pial arteriole. The upper panels show an arteriole with an imaging focal plane at its pial surface; the lower panels show the same arteriole with the imaging focal plane crossing the broadest section of the vessel. The inserts in the upper-right corner show the location of the focal plane in relation to the vessel cross-section. **b** Example images of AMT puncta measured along the penetrating arteriole, with the inset showing the location of the focal plane along the vertically oriented vessel and Δz showing the relative depths of the focal plane. **c** Neighboring arteriole and venule demonstrate contrast in vulnerability (AMT puncta extent) in R6/2 mice. **d** Vessels with stalled blood flow, i.e., increased interaction time with albumin and BECs, do not exhibit AMT increase. The image shows an example of a stalled capillary in WT mice, with numerous immobile blood cells obstructing the blood flow and the absence of AMT puncta.

All panels: *pA* = pial arteriole, *pV* = pial venule.

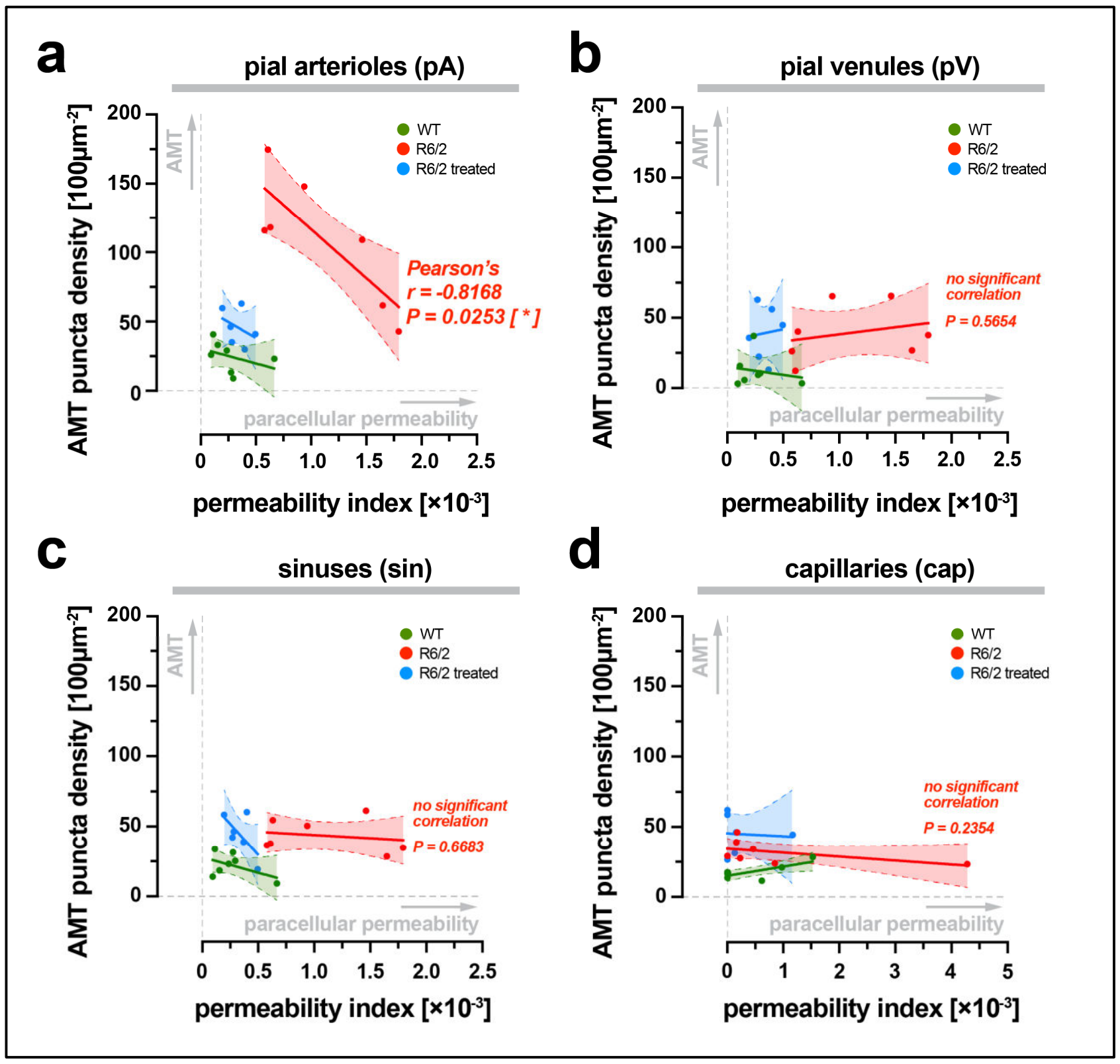

Supplementary Fig. 3. Either AMT or paracellular leakage pathology takes precedence at pial arterioles in R6/2 HD mice. Cross-correlation between the degree of paracellular permeability and AMT in WT, R6/2, and R6/2 treated mice (R6/2+). **a** In R6/2 pial arterioles (pA), the degree of AMT and paracellular permeability increases were inversely correlated (Pearson's r=-0.8168; **P=0.0253*). **b-d** This relation was not present in other vessel types occupying the same niche, and capillaries.

All panels: Each point represents a single animal; number of pairs: n_{WT}= 7; $n_{R6/2}$= 7; $n_{R6/2+}$= 6 mice; *r* = Pearson's correlation coefficient; *P* = two-sided probability under the null hypothesis of no linear correlation. The thick lines show a linear fit with a shaded area representing a 90% confidence interval. *AMT puncta density* = measure of the extent of AMT; Gray arrows denote the direction of increases in AMT and paracellular permeability. Source data are provided with this paper.

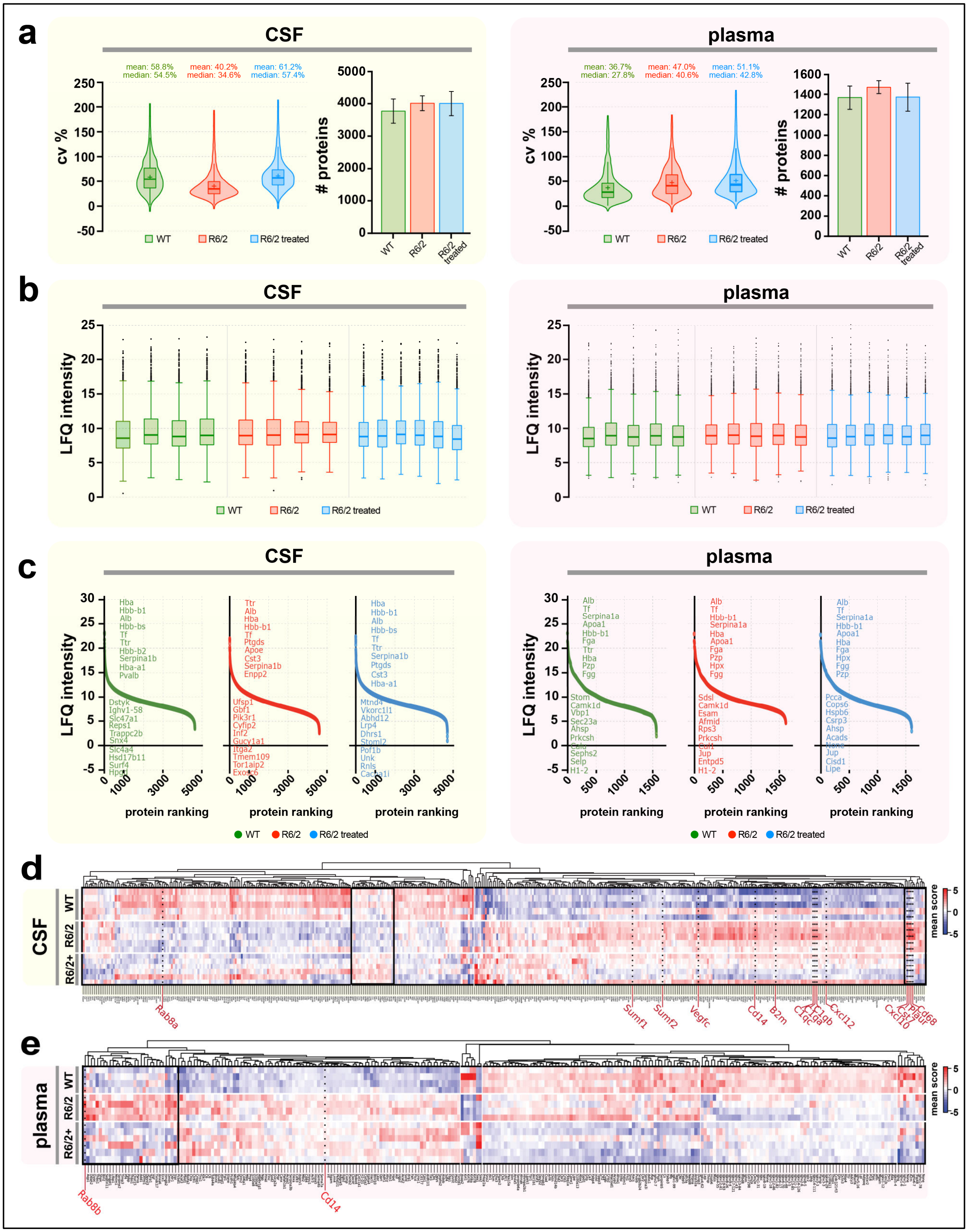

Supplementary Fig. 4. Protein yield and quality control of blood plasma and cerebrospinal fluid samples for mass spectrometry analysis. a The average number of proteins identified and the coefficient of variation (cv) are illustrated by group for both plasma and CSF. **b** Normalized protein intensities across all samples are depicted using box plots for both plasma and CSF to visualize the protein distribution. **c** The ranked proteins based on intensity are shown to illustrate the dynamic range and the top 10 highest and lowest expressed proteins by mass spectrometry. **d-e** CSF and plasma cluster analysis based on significant

hits demonstrating the grouping of the replicate samples. The genotypic and treatment effects can be observed in black boxes, and highlighted proteins are of direct relevance to BCSFB/BBB function.

All panels: n_{WT}= 5; $n_{R6/2}$= 5; $n_{R6/2+}$= 6 mice. Two mice were excluded from plasma analysis due to low protein yield and protein identifications. Source data are provided with this paper.

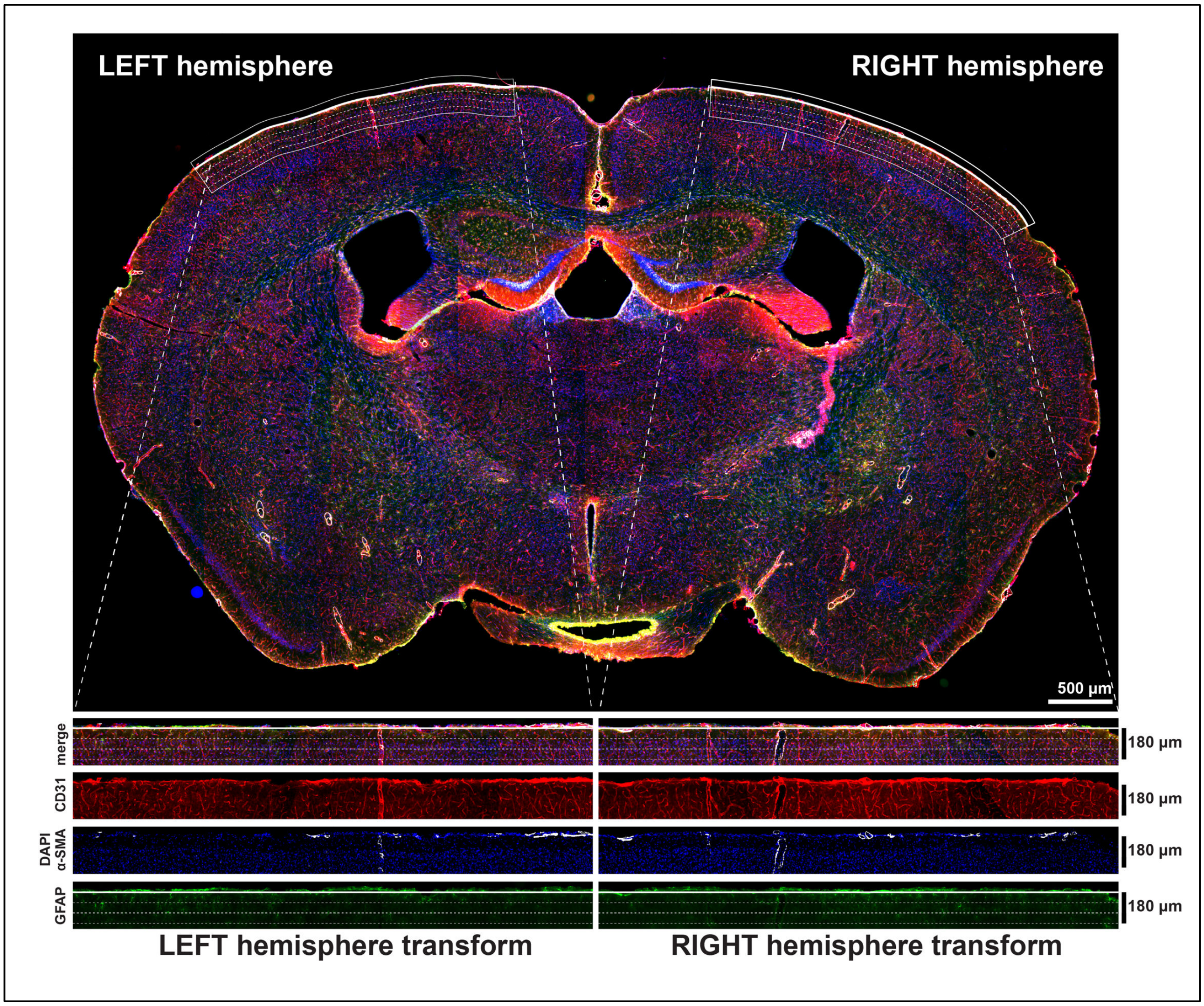

Supplementary Fig. 5. Mouse GFAP immunohistochemistry. Representative immunofluorescence image from a whole coronal brain section. The superficial cortical ribbon of each hemisphere was straightened by non-rigid deformation to align the pial surface and quantify GFAP+ astrocytes across three consecutive 60-µm depth intervals. Dashed lines mark interval boundaries; the bold line indicates the pia.

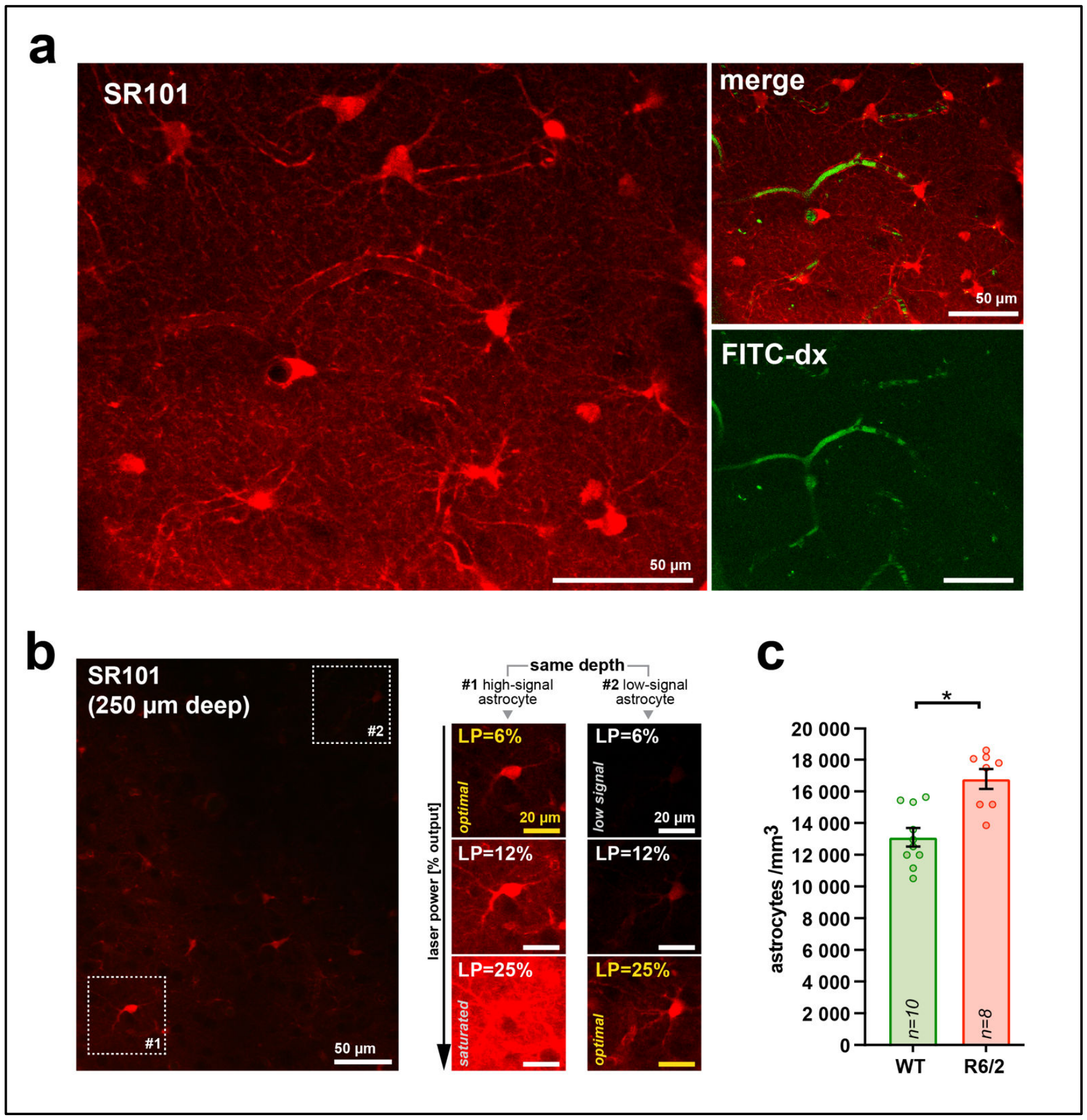

Supplementary Fig. 6. Measurements of astrocyte density *in vivo.* **a** Example images of astrocytes labeled *in vivo* with an intraparenchymal injection of SR101, a pan-astrocytic fluorescent marker. **b** Due to the varying extent of SR101 uptake by astrocytes, the images were collected with increasing laser intensity (here: 6-25% of the output power 2.51 W measured at the laser) to obtain the optimal detection range for all, both high-signal and low-signal astrocytes. **c** Compared to WT, R6/2 mice exhibit increased astrocyte density in cortical parenchyma. n_{WT}= 10; $n_{R6/2}$ = 8. Data is shown as average ± SEM; two-tailed t-test; *=p<0.05. Source data are provided with this paper.

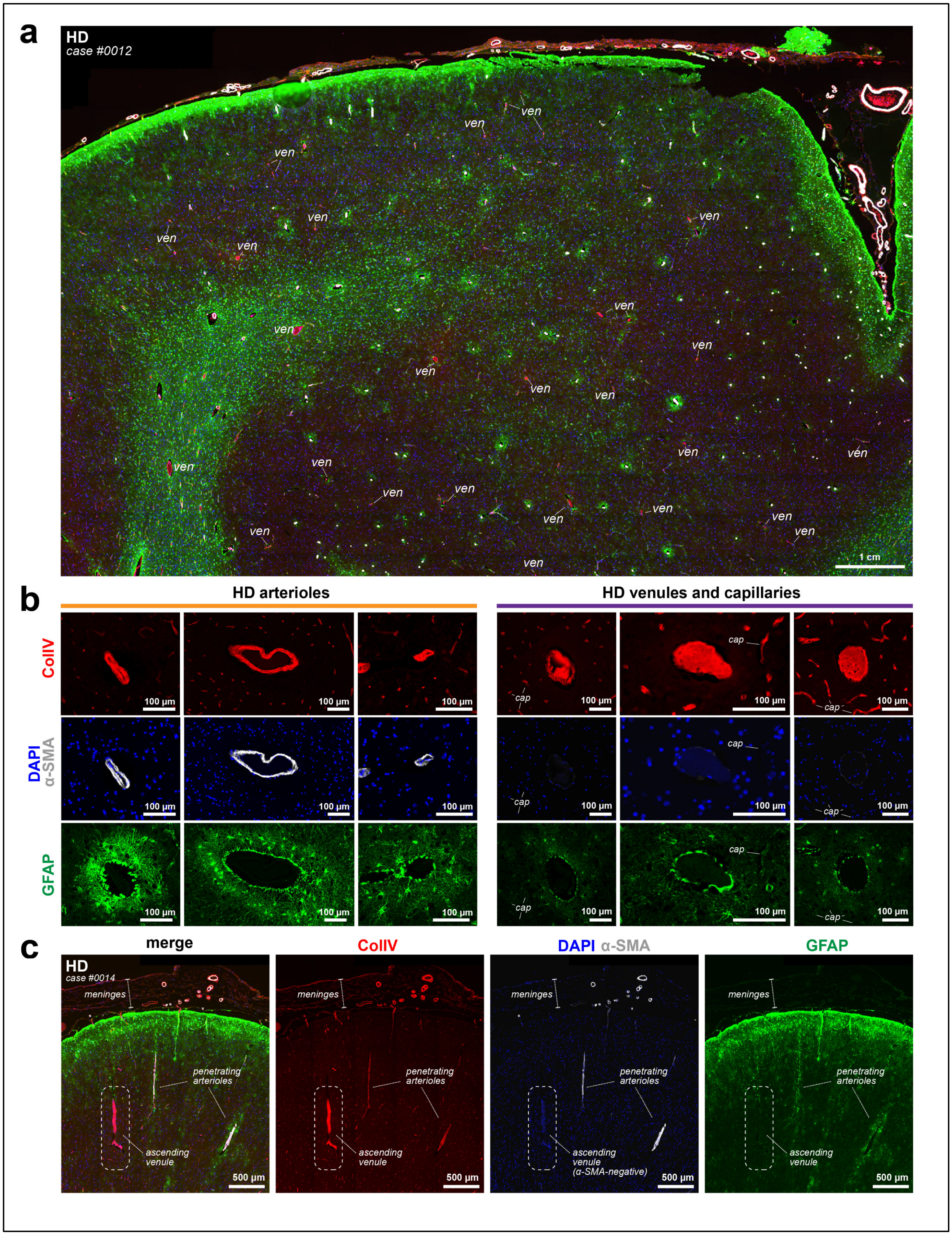

Supplementary Fig. 7. Immunohistochemistry on *post-mortem* human HD brains. a Immunofluorescence image of the cortical region from HD brain depicting a dense network of arterioles and venules within the field of view. The image demonstrates preferential astrocyte enrichment in proximity to arterioles, with minimal association observed in venules. For clarity, only vessels identified as venules are labeled (*ven*), while all unlabeled large vessels correspond to arterioles. **b** Representative images from Fig. 6d presented as individual fluorescence channels. GFAP+ astrocytes exhibit a distinct perivascular halo surrounding

arterioles, while capillaries (*cap*) and venules show only minimal astrocytic activation. **(C)** The spatial pattern of astrocyte activation is preserved regardless of vessel orientation. Sparse penetrating arterioles and ascending venules maintain the same trend, with astrocyte enrichment preferentially localized around arterioles.

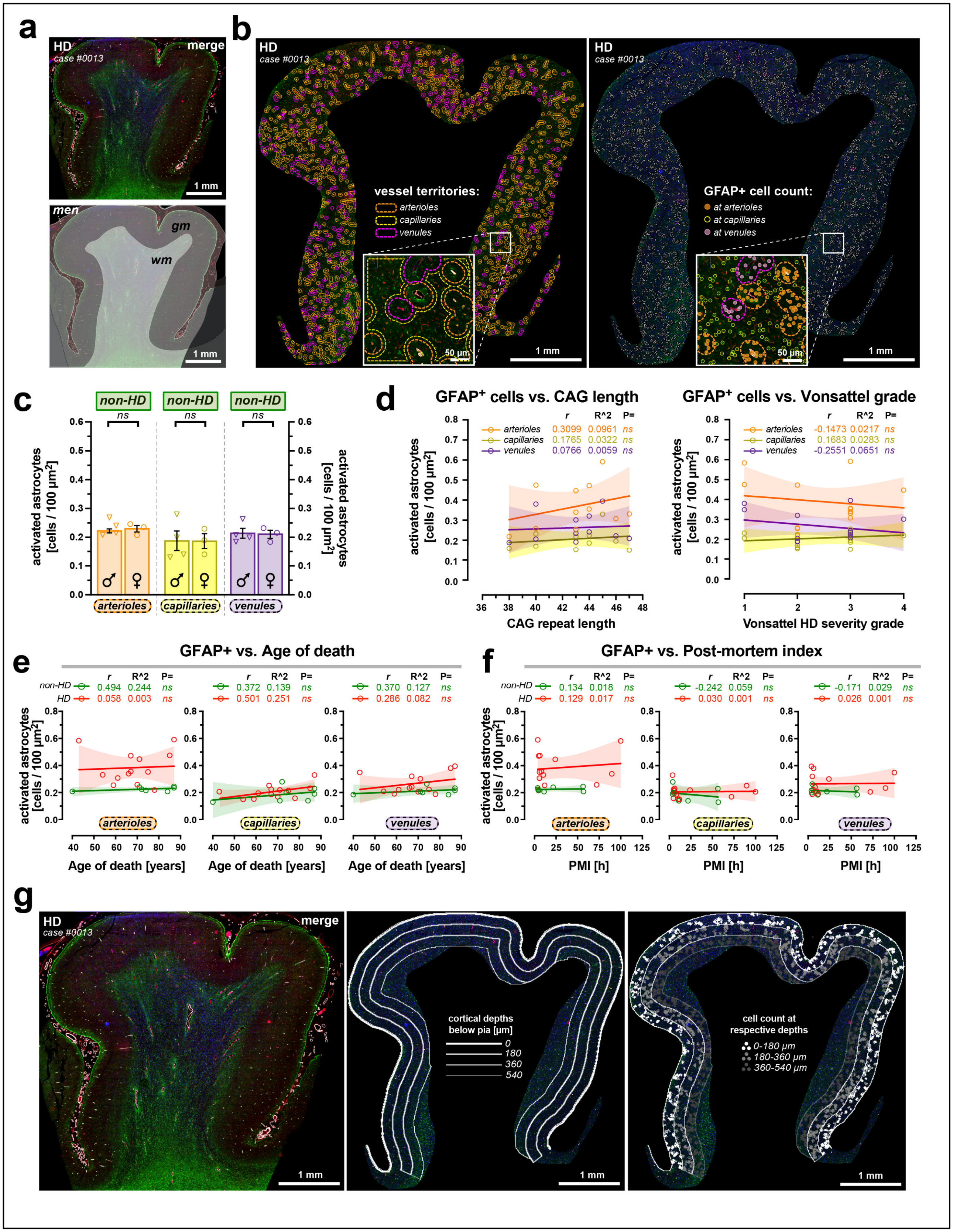

Supplementary Fig. 8. Quantitative analyses of human *post-mortem* brain sections. a Immunofluorescence image of the cortical region from an HD human brain with a schematic representation of anatomical features. **b** Gray matter panels corresponding to the brain region shown in panel (a), delineating arterial (orange), capillary (yellow), and venular (purple) territories based on a 20 µm cutoff. GFAP+ astrocytes were quantified within each territory, with astrocytes located in overlapping regions excluded from both arteriole and venule counts. **c** Comparison of the density of GFAP+ astrocytes in non-HD human brains at

distinct vessel types in males and females. The density of activated astrocytes is independent of sex and remains comparable across all vessel types. **d** Correlation analyses showing that in HD human brains, the density of activated astrocytes is not correlated with donor CAG repeat length or Vonsattel's grade of disease severity. Bold lines are linear fit, shaded areas show 90% confidence interval. **e-f** Internal controls demonstrating that GFAP+ astrocyte density across all vessel types is unaffected by the donor's age at death or post-mortem tissue interval. **g** Panels illustrating the same cortical region as in panel (a), depicting the analysis of the astrocytic activation gradient in relation to cortical depth.

All panels: Each point represents a single donor case, $n_{non\text{-}HD}$= 7 (4 males, 3 females); n_{HD}= 13 cases (8 males, 5 females). The thick lines show a linear fit with a shaded area representing a 90% confidence interval. *r* = Pearson's correlation coefficient; R^2, coefficient of determination; *P*= two-sided probability under the null hypothesis of no linear correlation. Bar graphs: data is shown as average ± SEM; two-tailed t-test; *ns*=non-significant. Source data are provided with this paper.

SUPPLEMENTARY NOTES

Consideration of alternative HD models

The R6/2 model of HD is the only HD mouse model in which BBB defects have been consistently demonstrated in contrast to BCSFB/BBB HD phenotypes absent in other HD mouse models (YAC128, BACHD, knock-in). Di Pardo et al. showed that R6/2 mice develop significant BBB leakage at symptom onset (~6–8 weeks), far earlier than previously assumed, starting at early disease (6 weeks), and worsening by the late stage (12 weeks) (Di Pardo et al., 2017). This indicates that the breach of BBB integrity, preceding or coincident with motor symptom onset in R6/2 mice (Di Pardo et al., 2017). Our current data agree with this: we showed ~4-fold higher paracellular tracer leakage in cortical microvessels (particularly surface vessels) in R6/2 compared to WT controls. Notably, the R6/2 model reproduces the BBB breakdown seen in HD, and does so in a spatially relevant manner (affecting cortex and striatum, the same regions as in HD patients) (Drouin-Ouellet et al., 2015).

Although distinct HD models have their merits, we concluded that no well-established models would allow us to interrogate late-stage cortical BBB pathology as readily as R6/2:

Model	Cortical Pathology (Atrophy, Gliosis, Synaptic Loss)	BCSFB/BBB Disruption
**R6/2** *(Exon-1)*	Severe, early-onset; robust atrophy and gliosis (Etxeberria-Rekalde et al., 2020).	**YES**. Significant leakage and AMT disinhibition (Di Pardo et al., 2017).
**YAC128** *(Full)*	Late-onset; atrophy visible by 9–12 months (Slow et al., 2003).	**NO**. BBB is intact under normal conditions, but can be breached by systemic LPS administration (Franciosi et al., 2012).
**BACHD** *(Full)*	Gradual; significant atrophy only after 12 months (Spampanato et al., 2008).	**NO.** Integrity maintained throughout life, normal cerebrovascular function (Mantovani et al., 2016).
**Knock-in** *(Q140/150/Q175)*	Typically, very slow; only subtle changes until 12–22 months (Bayram-Weston et al., 2012; Smith et al., 2014).	**NO(?)**. No reported leakage. Due to late onset age, possibility of interference of aging processes on BCSFB/BBB for baseline recordings (Yang et al., 2020).
**Human HD** *(ground truth)*	Hallmark cortical thinning and neurodegeneration.	**YES.** Documented leakage and vascular changes (Drouin-Ouellet et al., 2015).

Specifically:

1) **YAC128** (full-length human HTT, ~128 CAG) model reproduces many HD features over a prolonged timescale (12–18 months). However, BBB disruption is minimal in YAC128, especially at early stages. In baseline conditions, young YAC128 mice show no significant tracer leakage; even at 12 months, only subtle vascular changes are noted (e.g., slightly increased microvessel density and reduced diameter) with intact barrier function (Franciosi et al., 2012). These mice require an external insult (chronic LPS inflammation) to unmask BBB leakage–LPS–triggered striatal fibrinogen extravasation and vessel wall thickening, while untreated YAC128 mice did not exhibit increased BBB permeability (Franciosi et al., 2012). Moreover, to reach a comparable disease stage to R6/2, YAC128 mice would need to be ~12–15 months old, which presents practical challenges for two-photon imaging.

2) **BACHD** (full-length HTT BAC, ~97 CAG): BACHD mice express mutant huntingtin ubiquitously and show progressive motor impairments starting at ~3 months (Mantovani et al., 2016). However, their neuropathology is notably mild through mid-life, and BACHD mice have normal striatal neuron counts at 12–15 months, with no signs of BBB degeneration, even in 12-month-old mice. Given the absence of overt neurodegeneration or inflammatory pathology at the ages typically studied, the BBB in BACHD is presumed to be intact (Mantovani et al., 2016), with authors explicitly cautioning against the use of this model for preclinical studies in HD.
3) **Knock-in models** (HdhQ140, HdhQ150, zQ175, etc.): Knock-in (KI) mice have the mutant CAG expanded in the native mouse Htt gene, preserving endogenous regulation. They are genetically precise but slow-progressing, and to our knowledge, there is no evidence of spontaneous BBB leakage in Q140 or Q175 mice even at 1+ year of age, consistent with their milder HD pathology (Menalled, 2005; Smith et al., 2014). As such, detecting and imaging BBB dysfunction in them would be impractical, having to age cohorts to 18–24 months, and even then, the changes might be too subtle. At the same time, the late onset age would not only dramatically increase the risk of mortality-related surgical procedures and imaging anesthesia, but also be confounded by the presence of deleterious aging processes that gradually compromise BCSFB/BBB (Yang et al., 2020). Therefore, using KI mice would not meet our experimental need for a robust, measurable BBB phenotype in a reasonable timeframe.

Beyond gross leakage, R6/2 mice exhibit a rich spectrum of neurovascular pathology that closely mirrors human HD. For example, R6/2 brains have aberrant microvascular remodeling – increased vessel density but reduced vessel diameter – suggestive of pro-angiogenic yet maladaptive changes (Drouin-Ouellet et al., 2015) and mHTT aggregates deposit in blood vessels and perivascular cells of R6/2 mice, as in HD patients (Drouin-Ouellet et al., 2015). Notably, we have previously found mHTT in all major cellular components of the neurovascular unit, accompanied by substantial BBB leakage in the striatum (Drouin-Ouellet et al., 2015), and confirmed similar vascular and BBB changes in human HD post-mortem brains, firmly linking the R6/2 vascular phenotype to the human condition changes (Drouin-Ouellet et al., 2015).
Overall, alternative models either lack substantial cortical BBB disruption (YAC128, BACHD, KI) or require very long durations to manifest any BBB phenotype. By contrast, the R6/2 model – including our high-CAG variant, exhibits early, robust BBB dysfunction, closely paralleling human HD.

Rationale for using the long-CAG variant of the R6/2 model of HD

A key benefit of using the high-CAG R6/2 line is its prolonged disease course. There exists a well-documented, paradoxical relationship between CAG length and disease progression. R6/2 mice with extremely expanded CAG tracts (>300) have a slower progression and extended lifespan compared to the conventional ~150 CAG R6/2 mice (Cummings et al., 2012; Tang et al., 2011). This phenomenon is well documented: increasing the R6/2 CAG from ~110 to 150 accelerates disease, but further expansion to ~210–310 ameliorates severity and delays onset (Cummings

et al., 2012). For instance, Tang et al. reported that R6/2 mice carrying ~300 CAGs live longer and develop symptoms more gradually than the ~150 CAG line (Tang et al., 2011). This allowed us to perform technically demanding in vivo two-photon imaging at advanced disease stages, which would be infeasible in standard R6/2 mice that typically reach endpoint by ~12-14 weeks. Despite the extended timeline, the high-CAG R6/2 model retains a robust HD-like phenotype, encompassing the hallmark neuropathological and behavioral deficits of HD. Like conventional R6/2 mice, the >300 CAG lines show progressive brain atrophy (striatum and cortex), weight loss, and motor dysfunction (Etxeberria-Rekalde et al., 2020). Notably, significant cortical and striatal neurodegeneration with gliosis and huntingtin aggregation (Etxeberria-Rekalde et al., 2020), classic features of HD, are still present in the long-CAG line, albeit on a delayed schedule. For example, Tang et al. observed that high-CAG R6/2 mice form mutant huntingtin (mHTT) inclusions and transcriptional changes similar to the ~150 CAG line, but with later onset (Tang et al., 2011). Interestingly, the ultra-long CAG expansions can alter the subcellular pathology, i.e., R6/2^{Q300} mice tend to accumulate more small cytoplasmic aggregates (versus large nuclear inclusions in R6/2^{Q150}) (Tang et al., 2011), yet they ultimately develop the same pathogenic endpoints (synaptic dysfunction, neuronal loss). Importantly, key HD pathological hallmarks are robustly expressed in this model. By 14–16 weeks, long-CAG R6/2 mice exhibit: (i) neuroinflammation and gliosis: increased GFAP$^+$ astrocytes and activated microglia paralleling early neuronal injury (Etxeberria-Rekalde et al., 2020); (ii) pronounced cortical atrophy and neuron loss, akin to human HD where cortex and striatum undergo progressive degeneration (Etxeberria-Rekalde et al., 2020; Southwell et al., 2015); (iii) synaptic and electrophysiological dysfunction, including cortical hyperexcitability and impaired corticostriatal transmission, as shown in R6/2 lines of various CAG lengths (Barry et al., 2022; Cummings et al., 2012); and (iv) mHTT aggregation and ubiquitination throughout the brain (Etxeberria-Rekalde et al., 2020). These outcomes reinforce that the long-CAG R6/2 mice still manifest a robust and relevant HD phenotype.

Notably, the "slowed-down" R6/2 phenotype also provided a more stable and sufficiently wide experimental window (~2.5 weeks) to perform complex two-photon imaging. This technique has low throughput, yielding one animal imaging session per day. Using slow-progressing high-CAG R6/2 is a feat that allows imaging that would be technically unfeasible in more aggressive lines where mice undergo rapid decline and imminent mortality. This extended trajectory enabled the experiments to be conducted across all experimental groups over ~2 weeks without confounding disease stage, i.e., in each group and across the groups, the first imaged mouse was not systematically healthier than the last. As a result, we could treat the entire cohort as being at approximately the same disease timepoint, and perform comparisons between the groups (WT vs. R6/2 vs. R6/2 treated mice).

SUPPLEMENTARY REFERENCES

Barry, J., Bui, M. T. N., Levine, M. S., & Cepeda, C. (2022). Synaptic pathology in Huntington's disease: Beyond the corticostriatal pathway. *Neurobiol Dis*, *162*, 105574. https://doi.org/10.1016/j.nbd.2021.105574

Bayram-Weston, Z., Torres, E. M., Jones, L., Dunnett, S. B., & Brooks, S. P. (2012). Light and electron microscopic characterization of the evolution of cellular pathology in the Hdh(CAG)150 Huntington's disease knock-in mouse. *Brain Res Bull*, *88*(2-3), 189-198. https://doi.org/10.1016/j.brainresbull.2011.03.014

Cummings, D. M., Alaghband, Y., Hickey, M. A., Joshi, P. R., Hong, S. C., Zhu, C., Ando, T. K., Andre, V. M., Cepeda, C., Watson, J. B., & Levine, M. S. (2012). A critical window of CAG repeat-length correlates with phenotype severity in the R6/2 mouse model of Huntington's disease. *J Neurophysiol*, *107*(2), 677-691. https://doi.org/10.1152/jn.00762.2011

Di Pardo, A., Amico, E., Scalabri, F., Pepe, G., Castaldo, S., Elifani, F., Capocci, L., De Sanctis, C., Comerci, L., Pompeo, F., D'Esposito, M., Filosa, S., Crispi, S., & Maglione, V. (2017). Impairment of blood-brain barrier is an early event in R6/2 mouse model of Huntington Disease. *Sci Rep*, *7*, 41316. https://doi.org/10.1038/srep41316

Drouin-Ouellet, J., Sawiak, S. J., Cisbani, G., Lagace, M., Kuan, W. L., Saint-Pierre, M., Dury, R. J., Alata, W., St-Amour, I., Mason, S. L., Calon, F., Lacroix, S., Gowland, P. A., Francis, S. T., Barker, R. A., & Cicchetti, F. (2015). Cerebrovascular and blood-brain barrier impairments in Huntington's disease: Potential implications for its pathophysiology. *Annals of neurology*, *78*(2), 160-177. https://doi.org/10.1002/ana.24406

Etxeberria-Rekalde, E., Alzola-Aldamizetxebarria, S., Flunkert, S., Hable, I., Daurer, M., Neddens, J., & Hutter-Paier, B. (2020). Quantification of Huntington's Disease Related Markers in the R6/2 Mouse Model. *Front Mol Neurosci*, *13*, 617229. https://doi.org/10.3389/fnmol.2020.617229

Franciosi, S., Ryu, J. K., Shim, Y., Hill, A., Connolly, C., Hayden, M. R., McLarnon, J. G., & Leavitt, B. R. (2012). Age-dependent neurovascular abnormalities and altered microglial morphology in the YAC128 mouse model of Huntington disease. *Neurobiol Dis*, *45*(1), 438-449. https://doi.org/10.1016/j.nbd.2011.09.003

Mantovani, S., Gordon, R., Li, R., Christie, D. C., Kumar, V., & Woodruff, T. M. (2016). Motor deficits associated with Huntington's disease occur in the absence of striatal degeneration in BACHD transgenic mice. *Hum Mol Genet*, *25*(9), 1780-1791. https://doi.org/10.1093/hmg/ddw050

Menalled, L. B. (2005). Knock-in mouse models of Huntington's disease. *NeuroRx*, *2*(3), 465-470. https://doi.org/10.1602/neurorx.2.3.465

Slow, E. J., van Raamsdonk, J., Rogers, D., Coleman, S. H., Graham, R. K., Deng, Y., Oh, R., Bissada, N., Hossain, S. M., Yang, Y. Z., Li, X. J., Simpson, E. M., Gutekunst, C. A., Leavitt, B. R., & Hayden, M. R. (2003). Selective striatal neuronal loss in a YAC128 mouse model of Huntington disease. *Hum Mol Genet*, *12*(13), 1555-1567. https://doi.org/10.1093/hmg/ddg169

Smith, G. A., Rocha, E. M., McLean, J. R., Hayes, M. A., Izen, S. C., Isacson, O., & Hallett, P. J. (2014). Progressive axonal transport and synaptic protein changes correlate with behavioral and neuropathological abnormalities in the heterozygous Q175 KI mouse model of Huntington's disease. *Hum Mol Genet*, *23*(17), 4510-4527. https://doi.org/10.1093/hmg/ddu166

Southwell, A. L., Franciosi, S., Villanueva, E. B., Xie, Y., Winter, L. A., Veeraraghavan, J., Jonason, A., Felczak, B., Zhang, W., Kovalik, V., Waltl, S., Hall, G., Pouladi, M. A., Smith, E. S., Bowers, W. J., Zauderer, M., & Hayden, M. R. (2015). Anti-semaphorin 4D immunotherapy ameliorates neuropathology and some cognitive impairment in the YAC128 mouse model of Huntington disease. *Neurobiol Dis*, *76*, 46-56. https://doi.org/10.1016/j.nbd.2015.01.002

Spampanato, J., Gu, X., Yang, X. W., & Mody, I. (2008). Progressive synaptic pathology of motor cortical neurons in a BAC transgenic mouse model of Huntington's disease. *Neuroscience*, *157*(3), 606-620. https://doi.org/10.1016/j.neuroscience.2008.09.020

Tang, B., Seredenina, T., Coppola, G., Kuhn, A., Geschwind, D. H., Luthi-Carter, R., & Thomas, E. A. (2011). Gene expression profiling of R6/2 transgenic mice with different CAG repeat lengths

reveals genes associated with disease onset and progression in Huntington's disease. *Neurobiol Dis*, *42*(3), 459-467. https://doi.org/10.1016/j.nbd.2011.02.008

Yang, A. C., Stevens, M. Y., Chen, M. B., Lee, D. P., Stahli, D., Gate, D., Contrepois, K., Chen, W., Iram, T., Zhang, L., Vest, R. T., Chaney, A., Lehallier, B., Olsson, N., du Bois, H., Hsieh, R., Cropper, H. C., Berdnik, D., Li, L., . . . Wyss-Coray, T. (2020). Physiological blood-brain transport is impaired with age by a shift in transcytosis. *Nature*, *583*(7816), 425-430. https://doi.org/10.1038/s41586-020-2453-z
