# Supplementary material for: Huntington’s disease vasculopathy is barrier-type-selective and arteriolar-dominant with spatially-coupled astrogliosis and reversible by S1pr1 stimulation
Source: Nat Commun. 2026 Sep 12;17:10775. doi: 10.1038/s41467-026-77474-4 (PMC13661524; doi:10.1038/s41467-026-77474-4)
Supplement: Supplementary file 4 [file 41467_2026_77474_MOESM2_ESM.pdf]

Description of Additional Supplementary Files

Supplementary Videos

Supplementary Video 1: NaFluo paracellul ar leak into brain parenchyma at distinct cortical volumes, in WT, R6/2, and R6/2 treated mice (R6/2+). The data from all cortical volumes and blood was acquired simultaneously during the hyperstack (Z-stack over time) imaging. Time is relative to the NaFluo i.v. injection.

Supplementary Video 2: Dynamic AMT puncta (BSA-A488, green) visible at the blood-brain interface (at 120 min post injection).

Supplementary Video 3: Perivascular cells sequester albumin once it reaches the abluminal space, whether through increased BBB permeability in R6/2 mice, or in WT mice, after laser-induced extravasation used as a positive control. No significant presence and uptake of albumin is present in healthy, WT mice.

Supplementary Video 4: Timelapse recordings of cortical vessels dynamics. Upper panels show spontaneous constrictions of cerebral arterioles present in R6/2 mice (arrowheads). Lower panels show color-coded temporal maps of vessel diameter changes, evident in R6/2, but not in WT mice. In contrast, venules exhibit no constrictions, regardless of the genotype.

Supplementary Datasets

Supplementary Data 1. Summary of 2PM data and statistical analysis. Capital letters correspond to the tests referenced in the results section. Adjusted p value = p value after Bonferroni correction for testing multiple hypotheses. *=p<0.05; **=p<0.01; ***=p<0.001; ****=p<0.0001; ns=non-significant. Data provided as average ± SEM. Source data are provided in a Source Data file.

Supplementary Data 2. Summary of mice IHC data and statistical analysis. Capital letters correspond to the tests referenced in the results section. Adjusted p value = p value after Bonferroni correction for testing multiple hypotheses. *=p<0.05; **=p<0.01; ***=p<0.001; ****=p<0.0001; ns=non-significant. Data provided as average ± SEM. Source data are provided in a Source Data file.

Supplementary Data 3. Summary of mouse plasma and cerebrospinal fluid proteomic data and differential-abundance analysis. Protein intensities were $\log_2$-transformed and median-normalized after filtering and imputation. Differential abundance was assessed separately for each biofluid and pairwise comparison using two-sided independent-samples t-tests with permutation-based false-discovery-rate control (FDR < 0.05, S0 = 0.3, 250 randomizations). Run ID denotes an internal sequential analysis-row identifier; comparison specifies group 1 versus group 2; protein denotes the gene symbol or protein-group label; identifier denotes the UniProt accession; name gives the full protein name; status classifies

the protein as increased, decreased or unchanged according to the differential-abundance threshold and direction of change; sampled identifies the analyzed biofluid; mean (group 1) and mean (group 2) denote the mean normalized $\log_2$ protein intensity in the first and second groups named in the comparison, respectively; stdev (group 1) and stdev (group 2) denote the corresponding standard deviations; fold-change denotes the group 1/group 2 abundance ratio on the linear scale; $\log_2$FC denotes the $\log_2$ fold change, calculated as mean group 1 minus mean group 2, such that positive values indicate higher abundance in group 1 and negative values indicate lower abundance in group 1; P value adjusted denotes the exact permutation-based FDR-adjusted P value; and 'significant change' indicates whether the protein met the differential-abundance threshold. WT, wild type; R6/2, Huntington's disease-model mice; R6/2+, SEW2871-treated R6/2 mice; CSF, cerebrospinal fluid; FDR, false discovery rate; FC, fold change; SD, standard deviation; S0, parameter controlling the relative weighting of effect size in permutation-based FDR analysis. Data is also provided in a Source Data file.

Supplementary Data 4. Summary of electrophysiology data and statistical analysis. Capital letters (A, B, C, D) indicate the analyses cited in the Results. Effects were evaluated with a two-way mixed-effects model (REML), with group as the between-subject factor and stimulation frequency as the repeated within-subject factor, with measurements matched by mouse. The model tested group, frequency, and group × frequency effects. The omnibus effects were assessed using F tests. Detailed pairwise comparisons within each stimulation frequency were two-sided t-tests and adjusted for multiple comparisons using Šídák's method. Hash signs denote the relevant omnibus effect from the REML #=p<0.05; # # # =p<0.001; # # # # =p<0.0001; asterisks indicate results from Šídák-adjusted post hoc comparisons; *=p<0.05; **=p<0.01; ***=p<0.001; ****=p<0.0001; ns=non-significant. Exact P values, F and t statistics, and degrees of freedom are reported in the table. All Data is shown as average ± SEM. Source data are provided in a Source Data file.

Supplementary Data 5: Clinicopathological data for patients' post-mortem brain samples. HD status and Vonsattel grade denote HD presence and severity. Post-mortem interval (PMI) indicates the time from death to tissue preservation. RNA quality was measured using RNA Integrity Number (RIN) in the Frontal and Occipital poles. Cognitive Status describes function at death. Alzheimer's disease (AD) pathology includes: AD neuropathological change score (0=not, 1=low, 2=intermediate, 3=high); Braak stage (tau distribution); CERAD (Consortium to Establish a Registry for Alzheimer's Disease) plaque density score; Thal phase (amyloid-β spread); and CAA (Cerebral Amyloid Angiopathy) score for vascular amyloid. Lewy Body Disease (LBD) stages α-synuclein pathology. LATE (Limbic-predominant Age-related TDP-43 Encephalopathy) staging: −1=not assessed, 0=not identified, 1–3=increasing severity, 98=staging precluded. APOE genotype indicates Apolipoprotein E alleles. CTE (Chronic Traumatic Encephalopathy), ARTAG (Aging-Related Tau Astrogliopathy), and PART (Primary Age-Related Tauopathy) are aging-associated tauopathies (-1=Missing Information, 0=Not Identified 1=Possible, 2=Definite). FTLD-tau and FTLD-TDP are Frontotemporal Lobar Degeneration with tau or TDP-43 pathology, respectively. ALS/MND refers to Amyotrophic Lateral Sclerosis or Motor Neuron Disease. COVID-19 status as determined by PCR

and/or antibody testing. Individuals with dementia showed higher CAG repeats, lower brain weight, TDP-43,FTLD-TDP pathology, and more microinfarcts.

Supplementary Data 6. Summary of human IHC data and statistical analysis. Capital letters (A-U) correspond to the tests referenced in the results section. Adjusted p value = p value after Bonferroni correction for testing multiple hypotheses. *=p<0.05; **=p<0.01; ***=p<0.001; ****=p<0.0001; ns=non-significant. Data provided as average ± SEM. Source data are provided in a Source Data file.

Supplementary Data 7. Arteriolar GFAP+ burden relative to venules in HD patients. Cross-correlation between the CAG repeat length and ratio of GFAP+ cell count increase in arterioles over venules within corresponding patient cases. r = Pearson's correlation coefficient; p = two-sided probability under the null hypothesis of no linear correlation. Two cases were excluded because CAG-repeat information was unavailable.
